# Targeting USP1 Potentiates Radiation‐Induced Type I IFN‐Dependent Antitumor Immunity by Enhancing Oligo‐Ubiquitinated SAR1A‐Mediated STING Trafficking and Activation

**DOI:** 10.1002/advs.202412687

**Published:** 2025-02-20

**Authors:** Weilin Zhou, Yuxuan Zhao, Wenjing Qin, Weijian Wu, Chenyang Liao, Yiqiu Zhang, Xingli Yang, Xue Chen, Youqiao Wang, Yushan Kang, Jiaxin Wu, Jiaojiao Zhao, Junmin Quan, Xuecen Wang, Xianzhang Bu, Xin Yue

**Affiliations:** ^1^ School of Pharmaceutical Sciences Sun Yat‐Sen University Guangzhou Guangdong 510006 China; ^2^ The First Affiliated Hospital Jinan University Guangzhou Guangdong 510630 China; ^3^ Department of Radiation Oncology The First Affiliated Hospital Sun Yat‐sen University Guangzhou Guangdong 510080 China; ^4^ State Key Laboratory of Oncology in South China Guangdong Provincial Clinical Research Center for Cancer Sun Yat‐sen University Cancer Center Guangzhou Guangdong 510257 China; ^5^ Laboratory of Chemical Oncogenomics Guangdong Provincial Key Laboratory of Chemical Genomics Peking University Shenzhen Graduate School Shenzhen Guangdong 518072 China

**Keywords:** K27‐Ubn‐SAR1A, radiotherapy, STING trafficking and activation, targeting USP1, Type I IFNs

## Abstract

The magnitude of Type I interferon (IFN) mediated innate immune response within the tumor microenvironment (TME) critically influences the effectiveness of radiotherapy. Unfortunately, due to a myriad of resistance mechanisms, the double‐stranded DNA (dsDNA) signals produced by tumor cells postradiotherapy often induce a diminished response from immune cells. Through chemical screening targeting deubiquitinating enzymes, we identified USP1 (Ubiquitin Specific Peptidase 1) inhibitor as an enhancer of post‐radiotherapy dsDNA responses. Mechanistically, within the context of immune‐stimulatory cells in TME, USP1 serves as a suppressor in the stress‐mediated stages of the cGAS (Cyclic GMP‐AMP synthase) ‐STING (Stimulator of interferon genes protein) signaling pathway, specifically affecting the trafficking of STING from endoplasmic reticulum to Golgi apparatus. It is elucidated that SAR1A (Secretion associated Ras related GTPase 1A) requires K27‐linked oligo‐ubiquitination to assemble the STING‐COP‐II (Coat protein II) transport complex for STING trafficking. USP1 counteracts this activation by removing SAR1A ubiquitination, thereby blocking STING trafficking and activation. Consequently, pharmacological USP1 inhibition using ML323 sustains SAR1A ubiquitination and COP‐II complex formation, significantly enhancing STING trafficking and subsequent Type I IFN production. This intervention substantially amplifies radiotherapy‐induced immune activation in the TME, providing a strategic approach to overcome therapeutic resistance and synergize radiotherapy with immunotherapies.

## Graphical abstract

Targeting USP1 is identified as a strategy that significantly enhances type I IFN‐dependent innate immune response within the TME following radiotherapy. Mechanistically, USP1 inhibition facilitates STING trafficking from ER to Golgi by preserving K27‐linked oligo‐ubiquitination of SAR1A, thereby facilitating efficient activation of the dsDNA‐cGAS‐STING signaling pathway. This advancement significantly intensifies radiation‐induced “cold” to “hot” tumor conversion and strengthens antitumor immunity.

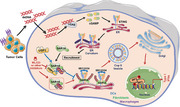

## 1 Introduction

Radiation therapy (RT) for tumors utilizes high‐energy X‐rays (or γ rays) to induce DNA damage in tumor cells, thereby achieving localized tumor ablation, this method continues to be one of the most effective clinical approaches for managing solid tumors, including nasopharyngeal carcinoma, early‐stage laryngeal cancer, cervical cancer, breast cancer, and rectal cancer.^[^ ^1^ ^]^ Nevertheless, RT still confronts numerous limitations, including insufficient efficacy, transient therapeutic effects, high recurrence rates, and inadequate control over tumor metastasis. These issues primarily emanate from radioresistance effects within tumor cells and the tumor microenvironment. It is evident that these shortcomings cannot be easily rectified through dosage adjustments or the indiscriminate combination with chemotherapy or immunotherapy.^[^ ^2^ ^]^ Therefore, developing targeted strategies to address these challenges is imperative to further augment the benefits of RT for patients. Increasing evidence suggests that the DNA damage and cytotoxic effects induced by RT, along with its immunomodulatory impact on the tumor microenvironment, are crucial for its antitumor response.^[^ ^3^ ^]^ Operationally, the prevailing view is that RT primarily triggers the release of proinflammatory mediators or damage‐associated molecular patterns (DAMPs), which enhance the innate immune responses of antigen‐presenting cells (APCs) such as dendritic cells (DCs) and other immune‐stimulatory cells, promoting their activation and infiltration.^[^ ^4^ ^]^ This process augments the cross‐presentation of tumor antigens, thereby modulating tumor immunogenicity, a phenomenon often summarized as RT converting the tumor immune status from “cold” to “hot.”^[^ ^5^ ^]^ However, clinical practice has revealed that the immune response triggered by RT is typically modest, indicating the general inefficacy of RT in inducing a robust “cold‐to‐hot” immunogenic shift in tumors.^[^ ^6^ ^]^ Consequently, understanding the inhibitory factors impeding the “cold‐to‐hot” transition induced by RT and devising targeted strategies to “awaken” the immune response is essential.

RT induces double‐strand breaks (DSBs) in DNA, leading to the liberation of significant amounts of double‐stranded DNA (dsDNA) within tumor cells, subsequently released into the tumor microenvironment (TME). This provides the material basis for dsDNA‐mediated, type I interferon (IFN)‐dependent innate immune responses. Notably, the absence of innate immune signals within the TME is a principal cause of the “cold” tumor phenotype. Therefore, we hypothesize that dsDNA‐mediated innate immune responses are a pivotal step in RT‐induced conversion of tumors from “cold” to “hot.” The cyclic GMP‐AMP synthase‐Stimulator of interferon response cGAMP interactor 1 (cGAS‐STING) pathway plays an essential role in sensing intracellular dsDNA signals and activating type I IFN‐dependent innate immune responses.^[^ ^7^ ^]^ Consequently, our research focus shifts to examining dsDNA‐cGAS‐STING signal pathway within the TME following RT, exploring the principal inhibitory factors influencing the initiation of innate immune responses from a target discovery perspective. The dynamic equilibrium between ubiquitination and deubiquitination in the regulation of innate immune responses has garnered significant attention.^[^ ^8^ ^]^ Recent studies have increasingly highlighted the indispensable role of deubiquitinating enzymes (DUBs) in preserving the homeostasis and functionality of critical immune checkpoints and regulatory factors within immune cells, thus earning them the appellation of “secondary immune checkpoints.”^[^ ^8^, ^9^ ^]^ Accordingly, in this research, we developed a screening model predicated on DUB inhibitors: DC cells were subjected to chemical intervention and subsequently coincubated with irradiated tumor cells to identify inhibitors that effectively augment the innate immune response of DC cells. Validation indicated that targeting and inhibiting ubiquitin specific peptidase 1 (USP1) significantly enhanced the innate immune response within the TME following RT. This provided a crucial direction for the research. Follow‐up experiments excluded the involvement of dsDNA release, uptake, and cGAS sensing, synthesis, and functionality. Ultimately, it was confirmed that targeting USP1 substantially augmented the ability of STING to translocate from the endoplasmic reticulum (ER) to the Golgi apparatus and activate.

The STING‐mediated release of type I IFNs relies on the involvement of various auxiliary factors. Upon the ER membrane, the binding of cyclic GMP‐AMP (cGAMP) to STING triggers a conformational change, resulting in its aggregation. Through interactions with regulatory proteins, such as yip1 domain family member 5, transmembrane emp24 domain‐containing protein 2/5, inactive rhomboid protein 2, or STING ER exit protein (STEEP), the aggregated STING is transported from ER to Golgi apparatus, encapsulated within coat protein complex II (COP‐II) vesicles.^[^ ^10^ ^]^ Subsequently, this aggregated STING adheres via glycosaminoglycans to the trans‐face of the Golgi apparatus, facilitating the activation of TANK Binding Kinase 1 (TBK1), and the subsequent recruitment and activation of interferon regulatory factor 3 (IRF3).^[^ ^11^ ^]^ The COP‐II‐mediated STING transport step represents the primary rate‐limiting phase in the activation of the STING pathway. COP‐II is composed of an inner layer formed by the small GTPase secretion‐associated and Ras‐related protein 1 (SAR1), protein transport protein Sec23 (SEC23), and protein transport protein Sec24 (SEC24), alongside an outer layer of protein transport protein Sec13 and protein transport protein Sec31 . Studies have shown that the knockout of any of these components hinders STING transport.^[^ ^11^, ^12^ ^]^ Consequently, this step is likely the critical juncture for the action of key inhibitory factors in the STING activation process. In this study, we present for the first time that, in cells susceptible to innate immune response, K27‐type oligo‐ubiquitinated secretion associated Ras related GTPase 1A (SAR1A) constitutes the active form of SAR1A involved in the assembly of the STING‐COP‐II transport complex. Concurrently, USP1 acts as a regulatory factor that can promptly recognize and deactivate this active form through deubiquitination, thereby obstructing transport complex formation and causing STING retention on the ER. Notably, such susceptible cells, including DCs, fibroblasts, or macrophages, are vital components of the TME. Therefore, we utilized a targeted inhibitor strategy that effectively enhances the STING‐mediated innate immune response in tumors post‐RT, significantly improving the transition of tumors from “cold” to “hot” following RT. This not only facilitates the abscopal effect of RT but also presents an excellent opportunity for employing immunotherapeutic agents, such as programmed cell death 1 ligand 1 (PDL1) monoclonal antibodies. Furthermore, analyses of clinical specimens of colorectal cancer indicate that the expression levels of USP1 in nontumor cells within the tumor pre‐RT correlate with the degree of immune activation and therapeutic efficacy post‐RT. This underscores the potential of targeting USP1 as a significant approach to enhance RT outcomes.

## 2 Results

### 2.1 Identification of USP1 Inhibitors Augments Type I IFNs Induction and Strengthens Innate Immune Responses Following RT

**Figure 1:**
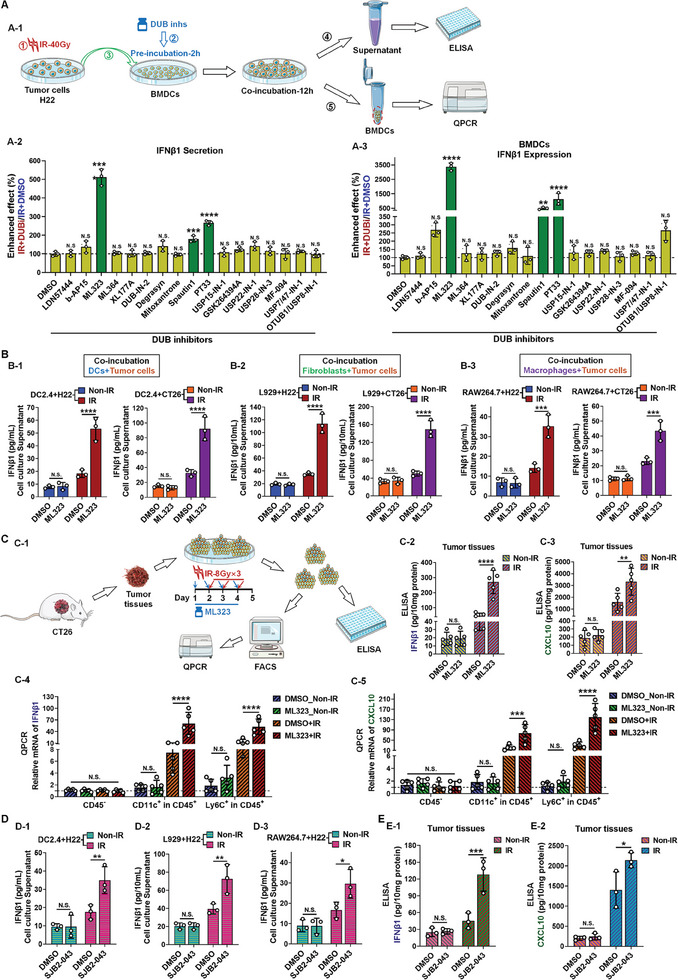
Screening and identification of USP1 inhibitors as key factors to enhance innate immune effects following RT. A) in vitro coincubation screening: A‐1) Schematic diagram of in vitro coincubation experiment: Coculture of tumor cells with BMDCs after RT, followed by ELISA and RT‐qPCR assay. A‐2) Quantification of IFNβ1 secretion in the supernatant of co‐cultured BMDCs treated with various DUB inhibitors using ELISA assay. A‐3) Expression levels of IFNβ1 in cocultured BMDCs measured by RT‐qPCR. Compared with sample treated with DMSO group, data indicate increase degree with combination DUB inhibitors treatment. B) Effects of USP1 inhibitor ML323 on IFNβ1 secretion in coculture experiments of DC2.4 B‐1), L929 B‐2), and RAW264.7 B‐3) cells with irradiated H22 and CT26 cells. Cotreatment with ML323 showed elevated secretion levels compared to the RT group. C) Effects of ML323 on IFNβ1 and CXCL10 secretion levels of DC or macrophage cells: C‐1) Schematic diagram; C‐2, C‐3) IFNβ1 and CXCL10 secretion levels in the indicated treatment group of mouse tumor tissues was detected. Single‐cell suspensions from tumors treated with C‐1 were stained with conjugated antibodies against CD45, CD11c, and Ly6C, followed by sorting into different cell populations using flow cytometry. The mRNA levels of IFNβ1 C‐4) and CXCL10 C‐5) in various cell subsets were then quantified via RT‐qPCR. D) Effects of USP1 inhibitor SJB2‐043 on IFNβ1 secretion in coculture experiments of DC2.4 D‐1), L929 D‐2), and RAW264.7 D‐3). Cotreatment with SJB2‐043 showed elevated secretion levels compared to the RT group. F) IFNβ1 F‐1) and CXCL10 F‐2) secretion levels in the indicated treatment group of mouse tumor tissues was detected. All data were represented as mean ± S.D. (*n* ≥ 3). Statistical significance was determined by A2 and A3) one‐way ANOVA, B‐1/2/3, C‐2/3/4/5, D‐1/2/3/4, and F‐1/2) two‐way ANOVA (N.S., no significance; *, *P* < 0.05; **, *P* < 0.01; ***, *P* < 0.001; ****, *P* < 0.0001).

**Figure 2:**
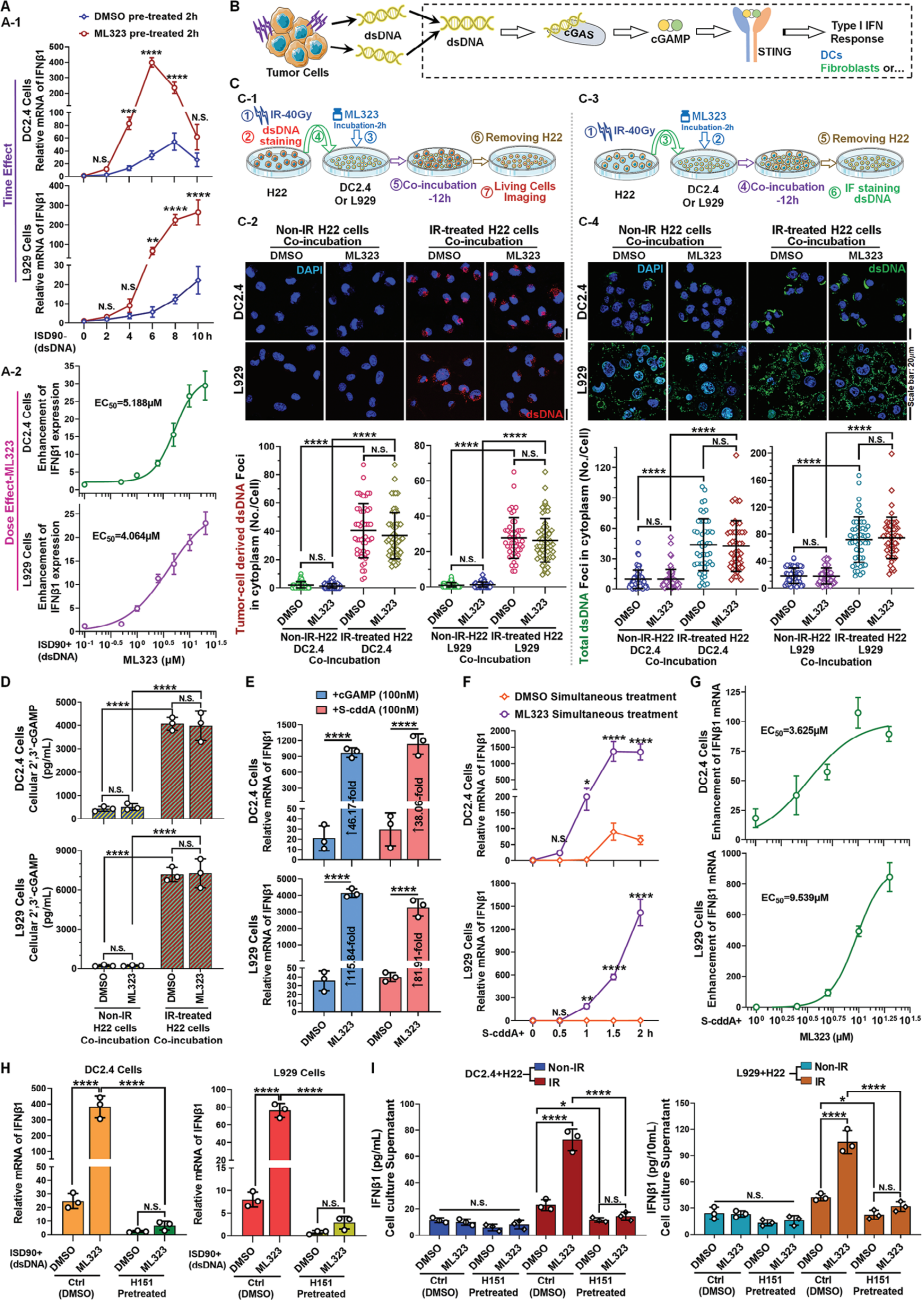
The effects of the USP1 inhibitor ML323 on type I IFN responses through the STING pathway. A) Time‐course analysis of IFNβ1 expression in DC2.4 and L929 cells treated with dsDNA, with DMSO or ML323 pretreatment. B) Schematic representation of the cGAS‐STING pathway activation by tumor released dsDNA leading to type I IFN production in innate immunity cells. C) Characterization of tumor cell‐derived dsDNA and total dsDNA content and distribution: C‐1) Experimental flowchart of coincubation and live‐cell imaging for prelabeled dsDNA in H22 cells. C‐2) Imaging of dsDNA content and distribution within DC2.4 and L929 cells after coincubation with H22‐derived dsDNA in live cells. C‐3) Experimental flowchart for coincubation and IF imaging of H22 cells with DC2.4 and L929 cells after RT. C‐4) IF assay analysis of dsDNA content and distribution within DC2.4 and L929 cells after coincubation. D) ELISA quantification of 2′3’‐cGAMP levels in DC2.4 and L929 cells after coincubation experiment. E) IFNβ1 expression levels in DC2.4 or L929 cells were treated with ML323 (5 µm) in the presence of 2′3’‐cGAMP or S‐cddA. F) Time‐course analysis of IFNβ1 expression in DC2.4 and L929 cells, when treated with ML323 (5 µm) under S‐cddA stimulation (100 nm). G) Dose‐response analysis of ML323 on IFNβ1 expression in DC2.4 and L929 cells, treated with ML323 at indicated concentrations under S‐cddA stimulation (100 nm). H) The mRNA levels of IFNβ1 in DC2.4 and L929 cells treated with dsDNA (5 nm) plus ML323 (5 µm) after pre‐incubation with H151 (5 µm). I) ELISA quantification of IFNβ1 secretion by DC2.4 and L929 cells in coculture experiments demonstrated that cotreatment with ML323, following preincubation with H151, resulted in IFNβ1 secretion levels. Data were represented as mean ± S.D. (*n* ≥ 3). The statistical method for cell imaging includes results from all biological replicates (*n* = 3). Statistical significance was determined by (A, C, D, E, F, H, and I) two‐way ANOVA (N.S., no significance; *, *P* < 0.05; **, *P* < 0.01; ***, *P* < 0.001; ****, *P* < 0.0001).

Following RT, innate immune cells within the TME, such as DCs, fibroblasts, and certain monocyte‐macrophages, sense DAMP signals released by tumor cells and respond by expressing and secreting type I IFNs, thereby initiating an immune response within the tumor. As previously noted, DUBs are likely to function as stress‐induced suppressors in this process, consequently inhibiting the immune activation effects elicited by RT at their source. DUBs, as a class of functional enzymes, typically exert their influence through deubiquitination. Accordingly, we designed in vitro coincubation experiments to evaluate whether various DUB inhibitors have the potential to enhance dsDNA responses (Figure S1A, Supporting Information), with the aim of identifying specific inhibitory DUBs that could be therapeutically targeted, this approach serves a dual purpose. We irradiated tumor cells (H22) with high‐intensity radiation and subsequently coincubated them with primary bone marrow‐derived DC cells (BMDCs) to assess the expression and secretion of Type I IFNs (IFNβ1) in BMDCs (**Figure** **1**A‐1). The results revealed that the USP1 inhibitor ML323, the ubiquitin specific peptidase 13 inhibitor Spautin1, and the ubiquitin specific peptidase 14 inhibitor PT33 all significantly enhanced the expression and secretion of IFNβ1 (Figure 1A‐2/3; and Figure S1B,C, Supporting Information). The enhancement mechanisms mediated by Spautin1 and PT33 within the Type I IFN signaling pathway have been previously documented.^[^ ^13^ ^]^ However, the enhancement effect resulting from the inhibition of USP1 by ML323 is a novel finding from our study, demonstrating the most pronounced effect at equivalent concentrations (Figure 1A‐2/3; and Figure S1B,C, Supporting Information). Consequently, we intend to further investigate the effects and underlying mechanisms of ML323.

**Figure 3:**
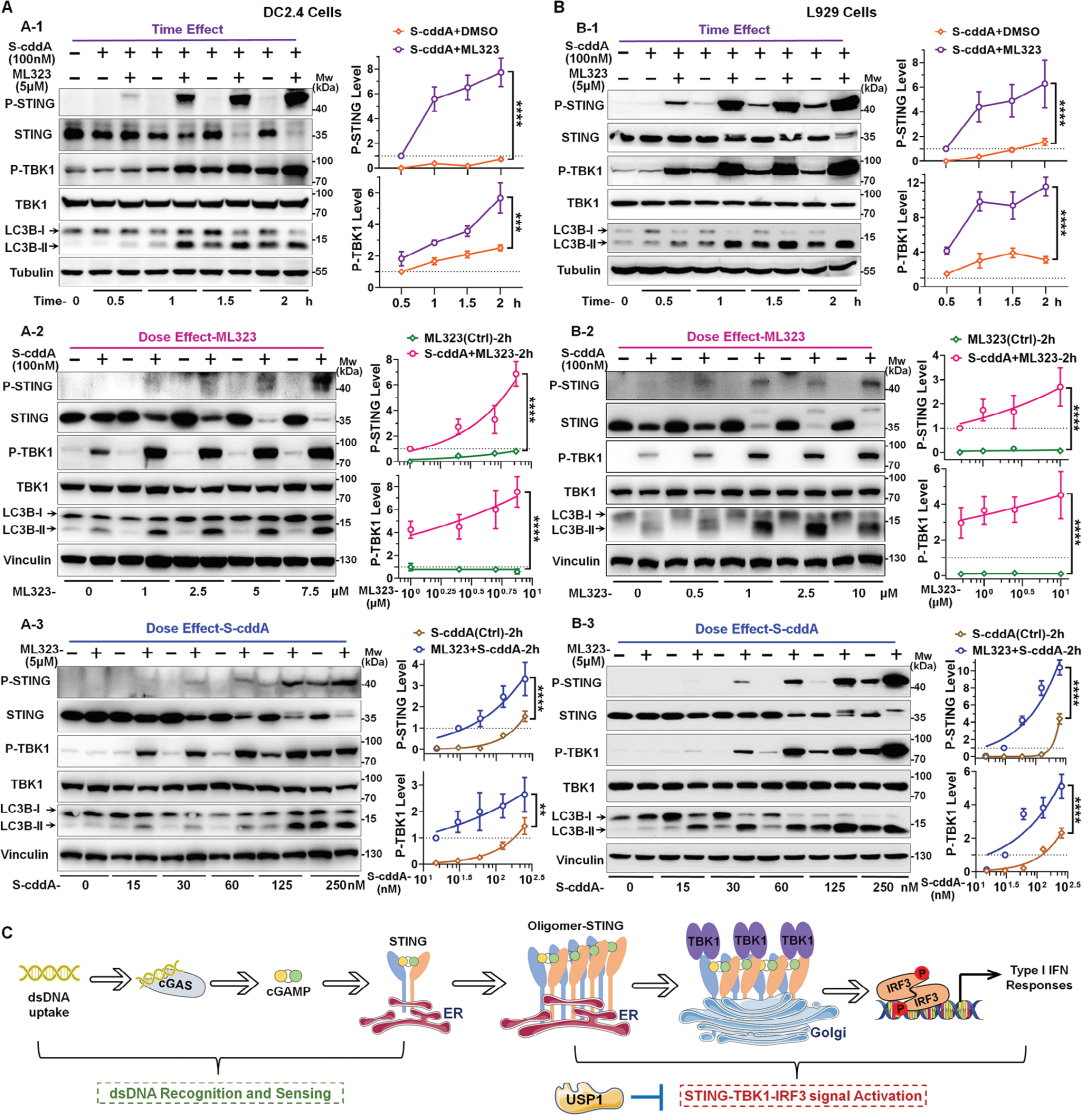
Inhibition of USP1 with ML323 enhances STING pathway activation and promotes LC3 lipidation. WB analysis of DC2.4 A) and L929 B) cells treated with ML323 and S‐cddA demonstrated alterations in STING signaling and changes in the LC3B‐II/LC3B‐I ratio. Time‐course analysis of S‐cddA alone or in combination with ML323 in DC2.4. A‐1) and L929 B‐1) cells revealed variations in P‐STING, P‐TBK1, and LC3B‐II/LC3B‐I levels. Dose‐response analysis of ML323 in DC2.4 A‐2) and L929 B‐2) cells showed a dose‐dependent increase in P‐STING, P‐TBK1, and LC3B‐II/LC3B‐I levels when combined with S‐cddA. Dose‐response analysis of S‐cddA in DC2.4 A‐3) and L929 B‐3) cells revealed a dose‐dependent increase in P‐STING, P‐TBK1, and LC3B‐II/LC3B‐I levels when combined with ML323.Quantifications of grayscale values (right), data were represented as mean ± S.D. (*n* = 3). Statistical significance was determined by two‐way ANOVA (**, *P* < 0.01; ***, *P* < 0.001; ****, *P* < 0.0001). C) The mechanistic model diagram illustrating the inhibitory role of USP1 in the STING signaling pathway.

**Figure 4:**
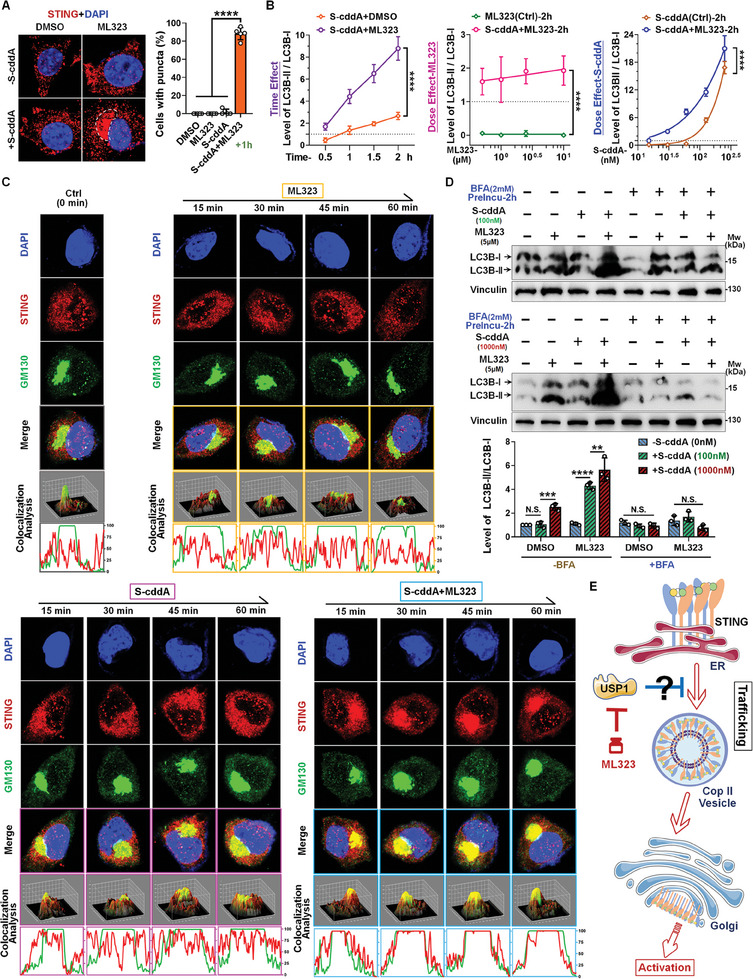
Targeting USP1 with ML323 facilitates STING trafficking from ER to ERGIC/Golgi. A) IF assay indicates co‐treated with ML323 + S‐cddA (1 h) significantly promoted STING aggregation in L929 cells. The proportion of cells with puncta was calculated by counting all cells in the field of view and then counting the percentage of cells with obvious STING aggregation. Left: representative images; right: statistician of cell with puncta. B) WB analysis of the gray values of LC3B‐II/LC3B‐I in the time‐point effect and dose response of S‐cddA or ML323 with combination treatment in L929 cells (shown in Figure 3). C) IF assay to observe the distribution and colocalization of STING and GM130, with increased colocalization of STING and GM130 in cells treated with S‐cddA, ML323, or combination. Upper: representative images; below: fluorescence colocalization analysis. D) Observation of BFA's impact on STING transport: Upon stimulation with varying concentrations of S‐cddA, grayscale value analysis reveals the LC3B‐II levels in L929 cells subjected to S‐cddA, ML323, or a combination treatment, following pretreatment with BFA. Data were represented as mean ± S.D. (*n* ≥ 3). Statistical significance was determined by A) one‐way ANOVA. B,D) Two‐way ANOVA (N.S., no significance; *, *P* < 0.05; **, *P* < 0.01; ***, *P* < 0.001; ****, *P* < 0.0001). E) The mechanistic model diagram illustrating the main role of USP1 is to inhibit the transport of STING from the endoplasmic reticulum to the Golgi apparatus.

Subsequently, we broadened the scope of effector cells in our in vitro coincubation experiments to include DCs, fibroblasts, and macrophages to further substantiate the augmentative effect of ML323. The findings revealed that following coincubation with irradiated H22 cells, DC2.4 cells (a murine dendritic cell line), L929 cells (a murine fibroblast cell line), and RAW264.7 cells (a murine macrophage cell line) all exhibited an enhanced capacity for IFNβ1 production and secretion to varying extents. The addition of ML323 markedly intensified this effect; however, this enhancement was exclusively observed when cells were coincubated with irradiated H22 cells, implying that the efficacy of ML323 is contingent upon RT as a prerequisite trigger (Figure 1B‐1/2/3; and Figure S2, Supporting Information). Moreover, when the tumor cells were replaced with CT26 cells, ML323 similarly elicited a significant increase in the secretion of IFNβ1 across all 3 cell lines post‐RT (Figure 1B‐1/2/3). It is noteworthy that ML323's enhancement of IFNβ1 production and secretion was not evident in tumor cells themselves, even under various irradiation protocols (Figure S3A–D, Supporting Information), indicating a lack of direct effect on tumor cells. To further corroborate these findings, we investigated whether ML323 could promote the secretion or release of type I IFNs within tumor tissues in vivo. Specifically, we excised CT26 tumors from mice, sectioned them into uniform tissue blocks (40–60 mm^3^), and measured IFNβ1 levels in the tumor tissues following in vitro intervention (Figure 1C‐1). The results demonstrated that ML323 increased IFNβ1 levels by up to fivefold in irradiated tumor tissues, with this effect being exclusively observable in post‐RT tumor tissues (Figure 1C‐2). Additionally, we observed a significant upregulation in the secretion of C‐X‐C motif chemokine ligand 10 (CXCL10), a crucial downstream mediator of type I IFNs, under the influence of ML323 (Figure 1C‐3). The evaluation of the Type I interferon response in tumor tissue and its secretion into the TME in this study focused on the activation of DCs or macrophages. Post‐treatment tumor tissues were analyzed by isolating cells using flow cytometry and measuring the mRNA expression levels of IFNβ1 and CXCL10 to assess their activity. The results demonstrated that CD11c^+^ DCs exhibited a significantly enhanced capacity to produce IFNβ1 and CXCL10 when treated with ML323, compared to the RT‐only group (Figure 1C‐4/5; and Figure S4A, Supporting Information). The activation of CD11c^+^ cells serves as a critical indicator of innate immune activation within the tumor following RT.^[^ ^14^ ^]^ Additionally, the Lymphocyte antigen 6 complex^+^ (Ly6C^+^) phenotype was used to characterize monocyte expression, including macrophages.^[^ ^14^ ^]^ Consistent with the DC findings, Ly6C^+^ monocytes also showed significant enhancement in response to ML323 (Figure 1C‐4/5; and Figure S4B, Supporting Information). These results highlight that ML323 amplifies the Type I interferon response of DCs and macrophages in tumors after RT.

Moreover, we also noted that the reported USP1 inhibitors are not confined to those exemplified by ML323; another class of inhibitors with distinctly different structural characteristics can also target USP1.^[^ ^15^ ^]^ Consequently, we chose SJB2‐043 as a corroborative molecule. In both in vitro coincubation assays and murine tumor tissue intervention studies, SJB2‐043 exhibited a capacity to enhance the innate immune response following RT, albeit with less efficacy compared to ML323 under equivalent treatment conditions (Figure 1E). Taken together, these findings indicate that USP1 inhibitor markedly enhances the innate immune response within tumor tissues following RT and suggest that targeting USP1 may substantially influence the immunophenotypic remodeling of the tumor microenvironment post‐RT.

### 2.2 Targeting and Inhibiting USP1 Enhances Radiation‐Induced dsDNA Sensing via the STING Pathway

Radiation‐induced free radicals penetrate the nucleus or mitochondria, resulting in DSBs in DNA, which represent the primary biological impact of radiation on cells. DNA‐DSBs trigger the release of significant quantities of dsDNA both within tumor cells and into the TME.^[^ ^16^ ^]^ Consequently, it can be inferred that the release and uptake of dsDNA form the essential material basis for the activation of innate immune signaling within the tumor post‐RT. Based on this, it is imperative to first confirm whether the amplified effect of ML323 targeting USP1 on the tumor's innate immune response post‐RT stems from the dsDNA signaling pathway. To elucidate this, we assessed the impact on type I IFN expression and the activation of downstream pathways by transfecting cells to simulate dsDNA uptake. Time‐course experiments revealed that in both DC2.4 and L929 cells, ML323 exhibited a pronounced enhancement as early as 2 h post‐dsDNA uptake. DC2.4 cells displayed a more rapid pathway response, with the ML323 group peaking at ≈6 h, markedly earlier than the DMSO group, with a peak nearly tenfold higher. Although L929 cells exhibited a more sluggish overall response to dsDNA, the ML323 group not only demonstrated a significant enhancement effect but also shortened the response duration (**Figure** **2**A‐1). The dose‐dependent increase in IFNβ1 expression induced by ML323 was also notably apparent, with an EC_50_ value around 5 µm (Figure 2A‐2). Regarding downstream effects, ML323 significantly upregulated CXCL10 expression, aligning with the pattern observed in IFNβ1 expression (Figure S5A,B, Supporting Information). These findings indicate that the augmentation of dsDNA signaling mediated by ML323 is the primary mechanism through which it enhances the innate immune response within the tumor.

The cGAS‐STING pathway is crucial for detecting intracellular dsDNA signals and initiating type I IFN‐dependent innate immune responses.^[^ ^17^ ^]^ To further discern the precise stage at which ML323 targeting USP1 disrupts stress‐induced inhibitory factors, we will undertake a stepwise validation approach (Figure 2B). First, the issue of verifying the source of dsDNA must be addressed. It has been confirmed that a primary mechanism by which cells release dsDNA involves transportation and intercellular interaction via exosomes or other extracellular vesicles.^[^ ^18^ ^]^ Based on this understanding, we measured the dsDNA content in exosomes released by H22 and CT26 cells before and after RT. The results demonstrated that tumor cells release a substantial amount of dsDNA into the extracellular environment through exosomes following RT (Figure S6, Supporting Information). To further investigate whether innate immune cells, such as DCs, can take up dsDNA released by tumor cells, we utilized two characterization approaches. After RT, dsDNA within tumor cells was labeled, the labeled tumor cells were then cocultured with DC2.4, L929, and RAW264.7 cells (Figure 2C‐1). The results revealed that all three cell types were capable of uptaking dsDNA released by irradiated tumor cells. Importantly, this uptake was independent of whether the cells were preincubated with ML323 (Figure 2C‐2; and Figure S7A/C, Supporting Information). Furthermore, immunofluorescence (IF) analysis was performed to assess the total dsDNA content in cells after cocultivation (Figure 2C‐3). The analysis revealed a significant increase in the total dsDNA content in DC2.4, L929, and RAW264.7 cells following coculture with irradiated tumor cells, again independent of ML323 treatment (Figure 2C‐4; and Figure S7B/D, Supporting Information). In summary, these findings indicate that DC cells or other innate immune cells possess the ability to uptake dsDNA released by tumor cells post‐RT, potentially contributing to the activation of the STING pathway. Notably, ML323 does not influence the uptake of tumor cell‐released dsDNA by DC or other immune cells, as its mechanism of action does not interfere with this process.

Subsequently, we evaluated the cGAS‐mediated synthesis of cGAMP and found that following the internalization of dsDNA released from tumor cells after RT, the cGAMP levels within L929 cells markedly increased. However, ML323 did not affect the cGAMP synthesis at the stage where cGAS detects dsDNA (Figure 2D). Naturally, we postulated that ML323's mechanism of action likely intervenes during the activation of STING. Indeed, ML323 markedly amplifies the potency of cGAMP or the STING agonist S‐cddA (a cGAMP analogs, Figure S8A, Supporting Information) in inducing IFNβ1 expression (Figure 2E; and Figure S8B, Supporting Information). Pursuing this hypothesis, we undertook a thorough evaluation of ML323's efficacy, to optimize the experimental process, we employed S‐cddA directly to trigger STING activation. The time‐course analysis revealed that the enhancement effect mediated by ML323 becomes apparent shortly after S‐cddA addition (within 0.5–1 h). This pronounced effect fully develops over time, peaking within 1.5–2 h. Notably, the concentration of S‐cddA employed did not elicit activation effects in L929 and RAW264.7 cells; however, the introduction of ML323 led to a transition from inactivity to significant activation (Figure 2F; and Figure S8C, Supporting Information). The dose‐response analysis further indicated that as the concentration of ML323 increased, its capacity to promote IFNβ1 expression correspondingly escalated, with maximal activation observed at concentrations between 10 and 20 µm (activation plateau) (Figure 2G; and Figure S8D, Supporting Information). Subsequently, we assessed the expression of the downstream factor CXCL10, which revealed that the enhancement effect induced by ML323 was also profoundly significant, this effect manifested later than the IFNβ1 expression (Figure S8E, Supporting Information). And the dose‐response analysis clearly demonstrated the dose‐dependent efficacy of ML323 (Figure S8F, Supporting Information). Additionally, we substituted the STING agonist with a non‐nucleoside STING activator‐DMXAA and likewise observed a pronounced enhancement effect mediated by ML323 (Figure S9A, Supporting Information). Simultaneously, the dose‐dependent efficacy of another USP1 inhibitor, SJB2‐043, was also clearly demonstrated (Figure S9B, Supporting Information).

To further validate the conclusion that STING activation constitutes the principal mechanism of action for ML323, we emulated the post‐RT mode of action by substituting the activating factor at its origin with dsDNA transfection and an in vitro coincubation system. We employed the STING inhibitor H151 to obstruct the activation phase and assess the impact of ML323. The findings revealed that the augmentation effect of ML323 on IFNβ1 expression following ISD90 transfection was abolished upon the introduction of H151 (Figure 2H). Similarly, the enhanced effect observed after coincubation with irradiated tumor cells was nullified with the addition of H151 (Figure 2I). These results suggest, first, that the action of ML323 indeed takes place subsequent to the dsDNA response signal, and second, that the precise point of action is at the level of STING activation. Collectively, the critical mechanism by which ML323 targets USP1 to amplify the type I IFN pathway response is through its facilitation of STING activation.

### 2.3 Blockade of USP1 Potentiates STING Trafficking and Activation

Next, we will explore the mechanism by which ML323 targets USP1 to enhance STING pathway activation. Our first step involves a comprehensive evaluation of the effects of ML323 on STING pathway activation. It is well established that STING undergoes several critical stages during activation: cGAMP induces STING aggregation, which in turn drives its trafficking from the ER to the Golgi apparatus. Upon aggregation, STING anchors at the mature face of the Golgi, where it recruits the core activator TBK1. TBK1 forms a dimeric structure, with the monomers mutually activating one another, leading to the subsequent phosphorylation of STING. The activated STING then plays a pivotal role in the activation of downstream IRF3.^[^ ^19^ ^]^ Accordingly, assessing the activation status of STING and the activity of TBK1 will elucidate the mechanism by which ML323 enhances pathway activation. Using S‐cddA as a stimulus, our results indicated that ML323 completely reversed the ineffectiveness or low efficacy of S‐cddA alone in all three cell types, demonstrating a significant, time‐dependent increase in STING and TBK1 phosphorylation levels, thus clearly promoting activation (**Figure** **3**A‐1,B‐1; and Figure S10A, Supporting Information). Furthermore, dose‐response characterization of ML323 revealed a clear dose‐dependent enhancement, with a marked effect observed at concentrations as low as 0.5–1 µm, and substantial enhancement at concentrations exceeding 2.5 µm (Figure 3A‐2,3B‐2; and Figure S10B, Supporting Information). Additionally, in the dose‐response results of S‐cddA, ML323 exhibited a potent synergistic effect; even at high concentrations where S‐cddA alone demonstrated some activation, the addition of ML323 significantly amplified this activation (Figure 3A‐3,B‐3; and Figure S10C, Supporting Information). Notably, these time‐course and dose‐course results confirmed that ML323 alone cannot activate the STING pathway (Figure 3A‐2,3B‐2; and Figures S10B and S11A–C, Supporting Information). Together, ML323 demonstrates a strong ability to enhance STING signaling activation in the presence of STING agonist, positioning it as an effective auxiliary factor in the activation of the STING pathway (Figure 3C).

The formation of STING puncta serves as crucial evidence of STING's self‐regulation at distinct stages of its activation. Having confirmed the effects of ML323 on STING pathway activation, we aim to progressively elucidate its specific mechanisms of action. Using immunofluorescence (IF) assays, we initially observed that ML323 markedly enhances STING aggregation induced by S‐cddA (**Figure** **4**A; and Figure S12A, Supporting Information), suggesting that ML323's effects are predominantly focused on STING's self‐regulation. Furthermore, in the previously mentioned time‐ and dose‐dependent experiments, we discovered that ML323 significantly promotes the conversion of microtubule‐associated proteins 1A/1B light chain 3 (LC3) to its lipidated form (LC3‐I → LC3‐II), a process mediated by S‐cddA (Figure 4B; and Figure S12B, Supporting Information). This lipidation of LC3 represents a critical step in autophagosome biogenesis.^[^ ^20^ ^]^ These results indicate that ML323 facilitates STING's transport to the ER‐Golgi intermediate compartment (ERGIC), thereby potently inducing LC3‐lipidation. Notably, Within our 2‐h observation window, only LC3 lipidation was detected, while autophagic activity following autophagosome formation was not observed (Figure S12C, Supporting Information), this indicates that after STING activation, it promotes LC3 lipidation during the autophagy process, which is consistent with the reported effects resulting from STING transport.^[^ ^11a^ ^]^ Additionally, the enhancement of STING translocation by ML323 was also observed through IF experiments (Figure S13, Supporting Information). These findings imply that the mechanism by which ML323 targets USP1 is likely involved in promoting STING translocation. Subsequently, we performed a thorough evaluation of the STING translocation process from ER to the Golgi apparatus. Our initial Golgi colocalization analysis demonstrated that ML323 not only reverses the ineffectiveness of S‐cddA at this concentration in facilitating STING translocation but also markedly accelerates this process. Remarkably, within merely 15 min of treatment, a significant aggregation of STING at the Golgi apparatus was evident, with the degree of colocalization (indicative of aggregation) progressively increasing over time (Figure 4C; and Figure S14, Supporting Information). Concurrently, the colocalization effect between STING and TBK1 was also substantially amplified in response to ML323 stimulation (Figure S15, Supporting Information).

Upon cGAMP binding on the ER membrane, STING undergoes a conformational change, leading to its aggregation. This aggregated STING, encapsulated by the COP‐II coat protein complex, forms a transport vesicle that facilitates its translocation from the ER to the Golgi apparatus.^[^ ^10a^ ^]^ To further clarify the mechanism, we employed Brefeldin A (BFA), an inhibitor of the GTPases necessary for COP‐II complex formation, to block the formation of transport vesicles, and subsequently observed whether the enhancement effect mediated by ML323 was affected. We monitored the formation of LC3B‐II as an indicator of STING translocation. The results demonstrated that the addition of BFA completely abolished the potentiating effect of ML323, and even a substantial increase in the concentration of S‐cddA failed to induce the conversion of LC3B‐I to LC3B‐II across the groups (Figure 4D), this further emphasizes that ML323's targeting of the regulatory pathway involving USP1 is intricately linked to the translocation process. Collectively, these findings suggest that ML323 significantly enhances STING trafficking to the Golgi apparatus (Figure 4E), and its ability to promote downstream signal activation is predominantly achieved through this facilitated translocation.

### 2.4 USP1 Targets and Removes K27‐Linked Oligo‐Ubiquitination from SAR1A, Resulting in the Functional Inactivation of SAR1A

**Figure 5:**
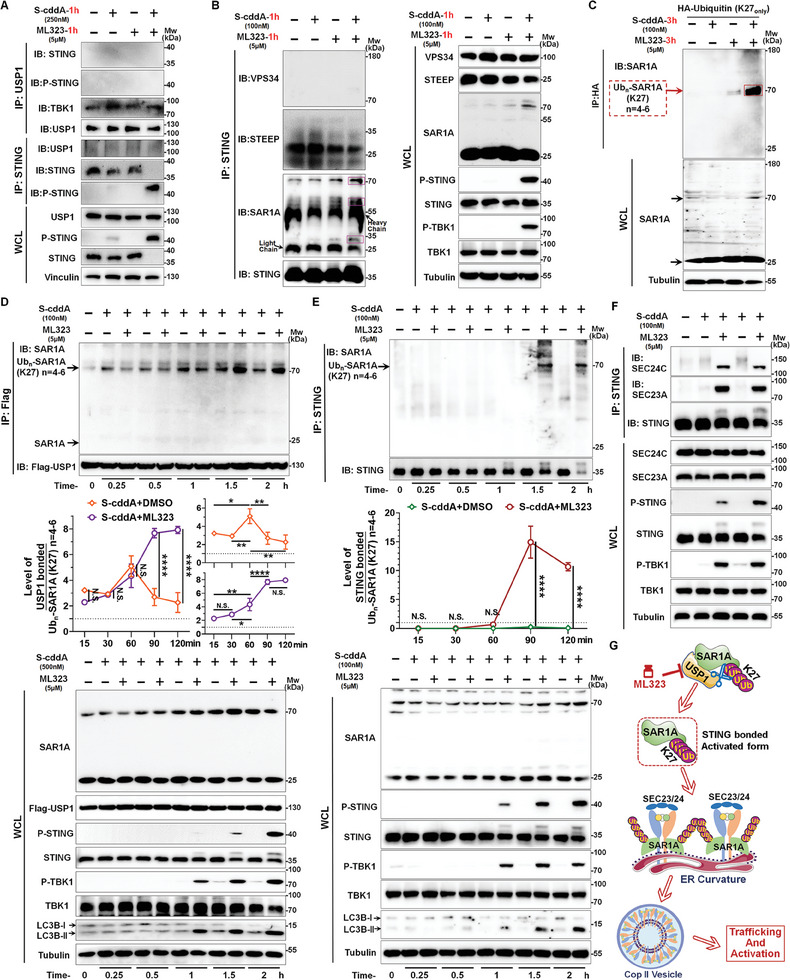
USP1 targets and deubiquitinated K27‐linked oligo‐ubiquitinated SAR1A. A) Co‐IP/WB analysis was used to detect the interaction between STING and USP1 in L929 cells. B) Co‐IP/WB assay was used to detect the interaction between STING and VPS34, STEEP, SAR1A in L929 cells. C) Co‐IP/WB assay was used to detect the ubiquitinated form of SAR1A in L929^HA‐UB‐K27^ cells. D) Co‐IP/WB assay was used to detect the time‐effect of interaction between USP1 and oligo‐ubiquitinated SAR1A in L929^USP1‐Flag^ cells. E) Co‐IP/WB assay was used to detect the time‐effect of interaction between STING and oligo‐ubiquitinated SAR1A in L929 ^HA‐UB‐K27^ cells. F) Co‐IP/WB analysis was used to detect the interaction between STING and SEC24C or SEC23A in L929 cells. All data were obtained from biological replicates conducted more than 3 times. D,E) Quantifications of grayscale values (right), data were represented as mean ± S.D. (*n* = 3). Statistical significance was determined by one‐way ANOVA (N.S., no significance; *, *P* < 0.05; **, *P* < 0.01; ****, *P* < 0.0001). G) The mechanistic model diagram illustrating USP1 primarily removes K27‐linked oligo‐ubiquitination from SAR1A.

To elucidate the fundamental mechanism by which USP1 inhibits STING transport, leveraging the substrate specificity of DUB enzymes, we initially sought to determine whether USP1 directly interacts with STING to mediate its function. Intriguingly, USP1 did not engage with STING during its activation phase (**Figure** **5**A). Based on this insight, our subsequent strategy employed ubiquitination substrate screening for USP1, coupled with tracking alterations in the STING transport complex, following USP1 inhibition by ML323. Upon analyzing the entire transport axis, we observed that ML323 does not influence the binding affinity between STEEP and STING (Figure 5B). Furthermore, during the STEEP‐mediated recruitment of functional factors, we did not observe any binding between STING and vacuolar protein sorting 34 (VPS34) under the same conditions (Figure 5B). We did not observe any changes in the ubiquitination status of VPS34 or STEEP following the addition of ML323, indicating that the substrate targeted by USP1 is unlikely to be either of these two factors. Remarkedly, following the formation of ER curvature, which precedes COP‐II vesicle formation, we identified a significant discovery in the group treated with both ML323 and S‐cddA: the interaction between STING and SAR1A was not detected in the unmodified form of SAR1A, several higher molecular weight SAR1A bands emerged, either newly appearing or significantly increasing in intensity (Figure 5B); however, the binding of SEC24C and SEC23A within the COP‐II complex to STING occurs at their native protein positions. This interaction is observed exclusively when ML323 is combined with S‐cddA (Figure 5F). This suggests that the higher molecular weight forms are likely ubiquitinated SAR1A. Notably, among the proteins interacting with STING, factors, such as STEEP and SEC24C do not exhibit molecular weight‐increased forms. Consequently, we postulate that ML323 inhibits USP1 by promoting the accumulation of SAR1A ubiquitination, rather than that of SEC24C or SEC23A. To test this hypothesis, we overexpressed ubiquitin variants containing 7 types of K mutations (retaining only one K) in cells to ascertain whether SAR1A undergoes a specific type of ubiquitination. Results demonstrated that combined treatment with S‐cddA and ML323, only the K27‐ubiquitinated form of SAR1A showed a marked increase, with a molecular weight of ≈70 kDa, a phenomenon absent in other K‐mutated systems (Figure 5C; and Figure S16, Supporting Information). However, neither the addition of ML323 nor its combination with S‐cddA significantly altered the ubiquitination status of SEC24C or SEC23A (Figure S17, Supporting Information). This suggests that ML323 regulates the oligoubiquitination of SAR1A at the K27 linkage, a key mechanism in facilitating STING loading onto COP‐II. We subsequently conducted further validation to confirm this finding.

This ubiquitination modification is termed oligoubiquitination, to ascertain whether this modification is directly governed by USP1's deubiquitination activity, further validation is necessary. Co‐IP/WB results reveal that the modified form of SAR1A at this site, which interacts with USP1, is relatively easy to capture. Under these conditions, the observed temporal dynamics demonstrate that the interaction between USP1 and SAR1A K27‐oligoubiquitination initially increases and then decreases, consistent with the deubiquitination process mediated by USP1. However, when USP1's deubiquitination activity is inhibited, the oligo‐ubiquitinated state of SAR1A persists for a certain duration (Figure 5D). This underscores that USP1‐mediated deubiquitination is the primary regulatory mechanism affecting the presence or level of SAR1A K27‐oligoubiquitination. To further investigate whether this oligoubiquitinated state is directly linked to STING transport activity, we conducted a time‐course analysis. The results showed that SAR1A K27‐oligoubiquitination, bound to STING, was significantly detected in the group treated with the combination of ML323 and S‐cddA, the rate of increase observed between 1 and 2 h is remarkably pronounced, but not in groups treated with ML323 or S‐cddA alone (Figure 5E). To further validate these findings, we retained modifications at other lysine sites and specifically mutated lysine 27 (K27) to arginine (R) of ubiquitin to inactivate this site. This mutant was then incorporated into the evaluation system. The results demonstrated that overexpression of the HA‐Ubiquitin (K27R) mutant in cells abolished the detection of the K27‐linked oligoubiquitination state of SAR1A under cotreatment with ML323 and S‐cddA (Figure S18, Supporting Information). Additionally, to validate the specificity of targeting USP1 as a DUB in this mechanism, we tested various DUB inhibitors under the same conditions as ML323 and assessed their effects on the K27‐linked oligoubiquitination of SAR1A. The results demonstrated that other DUB inhibitors, when combined with S‐cddA, failed to replicate the K27‐linked oligoubiquitination state of SAR1A observed with ML323 (Figure S19, Supporting Information). This finding underscores the unique specificity of USP1 in the mechanism of action. Collectively, these findings indicate two key points: 1) SAR1A associated with STING is in the form of K27 oligo‐ubiquitination; 2) under normal USP1 activity, even robust stimulation with a high concentration of STING activator negatively regulates the interaction between STING and Ubn‐SAR1A (K27), due to USP1's oversight and execution of deubiquitination of this oligo‐ubiquitinated form. Moreover, interaction analysis between STING and SEC24C or SEC23A further confirmed that USP1 inhibition significantly enhances the binding of STING to SEC24C or SEC23A, thereby demonstrating that suppressing USP1 can accelerate the formation of the STING transport complex (Figure 5F). In conclusion, the K27 oligo‐ubiquitinated form of SAR1A may function as a crucial active factor facilitating the formation of the STING transport complex, while USP1 serves as a pivotal negative regulatory monitor, promptly deactivating this active state to impede the formation of the transport complex (Figure 5G). The impairment of this negative regulatory function markedly enhances the formation of the STING transport complex over a certain period.

### 2.5 Blockade of USP1 Enhances Radiation‐Induced Immunity and Promotes the Conversion of Tumors from Immunologically “Cold” to “Hot”

**Figure 6:**
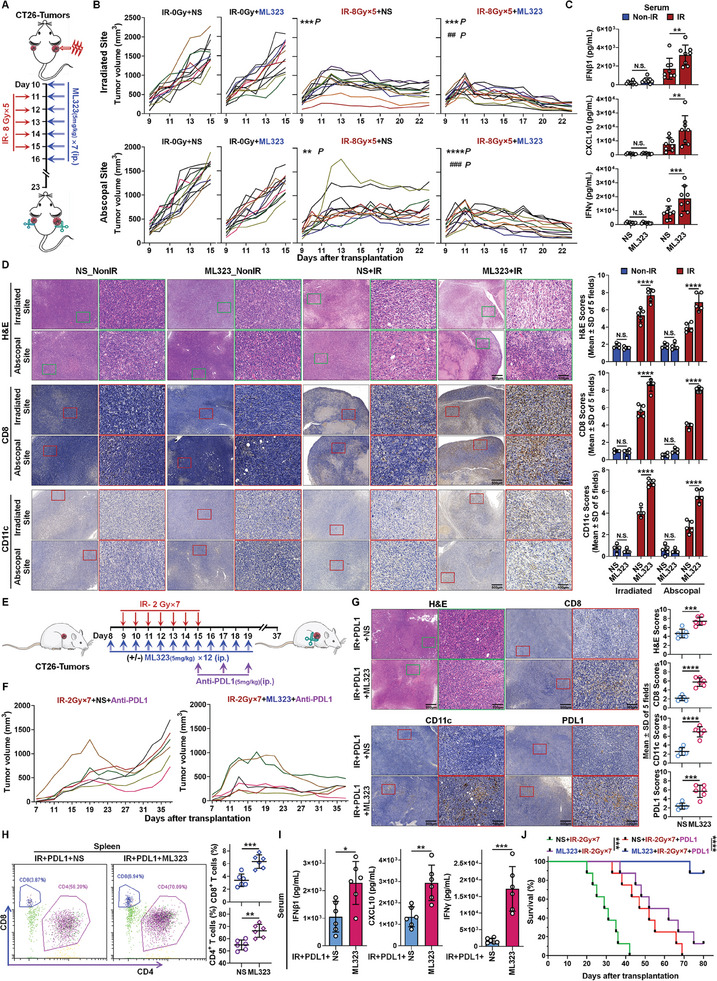
Effects of ML323 enhance radiation‐induced antitumor immune responses. A) Schematic diagram of the experimental design demonstrating the abscopal effect of RT, involving tumor‐bearing mice (CT26 tumors implanted in bilateral axillae) treated with IR and ML323. B) Tumor growth curves for irradiated (IR) and nonirradiated (NonIR) mice administered either ML323 or vehicle (NS, Normal Saline), categorized into the 4 groups (*n* = 12/group) listed in the figure. C) The serum levels of IFNβ1, IFN‐γ, and CXCL10 were detected from 4 groups. D) Analysis of H&E and IHC‐CD8 and CD11c staining of mouse tumor sections. Left: representative images; Right: statistics of HE and IHC scores (*n* = 5/group). E) Schematic diagram of the experimental design demonstrating the effect of RT + immunotherapy, involving mice bearing CT26 tumors implanted in the right axilla, wherein the groups receive irradiation, along with administration of ML323 and PDL1 monoclonal antibody. F) Tumor growth curves of irradiated (IR) mice treated with PDL1 and ML323 or vehicle (N/S), showing tumor control in both the RT + Anti‐PDL1 and RT + Anti‐PDL1 + ML323 treatment groups (*n* = 6/group). G) Analysis of H&E and IHC‐CD8, CD11c, and PDL1 staining of mouse tumor sections. Left: representative images; Right: statistics of HE and IHC scores (*n* = 6/group). H) Mouse spleen lymphocytes were extracted for flow cytometry analysis and showed percentage of infiltrating CD4^+^ T cells and CD8^+^ T cells. Left: representative results; Right: statistics for flow cytometry. I) The serum levels of IFNβ1, IFN‐γ, and CXCL10 in mice were detected. J) Kaplan–Meier survival curves of treated mice demonstrated survival outcomes in the IR + NS group; IR + ML323 group; IR + PDL1 + NS group, and IR + PDL1 + ML323 group. Data were represented as mean ± S.D. (*n* ≥ 5). Statistical significance was determined by two‐way ANOVA (N.S., no significance; *, *P* < 0.05; **, *P* < 0.01; ***, *P* < 0.001; ****, *P* < 0.0001).

It has been unequivocally demonstrated that USP1 mediates the production of stress resistance factors by innate immune cells. Through a series of in vitro experiments, we have shown that targeting and inhibiting USP1 enzymatic activity with ML323 can effectively surmount this obstacle. However, the potential of ML323 to exert synergistic effects with radiation therapy in vivo remains uncertain, necessitating further comprehensive evaluation of its in vivo efficacy. Initially, it is imperative to determine whether ML323 possesses the capability to potentiate radiation‐induced immune responses. The observation of the abscopal effect of radiation is widely recognized as the most direct method for assessing radiation‐induced immune responses.^[^ ^21^ ^]^ In accordance with previously published studies, we designed our animal experiments by administering a moderately high dose of continuous irradiation to one side of tumor‐bearing animals for 5 consecutive days, subsequently monitoring the growth of contralateral tumors to discern the trend of the effect (**Figure** **6**A). Moreover, regarding the choice of drug intervention, preliminary cellular‐level experiments indicated that the synergistic effect of ML323 followed by STING agonist activation remains intact (Figure S20A, Supporting Information). Consequently, ML323 was administered via intraperitoneal injection (ip.) prior to RT. The results demonstrated that this radiation strategy effectively controlled the tumor volume in both the irradiated and abscopal tumors. Notably, ML323, which is ineffective as a monotherapy, significantly enhanced the control of tumor volume in both the irradiated and contralateral tumors by radiation, particularly in preventing the recurrence of contralateral tumors post‐treatment; the synergistic effect in treatment was strikingly apparent (Figure 6B; and Figure S20B,C, Supporting Information). In terms of immune response evaluation, we observed a substantial increase in the levels of IFNβ1 and CXCL10 in the serum of mice in the ML323 combined with radiation therapy group compared to those in the radiation therapy alone group (Figure 6C). Furthermore, there was a marked increase in the infiltration of CD11c^+^ DC cells, especially within the abscopal tumors (Figure 6D). These findings are consistent with our previously elucidated mechanism supporting STING activation. Subsequently, in the assessment of adaptive immunity, we also observed a significant elevation in IFN‐γ levels in the serum, further indicating the potential of ML323 to augment tumor immune responses following RT (Figure 6C). To further corroborate these findings, we evaluated the infiltration of cytotoxic lymphocytes within the tumors. The results revealed a pronounced increase in the infiltration of CD4^+^ and CD8^+^ T lymphocytes within the irradiated tumors in the presence of ML323, with an even more significant increase in the abscopal tumors (Figure 6D; and Figure S21A, Supporting Information). Granzyme B and perforin, key indicators of cytotoxic CD8^+^ T cell activity, have been analyzed and found to exhibit a positive correlation with CD8^+^ T cell infiltration. Notably, ML323 significantly upregulates the levels of these indicators within tumors following RT, regardless of whether the tumor resides on the irradiated side or the distant side (Figure S21B,C, Supporting Information). Additionally, HE staining results also demonstrated a higher proportion of inflammatory cell infiltration and cytotoxicity in tumors treated with the combination of ML323 and RT (Figure 6D). To address this, we employed nude mice to further evaluate the abscopal effects of RT (Figure S22A, Supporting Information). Our results demonstrated that while radiotherapy exhibited a strong therapeutic effect on tumors at the irradiated site, its efficacy in controlling tumors at distant sites was relatively limited. Notably, the trends in tumor volume changes, both at the irradiated and distant sites, remained unaffected by the continued administration of ML323 (Figure S22B,C, Supporting Information). These findings suggest that ML323 primarily enhances the local and systemic immune response following RT, rather than functioning as a radiosensitizer or exerting any other synergistic effects. In conclusion, the capacity of ML323 to enhance the abscopal effect of radiation therapy is profoundly evident.

We have observed the limited efficacy of immunotherapy in addressing “cold” immune phenotype tumors, such as colorectal cancer (CRC).^[^ ^22^ ^]^ Harnessing the potential of radiotherapy (RT) to induce a “cold‐to‐hot” tumor conversion may facilitate the integration of immunotherapy and expand the role of RT in comprehensive CRC treatment. Notably, PDL1, encoded by the CD274 gene, is a major driver of tumor immune escape, and its changes in expression have garnered significant attention, particularly during the “cold‐to‐hot” transformation of tumor immune phenotypes, where its expression levels increase. This process is mediated by the transcriptional activation effects of type I interferons, as PDL1 (CD274) is well‐documented as a type I IFN‐inducible gene.^[^ ^23^ ^]^ We also recognize that the general ineffectiveness of PDL1 antibodies in treating colorectal cancer patients stems from the low expression levels of PDL1, which lack the necessary therapeutic target characteristics.^[^ ^24^ ^]^ We hypothesize that the robust type I IFN response induced by ML323 following radiotherapy could enhance PDL1 expression. This upregulation of PDL1 may render it a viable therapeutic target, thereby improving the effectiveness of PDL1 monoclonal antibodies. To validate this hypothesis, we first utilized the tumor cluster in vitro culture model shown in Figure 1C. Characterization of PDL1 expression across different groups revealed that the combination of ML323 and RT drastically increased PDL1 expression levels (Figure S23A, Supporting Information). Next, we employed a clinically prevalent long‐course, large‐fraction RT regimen, and introduced anti‐PDL1 monoclonal antibodies toward the conclusion of the RT cycle as an adjunctive treatment to evaluate the impact of ML323 (Figure 6E). It is important to note that anti‐PDL1 monoclonal antibodies have been shown to be ineffective or only marginally effective in CRC during clinical trials, and thus their standalone use has not been incorporated into clinical guidelines.^[^ ^25^ ^]^ The results indicated that, although the combination of RT and anti‐PDL1 could temporarily control tumor growth, tumors typically recurred within 1–2 weeks following the cessation of treatment. Encouragingly, the addition of ML323 completely reversed the adverse recurrence of tumors and led to a sustained reduction in tumor volume (Figure 6F; and Figure S23B, Supporting Information), which we hypothesize is closely linked to the immune enhancement mediated by ML323. Consequently, upon evaluating the immune infiltration within the tumors, we observed a significant increase in the infiltration of CD11c^+^ DC cells; naturally, this was accompanied by an increased infiltration of CD8^+^ T lymphocytes, and the overall proportion and cytotoxicity of inflammatory cells within the tumor were correspondingly elevated (Figure 6G), the levels of granzyme B and perforin exhibit a trend that is essentially consistent with the trend of CD8^+^ T cell infiltration (Figure S23C, Supporting Information). Notably, PDL1 expression levels were also significantly elevated under the influence of ML323 (Figure 6G). It is widely believed that low PDL1 expression levels in CRC are a critical factor in the limited efficacy of anti‐PDL1 therapy. The introduction of ML323, while facilitating the “cold‐to‐hot” conversion of tumors, also activated the expression of immune checkpoint inhibitors such as PDL1. This activation may provide a critical opportunity for the deployment of immune checkpoint inhibitors, potentially contributing to the sustained immune activation against tumors and the remarkable antitumor efficacy observed. In evaluating systemic immune status, we also found that ML323 significantly enhances the production of CD4^+^ and CD8^+^ T lymphocytes in the spleen (Figure 6H). By assessing the levels of Type I IFN‐mediated cytokines and Type II IFN indicative of adaptive immune activation in mouse serum (Figure 6I), it can be inferred that ML323 substantially reconfigured the immune landscape in vivo. Moreover, based on the evaluation of these immune parameters, we assessed the overall survival outcomes under this treatment regimen. The results demonstrated that, compared to RT alone or in combination with anti‐PDL1, the triple combination of ML323, RT, and anti‐PDL1 antibody significantly improved the survival rate of mice over an 80‐day period (Figure 6J). We also observed that, even in the absence of anti‐PDL1 monoclonal antibodies, ML323 could enhance the efficacy of RT alone. However, the addition of anti‐PDL1 can achieve prolonged survival, which has profound implications for advancing therapeutic efficacy (Figure 6J), further corroborating the robust capacity of ML323 to promote the “cold‐to‐hot” conversion of tumors following RT.

### 2.6 Elevated USP1 Levels in Nontumor Cells within TME Prior to RT Imply the Failure of RT‐Induced Immunity and are Indicative of an Unfavorable Prognosis in LARC

Emerging evidence indicates that RT not only mediates its antitumor effects through direct DNA damage to tumor cells but also actively remodels the tumor immune microenvironment, a pivotal mechanism underpinning its therapeutic efficacy. The immune response triggered by RT is recognized as a key determinant of its effectiveness and the longevity of its therapeutic outcomes.^[^ ^3a^ ^]^ Beyond the dominance of tumor cells, the TME comprises diverse stromal cells, including tumor‐associated fibroblasts, mesenchymal stem cells, pericytes, antigen‐presenting cells like DCs, and tumor‐associated macrophages, each fulfilling distinct functions around the tumor cells. Our focus is on the inherent potential of these cells as innate immune entities capable of detecting and responding to signals released by irradiated tumor cells, leading to the secretion of type I IFNs that stimulate the adaptive immune response, effectively “reviving” antitumor immunity within the TME. Consequently, we are dedicated to developing targeted interventions that amplify the sensing capabilities of innate immune cells to robustly reshape the antitumor immune phenotype of tumors following RT. Our prior research has compellingly demonstrated, both in vitro and in vivo, that targeting USP1 can synergistically enhance innate immune signaling within tumors post‐RT. Our next objective is to assess the clinical feasibility of this strategy through extensive clinical sample analyses of locally advanced rectal cancer (LARC). The rationale for selecting LARC as the research focus is that neoadjuvant RT is an essential treatment modality for this cancer type, enabling the collection of specimens both pre‐ and post‐treatment. We will investigate the potential clinical implications of USP1 by evaluating its expression in nontumor cells within clinical specimens, a critical step given our cellular‐level findings that higher USP1 expression correlates with enhanced STING pathway inhibition, complicating targeted therapeutic intervention (Figure S24, Supporting Information).

**Figure 7:**
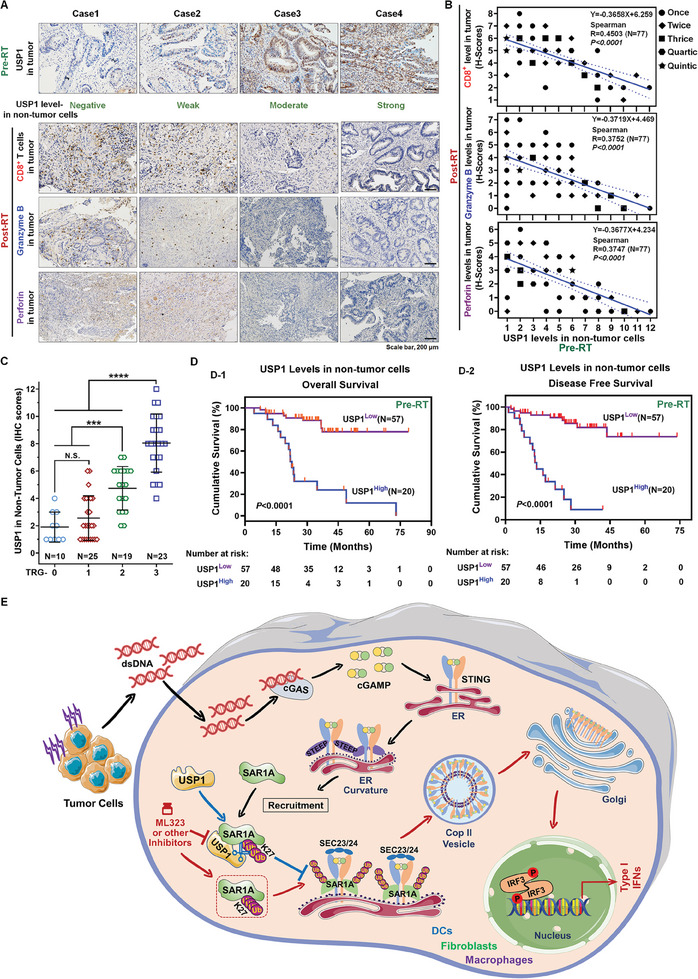
Prognostic implications of USP1 expression in non‐tumor cells and its correlation with immunological response and survival outcomes in LARC. A) IHC staining for USP1 in non‐tumor cells pre‐RT and for CD8^+^ T cells infiltration, as well as the levels of Granzyme B and Perforin in LARC tumors post‐RT in 4 representative cases. B) Spearman correlation analysis showing a significant inverse correlation between USP1 levels in nontumor cells pre‐RT and CD8^+^ T cell infiltration, as well as the levels of Granzyme B and Perforin post‐RT in tumor tissues. C) Box plot depicting USP1 IHC scores in nontumor cells according to TRG after RT (one‐way ANOVA). D) The Kaplan–Meier survival curve showed OS D‐1) and DFS D‐2) based on USP1 levels in nontumor cells. (N.S., no significance; *, *P* < 0.05; **, *P* < 0.01; ***, *P* < 0.001; ****, *P* < 0.0001). E) Schematic model. Our research has uncovered that SAR1A must be subjected to K27‐linked oligo‐ubiquitination to mediate the formation of the STING‐COP‐II transport complex. However, USP1's deubiquitinating function can swiftly intercept and deactivate SAR1A, resulting in a cessation of STING transport and a consequent weakening of antitumor immune activation. Inhibiting the catalytic activity of USP1 maintains SAR1A activation, thereby significantly promoting STING trafficking and subsequent activation, this enhancement considerably facilitates the innate immune response within the TME post‐RT.

In tumor tissues from 77 LARC patients at The First Affiliated Hospital, Sun Yat‐sen University, a distinct inverse correlation was observed between USP1 expression in nontumor cells before RT and the infiltration of cytotoxic CD8^+^ T lymphocytes after treatment (**Figure** **7**A). This significant negative correlation suggests that USP1 expression levels in nontumor cells within the TME prior to RT can accurately predict the degree of adaptive immune activation induced by the treatment (Figure 7B). Meanwhile, we evaluated the correlation between the expression levels of Granzyme B and Perforin in human tumor specimens post‐RT and their levels in both tumor and nontumor cells pre‐RT. This analysis revealed the expression level of USP1 in pre‐RT nontumor cells is significantly negatively correlated with CD8^+^ T cell infiltration and cytotoxic activity following RT (Figure 7A,B). To assess RT efficacy, we utilized the tumor regression grade (TRG), where a lower degree of regression indicates a higher TRG score. Analysis showed a positive correlation between USP1 levels and TRG: higher TRG scores corresponded with elevated USP1 expression (Figure 7C). Stratification by TRG revealed that the negative correlation between pre‐RT USP1 levels in nontumor cells and post‐RT CD8^+^ lymphocyte infiltration, as well as the levels of Granzyme B and Perforin in tumors, was specifically observed in the TRG‐3 group. In contrast, no significant correlation was found in the TRG‐0/1 or TRG‐2 groups (Figure S25A–C, Supporting Information). This indicates that the overall significant negative correlation is largely attributable to the TRG‐3 subgroup. Further survival analysis demonstrated that elevated USP1 levels were significantly associated with poorer prognosis (Table S1, Supporting Information), specifically correlating with reduced overall survival (OS) and disease‐free survival (DFS) before RT (Figure 7D). To further distinguish the indicative capacity of USP1 levels in tumor cells versus nontumor cells and to consolidate the aforementioned conclusions, we continued to characterize USP1 levels specifically within tumor cells in clinical tissue specimens. The results demonstrated that USP1 levels in tumor cells showed no significant correlation with the degree of CD8^+^ T cell infiltration, granzyme B, or perforin (Figure S26A, Supporting Information) levels; No significant association with TRG (Figure S26B, Supporting Information); No significant relationship with survival outcomes (Table S2, Supporting Information), including DFS or OS (Figure S26C, Supporting Information). These findings suggest that USP1 levels in tumor cells may be less important than USP1 levels in nontumor cells in indicating immune status, survival, and prognosis. Overall, the elevated expression levels of USP1 in nontumor cells within the TME prior to RT serve as a potent indicator of the suboptimal or ineffective immune activation induced by RT. This underscores the compelling scientific rationale for targeting USP1, suggesting that pharmacological modulation of USP1 could potentially reverse the impaired activation of anti‐tumor immunity post‐RT. These findings strongly support further drug development and the initiation of new clinical trials. Moreover, our results imply that the RT‐induced immune activation within tumors may predict the durability of the treatment's efficacy in patients. Therefore, implementing targeted interventions to enhance anti‐tumor immune responses post‐RT holds substantial clinical significance.

## 3 Discussion

Innate immunity is a fundamental defense mechanism that organisms utilize in response to external pathogenic invasion or internal stress.^[^ ^26^ ^]^ Within the TME, the innate immune response is integral to various antitumor immune processes, including tumor surveillance, antigen presentation, the initiation and sustainment of T cell activity, and the establishment of immune memory.^[^ ^27^ ^]^ Central to these processes is the cGAS‐STING pathway, which plays a critical role in detecting intracellular dsDNA signals and initiating type I IFNs‐dependent innate immune responses. The cytosolic accumulation of dsDNA is detected and captured by the receptor cGAS, which subsequently synthesizes the natural ligand cGAMP for the STING. cGAMP then induces STING aggregation, prompting its transport from the ER to the Golgi apparatus. Upon Golgi localization, aggregated STING recruits the key activator TBK1, which dimerizes and mutually activates, leading to the phosphorylation of STING. Phosphorylated STING then recruits IRF3, which is likewise phosphorylated by TBK1. The phosphorylated IRF3 dimerizes and translocate to the nucleus, where it acts as a transcriptional activator, inducing the production of IFNβ. IFNβ serves as a crucial mediator bridging innate and adaptive immunity, orchestrating the composition and function of innate and adaptive immune cells within the TME.^[^ ^28^ ^]^ It initiates and maintains the tumor's immunoinflammatory phenotype by inducing the expression of various cytokines and chemokines, facilitating immune cell infiltration, activating NK cells for direct tumor lysis, promoting DC maturation, and CD8^+^ T cell activation, and fostering the development of antitumor immune memory.^[^ ^29^ ^]^

Building on the mechanism by which the dsDNA‐cGAS‐STING‐mediated type I IFNs‐innate immune response promotes the conversion of tumors from “cold” to “hot,” current research is centered on the utilization of direct STING agonists to enhance innate immunity. Consequently, the development of STING activators has attracted significant interest, with the primary strategy focusing on the optimization and modification of cGAMP's structural properties. Various classes of small molecules, including both nucleoside and non‐nucleoside analogs, have been developed and are presently in preclinical or Phase I clinical trials, although none have yet received market approval.^[^ ^30^ ^]^ Notably, direct STING agonists induce the most pronounced responses in susceptible innate immune cells. However, due to the widespread distribution of these cells throughout the body, the use of direct STING agonists could precipitate severe systemic immune disorders. Consequently, most in vivo studies on STING agonists have had to employ intertumoral injection or targeted delivery systems, which significantly hinders their clinical application.^[^ ^31^ ^]^ In this context, we conclude that RT, as a critical localized treatment modality, offers distinct advantages in the activation of cGAS‐STING. It enables precise targeting of the tumor region while mitigating systemic side effects, thereby obviating the need for STING agonists that could provoke systemic adverse reactions. Theoretically, the cytosolic DNA response elicited by RT can activate the cGAS‐STING signaling pathway, thereby achieving “immune awakening.” However, according to literature reports and preliminary research findings from the applicant's team, it has been observed that radiation‐induced release of tumor cell genomic DNA or mitochondrial DNA fragments demonstrates a limited ability to activate the type I interferon pathway via dsDNA in DCs or other APCs within the TME, as well as in tumor cells through the cGAS‐STING axis.^[^ ^21^, ^32^ ^]^ This limited activation presents challenges in triggering downstream innate immune responses, thereby explaining the difficulty in observing systemic immune activation or the abscopal effect of RT in clinical settings.^[^ ^6^, ^33^ ^]^ To reliably induce an immune response through RT, it is crucial to specifically address the inhibitory factors associated with the dsDNA‐cGAS‐STING signaling pathway, making the elucidation of the underlying mechanisms essential.

Ubiquitin is a highly conserved peptide chain comprising seven lysine (K) residues (K6, K11, K27, K29, K33, K48, and K63). Facilitated by activating enzyme E1 and conjugating enzyme E2, ubiquitin molecules polymerize at distinct K sites to generate various polyubiquitin chain types. These chains are subsequently attached to target proteins by ligase E3, resulting in ubiquitinated proteins (Ub‐Protein). The distinct linkages within polyubiquitin chains create unique protein scaffolds, capable of recruiting specific functional molecules, thereby mediating diverse downstream activities of the target proteins.^[^ ^34^ ^]^ DUBs are pivotal molecular targets, modulating protein degradation and function, and consequently influencing tumorigenesis, progression, and resistance. Recent studies have increasingly highlighted DUBs’ critical role in maintaining the homeostasis and function of key immune checkpoints and regulatory factors in immune cells, thus earning the designation of “secondary immune checkpoints.”^[^ ^9a–c^ ^]^ In the context of STING ubiquitination, E3 ubiquitin‐protein ligase RNF5 is reported to mediate K48‐linked ubiquitination of STING, promoting its degradation,^[^ ^35^ ^]^ while E3 ubiquitin‐protein ligase RNF26 mediates K11‐linked ubiquitination, competitively counteracting this process.^[^ ^36^ ^]^ Moreover, K63‐ and K27‐ linked ubiquitination of STING enhances the recruitment of TBK1 and IRF3.^[^ ^37^ ^]^ Additionally, there are reports suggesting that K63‐linked ubiquitination of TBK1 and IRF3 is essential for their activation, whereas K48‐linked ubiquitination drives them toward inactivation and degradation.^[^ ^38^ ^]^ While the regulatory role of ubiquitination in cGAS‐STING activation has attracted growing attention, research on the impact of deubiquitination on this pathway remains relatively scarce.

USP1 is a member of the ubiquitin‐specific protease family, whose catalytic activity is contingent upon activation through the auxiliary protein WD repeat‐containing protein 48 (UAF1, also known as WDR48). Recent studies have progressively elucidated the immunoregulatory roles of the USP1‐UAF1 complex.^[^ ^39^ ^]^ For instance, one study indicates that USP1‐UAF1 modulates the differentiation of T helper cell 17 and Treg cells by stabilizing Tafazzin,^[^ ^40^ ^]^ while another demonstrates that USP1‐UAF1 stabilizes NLR family pyrin domain containing 3 (NLRP3), thereby activating the NLRP3‐mediated inflammatory phenotype.^[^ ^41^ ^]^ In the context of type I interferon pathway regulation during RNA virus infection, a report suggests that USP1‐UAF1 augments antiviral responses by stabilizing TBK1.^[^ ^42^ ^]^ However, the regulatory mechanism of USP1‐UAF1 within the STING‐TBK1‐IRF3 signaling pathway remains uncharted. In our study, for the first time, we have elucidated that upon receiving the cGAMP signal after RT, STING undertakes a sequence of preparatory actions on the ER membrane before its loading onto the COP‐II transport vesicle. During this process, USP1 intervenes through deubiquitination, consequently deactivating SAR1A (in its K27 type oligo‐ubiquitinated condition), thereby causing a delay in the transport of STING. As a result, STING remains predominantly sequestered in the ER for a certain duration poststress, thereby disrupting the activation of the STING signaling pathway.

SAR1A, a small GTPase that oscillates between an active GTP‐bound state and an inactive GDP‐bound state, primarily functions in vesicle‐mediated transport from the ER to the Golgi apparatus. When in its active GTP‐bound form, SAR1A integrates into the ER membrane, where it recruits the remaining components of the COP‐II complex. This assembly and polymerization of COP‐II on the ER membrane are crucial for cargo sorting and the deformation and budding of vesicles destined for the Golgi.^[^ ^43^ ^]^ In contrast, SAR1B, as another GTPase, functions entirely oppositely to SAR1A. It specifically interacts with the cargo receptor surfeit locus protein 4 to facilitate the transport of lipid‐laden lipoproteins, including apolipoprotein B and apolipoprotein A1. This process mediates the retrograde transport of proteins from the Golgi to the ER.^[^ ^44^ ^]^ Recent studies are increasingly focusing on the roles of SAR1‐type GTPases on the ER membrane, although analyses of their inherent activity and regulatory mechanisms remain limited.^[^ ^45^ ^]^ Certain reports suggest that SAR1‐GTP induces positive membrane curvature 10–20 times greater than SAR1‐GDP; furthermore, dimerization of the GTP‐bound form of SAR1 intensifies curvature generation. This line of research offers a novel perspective on SAR1 functionality.^[^ ^45a^ ^]^ However, we observe that in studies involving GTP‐GDP interconversion regulation, the binding conformations of protein‐GTP often fundamentally differ from those of protein‐GDP. Thus, we postulate that SAR1‐type proteins have a regulatory system governing their activity and state, intricately linked to the innovative mechanism presented in our study. The K27 and K63‐linked oligoubiquitination forms generally possess activating properties for protein substrates.^[^ ^46^ ^]^ Notably, in the presence of USP1 activity, even a potent activation signal delays the emergence of the Ubn‐SAR1A (K27) active form, while a weak activation signal entirely prevents the active form's appearance. This results in a prominent ER retention effect of STING. This suggests that the dynamic interplay between deubiquitination and ubiquitination endures throughout the activation process. Conversely, when USP1 activity is inhibited, the balance markedly shifts toward the activated state. In conclusion, our research enhances the understanding of the pivotal role of ubiquitination‐deubiquitination regulation in STING pathway activation. We also acknowledge the critical importance of investigating the ubiquitination regulation of SAR1A, such as through its E3 ligase, which will be a focal point of our subsequent research endeavors.

## 4 Conclusion

Through the screening of DUB inhibitors, we have identified that the USP1 inhibitors significantly enhance the dsDNA response within the TME post‐RT. Subsequent mechanistic investigations have revealed that, within the TME, cells predisposed to innate immune responses—such as DCs, fibroblasts, and macrophages—rapidly initiate the synthesis of cGAS upon receiving dsDNA released from irradiated tumor cells. This process sufficiently reserves cGAMP for subsequent STING activation. Upon cGAMP transmitting the activation signal to STING, STING promptly assembles on the ER membrane in preparation for transport to the Golgi apparatus. Our research has uncovered that SAR1A must be subjected to K27‐linked oligo‐ubiquitination to mediate the formation of the STING‐COP‐II transport complex. However, USP1's deubiquitinating function can swiftly intercept and deactivate SAR1A, resulting in a cessation of STING transport and a consequent weakening of antitumor immune activation. Inhibiting the catalytic activity of USP1 maintains SAR1A activation, thereby significantly promoting STING trafficking and subsequent activation, this enhancement considerably facilitates the innate immune response within the TME post‐RT, providing a compelling impetus for the transition of tumors from a “cold” to “hot” state following RT (Figure 7E).

## 5 Experimental Section

### Ethical Approval and Consent to Participate

Animal experiments were approved by the Animal Welfare and Ethics Committee of Jinan University. All experiments were conducted in the central laboratory of the Overseas Chinese Hospital affiliated to Jinan University. The ethical Approval Number is 20240227‐0076.

The clinical analysis was approved by the Institutional Ethics Committee for Clinical Research and Animal Trials of the First Affiliated Hospital, Sun Yat‐sen University (SYSUFAH) (Approval No. [2023]‐079). A total of 77 formalin‐fixed and paraffin‐embedded tumor biopsy specimens were collected from patients pathologically diagnosed with LARC prior to RT from SYSUFAH. They all received radical RT (VMAT, 50 Gy, 2 Gy/day, 5 days/week). Informed consent was obtained from the patients, and the study is compliant with all relevant ethical regulations regarding research involving human participants.

Following RT, TRG grading was determined according to the 4‐tier classification outlined in the 8th edition of the AJCC guidelines, with the following definitions: TRG 0 signifies the absence of viable cancer cells, indicating a complete response; TRG 1 denotes the presence of single cells or occasional small clusters of cancer cells, suggesting a near‐complete response; TRG 2 refers to residual cancer with evident tumor regression, where more than just single cells or small clusters are present, representing a partial response; and TRG 3 indicates extensive residual cancer without any visible tumor regression, denoting a poor or no response.

### Cell Lines and Cell Culture

RAW264. 7, L929, L929^USP1‐Flag^, L929^mCherry‐Sting^, HEK293T, L929^HA‐UB‐WT^, L929^HA‐UB‐K6^, L929^HA‐UB‐K11^, L929^HA‐UB‐K27^, L929^HA‐UB‐K29^, L929^HA‐UB‐K33^, L929^HA‐UB‐K48^, L929^HA‐UB‐K63^, L929^HA‐UB‐K27R^ cells were cultured in Corning's DMEM 1 × medium supplemented with 10% fetal bovine serum, 100 units mL^−1^ penicillin, and 100 µg mL^−1^ streptomycin. BMDCs, DC2.4, H22, CT26 cells were cultured in Corning's RPMI‐1640 1 × medium supplemented with 10% FBS, 100 units mL^−1^ penicillin, and 100 µg mL^−1^ streptomycin. All cells were cultured at 37 °C in a humidified atmosphere with 5% CO_2_.

### Lentiviral Transduction

The coding sequences of full‐length mouse USP1 tagged with 3 × Flag at the C‐terminus, full‐length mouse STING tagged with mCherry at the N‐terminus, and ubiquitin (wild type, K6/K11/K27/K29/K33/K48/K63/K27R‐specificity) tagged with HA at the N‐terminus were inserted into the pCDH‐EF1‐MCS‐T2A‐Puro plasmid respectively. Lentiviruses are generated by cotransfecting the above plasmid with psPAX2 and pMD2. G plasmids into HEK293T cells, then the virus is transduced into L929 cells. The stable cell lines are then produced by selecting with puromycin as described previously.^[^ ^47^ ^]^

### In Vitro Cell Coculture

After radiation treatment (radiation dose 40 Gy), resuspended the H22 (cell number 1 × 10^7^) cells in RPMI‐1640 culture medium containing 5 µm DUB inhibitors (the inhibitors are shown in Figure S1, Supporting Information), and add them to the adherent cultured BMDCs/DC2. 4/L929/RAW264.7 (cell number 5.0 × 10^6^) which have been pre‐incubated with 5 µm DUB inhibitors, cocultured for 12 h. Following coculture with H22 cells, PBS was used to oscillatory washed the BMDCs/DC2. 4/L929/RAW264.7 cells three times, and observed them under a microscope to ensure that there were no adherent H22 cells, then extracted the RNA of BMDCs/DC2.4/L929/RAW264.7 cells, reverse transcribed to cDNA, and perform RT‐qPCR to detect the expression of *Ifn‐β1* and *Cxcl10*.

### Radiation Therapy for Tumor Cells

1 × 10⁷ H22/CT26 cells were irradiated with single doses of 4, 6, 8, 10, 20, and 40 Gy, or with continuous irradiation (4, 8, 16, and 32 Gy) for 3 days. Then cultivate them using RPMI‐1640 culture medium with or not with 5 µm USP1 inhibitor ML323 for 12 h. Afterward, the H22/CT26 cells were collected to extract RNA, reverse‐transcribed the RNA into cDNA, and then use RT‐qPCR to detect the expression levels of *Ifnβ1* and *Cxcl10*.

### Exosome Extraction

After IR treatment (radiation dose 40Gy), CT26/H22 cells continued to culture for 12 h. Then the cell culture medium was collected and subjected to continuous centrifugation at 4 °C: first at 300 g for 10 min to remove floating and dead cells, followed by 2000 g for 30 min. Next, the supernatant was further centrifuged at 4 °C at 10 000 g for 30 min to eliminate subcellular components, such as cell debris. A subsequent centrifugation at 10 000 g for 70 min at 4 °C resulted in a pellet containing exosomes. The pellet was then dissolved in DNA lysis buffer to release dsDNA, and samples were loaded onto a 1.5% DNA gel. After separation in the gel, images were captured using high‐performance fluorescence and chemiluminescence imaging systems (G: BOX; Chemi XX9).

### Enzyme‐Linked Immunosorbent Assay

After radiation treatment (radiation dose 40 Gy), resuspend the H22/CT26 cells (cell number 1 × 10^7^) in RPMI‐1640 culture medium containing either ML323/SJB2‐043 or DUB inhibitor (the inhibitors are shown in Figure S1 (Supporting Information), all at a concentration of 5 µm), then added them to the adherent BMDCs/DC2. 4/L929/RAW264.7 cells (cell number 5.0 × 10^6^), cocultured for 12 h. Afterward, collected the culture medium, and the supernatant was subjected to continuous centrifugation at 4 °C: first at 300 g for 10 min and then at 2000 g for 30 min to remove live and dead cells. The resulting supernatant was further centrifuged at 1000 g at 4 °C for 20 min. After a fivefold dilution of the collected supernatant, mouse IFN‐beta quantikine ELISA kits were used to detect secreted IFNβ1 following the instructions procedure.

For in vivo screening, CT26 cells were subcutaneously injected into the left axillary region of Balb/c mice at a dose of 1 × 10^6^ cells per mouse. Tumors typically form ≈1 week later. At this point, the mouse was dissected to obtain the whole tumor, and then cut tumors into small pieces. These cut‐tumor tissues were then cultured in RPMI‐1640 culture medium with or not with USP1 inhibitor ML323/SJB2‐043 (5 µm) and radiotherapy (radiation dose 8 Gy) was given within every 3 days. 24 hours after completing radiation therapy, collected the culture medium, then centrifuge it continuously at 4 °C: first at 300 g for 10 min, followed by 2000 g for 30 min to remove live and dead cells. After a fivefold dilution of the collected supernatant, mouse IFN‐beta quantikine ELISA kits and mouse CXCL10/IP‐10/CRG‐2 DuoSet ELISA kits were used to detect secreted IFNβ1and CXCL10 following the instructions procedure.

For serum samples obtained from animal experiments, whole blood samples collected in serum separator tubes, which were then stood at room temperature for 2 h. Afterward, the samples were followed centrifuged at 1000 g for 20 min at 4 °C to collect the serum. Then the serum fivefold was diluted and used the mouse IFN‐beta quantikine ELISA kits, mouse IFN‐gamma quantikine ELISA kits and mouse CXCL10/IP‐10/CRG‐2 DuoSet ELISA kits to detect the secreted IFNβ1, IFN‐γ, and CXCL10 following the instructions procedure.

### RNA Isolation and RT‐qPCR Analysis

RAW264. 7/L929/DC2. 4 cells were seeded in 6‐well dishes (5.0 × 10^5^ cells per well). Once the cells confluence reached to 80%, treated the cells with S‐cddA (100 nm)/cGAMP (100 nm)/DMXAA (2.5/5/10/25/50 µm) + ML323 (5 µm) for 2 h; or treated with ML323 (5 µm) + ISD90 (5 nm, transfected by NanoTrans Transfection Reagent Plus) for stimulation at time points 0/2/4/6/8 h; or treated with ISD90 (5 nm) + ML323 (0.1/0.5/1/2.5/5/10/20 µm) for 6 h; or treated with S‐cddA (100 nm) + ML323 (1/2.5/5/10/20 µm) for 2 h; or treated with S‐cddA (100 nm) combined with ML323 (5 µm) for stimulation at time points 0.5/1/1.5/2 h; or pretreated with H‐151 (5 µm) for 4 h, and then treated with S‐cddA (100 nm) + ML323 (5 µm) for another 2 h. After treatment by indicated time and concentration of compounds, total RNA was isolated by 1 mL VeZol Reagent. Then, add 200 µL of chloroform and centrifuge at 13 000 rpm for 15 min at 4 °C. Obtain the supernatant, add an equal volume of isopropanol, and then centrifuge at 13 000 rpm for 10 min at 4 °C. Discard the supernatant and add 500 µL of 75% ethanol (diluted by DEPC water) to the sediment at the bottom. Centrifuge at 13 000 rpm for 10 min at 4 °C. Discard the supernatant and add 20 µL of DEPC water. RNA concentration was determined by Nanodrop 2000 (Thermofisher) and 2 µg RNA was used to synthesized cDNA by HiScript Q RT SuperMix with gDNA wiper. Expression of respective genes was measured by RT‐qPCR using an equal amount of cDNA, performed on Bio‐Rad CFX96TM with ChamQ SYBR Color qPCR Master Mix. Data were presented at least three experiments. Use the Bio‐Rad CFX Manger Software to determine the relative gene expression and graphpad prism 8 was used to analysis the data.

The primer pairs are as follows:

The forward primer sequence for Mouse‐*Gapdh* is 5′‐ATTCAACGGCACAGTCAAGG‐3′, and the reverse primer sequence is 5′‐GCAGAAGGGGCGGAGATGA‐3′;

The forward primer sequence for Mouse*‐Ifnβ1* is 5′‐GGTGGAATGAGACTATTGTTG‐3′, and the reverse primer sequence is 5′‐AAGTGGAGAGCAGTTGAG‐3′;

The forward primer sequence for Mouse‐*Cxcl10* is 5′‐CCAAGTGCTGCCGTCATTTT‐3′, and the reverse primer sequence is 5′‐TTCATCGTGGCAATGATCTCAAC‐3′.

### Immunofluorescence Analysis

L929 ^mCherry‐STING^ cells or L929 cells were seeded in confocal culture dishes at a density of 2 × 10^4^ cells per dish. The drug treatment methods are as follow: L929 cells were treated with S‐cddA (300 nm) + ML323 (5 µm) for stimulation at time points 15/30/45/60 min; L929 ^mCherry‐STING^ cells or L929 cells were treated with S‐cddA (100 nm) + ML323 (5 µm) for 2 h; L929/DC2.4/RAW 264.7 cells were pretreated with ML323 (5 µm) for 2 h and then coincubated with IR‐treated (dose 40 Gy) H22 cells for another 12 h. After treatment of indicated time and concentration of drugs, cells were fixed for 15 min with 4% paraformaldehyde, permeabilized in 0.1% Triton X‐100 in PBS for 20 min and blocked using QuickBlock Immunostaining Blocking Solution for 1 h. Then, these cells were stained with indicated primary antibodies (STING, TBK1, ERGIC‐53/p58, GM130 were diluted at 1:100, dsDNA was diluted at 1:500) at 4 °C overnight and followed by incubated with fluorescent‐conjugated secondary antibodies at room temperature for 1 h. The nuclei were counterstained with DAPI for 5 min. Images were acquired by a confocal laser scanning microscope (Olympus, FV3000).

The colocalization analysis of IF images were conducted using the colocalization, 3D plugin, and Plot Profile tools in ImageJ software. The counting of intracellular fluorescent spots was completed using the Foci Quantification plugin in ImageJ software.

### Live‐Cell Imaging

L929/DC2.4/RAW 264.7 cells were seeded in confocal culture dishes at a density of 2 × 10^4^ cells per dish and pretreated with 5 µm ML323 for 2 h, and then coincubated with IR‐treated (dose 40 Gy) H22 cells (cell number 4 × 10^4^) for another 12 h. Before coincubated with L929/DC2.4/RAW 264.7 cells, H22 cells were first incubated with Nuc Red Live 647 Ready Probes at room temperature and protected from light for 30 min and washed with PBS for 3 times after IR‐treatment. The H22 cells were oscillatory washed away by PBS until no suspended or attached H22 cells were observed under microscope. Then the nuclei were stained with Hoechst 33 342 for 3 min, then washed three times with PBS. Above finished, the images were captured by a confocal laser scanning microscope (Olympus FV3000).

The counting of intracellular fluorescent spots was completed using the Foci Quantification plugin in ImageJ software.

### Immunoblotting Assays

1 × 10^6^ DC2. 4/L929/RAW264. 7 cells were seeded and adhered; the cells are then treated with S‐cddA (100 nm) + ML323 (5 µm) for stimulation at time points 0. 5/1/1.5/2 h; or treated with S‐cddA (100 nm) + ML323 at a concentration gradient (1/2.5/5/7.5 µm or 0. 5/1/2.5/10 µm) for 2 h; or treated with S‐cddA (15/30/60/125/250 nm) + ML323 (5 µm) for 2 h. Furthermore, 1 × 10^6^ L929 cells were seeded per dishes, once the cells have adhered, treated these cells with S‐cddA (100 nm/1000 nm)/ML323 (5 µm)/S‐cddA (100 nm) + ML323 (5 µM) for 2 h, while another group was first stimulated with BFA (2 mm) for 2 h followed by adding S‐cddA (100 or 1000 nm) + BFA (2 mm)/ML323 (5 µm) + BFA (2 mm)/S‐cddA (100 nm) + ML323 (5 µm) + BFA (2 mm) for an additional 2 h; or treated with S‐cddA (100 nm)/S‐cddA (100 nm) + ML323 (5 µm) for stimulation at time points 0/0.5/1/1.5/2 h, another group was first stimulated with BFA (2 mm) for 2 h, and followed by treating with S‐cddA (100 nm)/S‐cddA (100 nm) + ML323 (5 µm) for additional stimulation at time points 0/0.5/1/1.5/2 h.

For in vivo experiments, CT26 cells were subcutaneously injected into the left axillary region of Balb/c mice at a dose of 1 × 10^6^ cells per mouse. Tumors typically form ≈1 week later. At this point, the mouse was dissected to obtain the whole tumor, and then cut tumors into small pieces. These cut‐tumor tissues were then cultured in RPMI‐1640 culture medium with or without USP1 inhibitor ML323/SJB2‐043 (5 µm), and radiotherapy (radiation dose 8 Gy) was given within every 3 days; 24 h after completing radiation therapy, freeze the tumor in liquid nitrogen and then put it into a homogenizer, adding precooled RIPA lysis buffer for homogenization.

Whole cell‐extracts were diluted in RIPA lysis buffer with 1 × phosphatase inhibitors and 1 × PMSF. Then centrifuging the lysate at 13 000 rpm for 15 min at 4 °C, and collected the supernatant, quantified to 40 ng/16 µL by BCA protein assay kit. Subsequently, 5 × SDS‐PAGE sample loading buffer was added, and the mixture was heated at 95 °C for 15 min, then loaded the mixture into 10%–15% SDS‐PAGE gel. After separated, proteins were transferred onto PVDF membranes. The membranes were subsequently incubated overnight at 4 °C with primary antibodies (STING, Phospho‐STING, TBK1, Phospho‐TBK1, LC3A/B, USP1, SEC24C, SEC23A, VPS34, STEEP, SAR1A, Phospho‐mTOR, p62, Phospho‐p62, PDL1, GAPDH, Vinculin, β‐Tubulin were diluted at 1:1000), followed by incubation with a horseradish peroxidase‐coupled secondary antibody for 2 h at room temperature. Then the protein detection was accomplished using the FDbio‐Dura ECL Kit and quantified using Image J.

### Coimmunoprecipitation Assays

4.5 × 10^6^ L929 cells were seeded and adhered, then were stimulated with S‐cddA (100 nm)/ML323 (5 µm)/S‐cddA (100 nm) + ML323 (5 µm) at time points 1 or 2 h; 4.5 × 10^6^ L929^HA‐UB‐K27^ or L929^HA‐UB‐K27R^ cells were seeded and adhered, then were treated with S‐cddA (100 nm)/S‐cddA (100 nm) + ML323 (5 µm) for stimulation at time points 0/0.25/0.5/1/1.5/2 h; 4.5 × 10^6^ L929^HA‐UB‐K27^ cells were seeded and adhered, were then treated with b‐AP15M/ML364/P005091/DUB‐IN‐2/Degrasyn/Mitoxantrone/Spautin‐1/PT33/MF‐094/GSK264394A/ML323 (5 µm) + S‐cddA (100 nm) + B‐AP15M/ML364/P005091/DUB‐IN‐2/Degrasyn/Mitoxantrone/Spautin‐1/PT33/MF‐094/GSK264394A/ML323 (5 µm). Then the whole cell‐extracts were diluted in IP lysis buffer with 1 × phosphatase inhibitors and 1 × PMSF. followed by centrifuged for 10 min, 14 000 rpm at 4 °C after lysing at 4 °C for 30–60 min. Once centrifuged finished, the supernatant was collected and quantified to a total protein volume of 1 mg/500 µL. Then added 1/500 µL of primary antibody to the protein, and incubated overnight at 4 °C in a rotating shaker. After the incubation was completed, 20 µL of Protein A + G Agarose, previously washed three times with TBS at 4 °C, was added, and the mixture was incubated at 4 °C for an additional 4 h. After incubation, the Protein A + G Agarose was washed with TBS at 4 °C for three times, then added 1 × loading buffer and heated at 95 °C for 15 min, then proceed with WB analysis.

4.5 × 10^6^ L929^HA‐UB‐WT^/L929^HA‐UB‐K6^/L929^HA‐UB‐K11^/L929^HA‐UB‐K27^/L929^HA‐UB‐K29^/L929^HA‐UB‐K33^/L929^HA‐UB‐K48^/L929^HA‐UB‐K63^ cells were seeded and adhered, then stimulated with S‐cddA (100 nm)/ML323 (5 µm)/S‐cddA (100 nm) + ML323 (5 µm) for 3 h. Furthermore, 4.5 × 10^6^ L929^USP1‐Flag^ cells were seeded and adhered, then treated with S‐cddA (100 nm)/S‐cddA (100 nm) + ML323 (5 µm) for stimulation at time points 0/0.25/0.5/1/1.5 h. Then the whole cell‐extracts were diluted in IP lysis buffer and centrifuged for 10 min, 14 000 rpm at 4 °C after lysing at 4 °C for 30–60 min. The supernatant was collected and quantified to a total protein volume of 1 mg/500 µL. Take 500 µL of quantified protein from each group, add 20 µL of magnetic beads that have been replaced with TBS at 4 °C three times, followed by incubation overnight at 4 °C in a rotating shaker. After incubation, washed the beads with TBS at 4 °C for three times. Then added 1 × loading buffer and heated at 95 °C for 15 min, then proceed with WB analysis.

### Mice and In Vivo Studies

Female Balb/c mice (stock number N000020‐22) and female BALB/cJNju‐Foxn1nu/Nju(stock number D000521) were purchased form Gempharmatech‐GD, Guangzhou, China.

### Bilateral Axillary Tumor Cell Implantation

CT26 cells were implanted bilaterally under the axilla of Balb/c mice (4–5 weeks), with a cell count of 1 × 10^6^ per site. The mice were randomly divided into the following groups: blank control group, ML323 group, radiation therapy group, and combination of radiation therapy with ML323 group. ML323 at a dose of 5 mg kg^−1^ (dissolved in 1% DMSO and diluted in HS‐15, then prepared into a 94% NS solution) was intraperitoneally injected one day prior to radiation therapy and administered radiation therapy with a daily dose of 8 Gy for 5 days. After radiation therapy, recorded the tumor volume. In order to comply with animal ethics requirements, on the 15th day, the mice in control and ML323 groups were euthanized, and tumors were removed; and on the 23rd day, the mice in radiation therapy group and combination of radiation therapy with ML323 group were euthanized. After complete removal of the tumors, fixed them with 4% paraformaldehyde and then performed HE and IHC staining analysis on the sections. The tumor volume was calculated by the following formula: Volume = 0.5 × width^2^ × length.

### Axillary Tumor Cell Implantation

CT26 cells were implanted in the axilla of female Balb/c mice with a tumor cell count of 1 × 10^6^ per mouse. The mice were randomly divided into the following groups: radiation therapy group, and combination of radiation therapy with ML323 group. ML323 was administered intraperitoneally at a dose of 5 mg kg^−1^ daily for 7 days before the initiation of radiation therapy. Beginning on day 9, radiation therapy will be delivered intraperitoneally at a dose of 2 Gy daily for a total of 7 days. During this period, ML323 will be continuously intraperitoneally injected once a day. Additionally, ML323 was given for 5 days after the completion of radiation therapy. After radiation, PDL1 (5 mg kg^−1^) were administered every 2 days for a total of three intraperitoneal injections, then tumor volume was recorded, the mice were euthanized on the 37th day and removed the tumors, fixed the tumors with 4% paraformaldehyde and then performed HE and IHC staining analysis on the sections.

### Mice Survival Rate Studies

Another batch of female Balb/c mice were purchased and performed tumor transplantation, grouping, administered intraperitoneally, and RT according to the experimental protocol for unilateral axillary tumors mentioned above. Tumor volumes were recorded, and the mice were euthanized when the tumor volume reaches 2000 mm^3^. Survival rate of each group of mice were recorded every day.

### HE and Immunohistochemistry (IHC) Assays

After fixing the tumor tissue in 4% paraformaldehyde for 30 min, it is sequentially immersed in ethyl alcohol solutions of varying concentrations (70%, 80%, 95%, 100%) for 30 min each, followed by a process of clearing with xylene. The cleared tumor tissue was then placed in a melted paraffin, allowing it to fully saturate before placing it on a clean paraffin block. The paraffin is then heated to solidify, forming a paraffin block containing the tissue. Finally, the tumor tissue is cut into thin slices using a microtome, and the slices are adhered to a slide with Poly‐L‐lysine.

After heating paraffin sections in a 60 °C oven for 2 h, immerse them in xylene twice for 10 min each, sequentially immerse in absolute ethanol, 90% ethanol, 80% ethanol, 75% ethanol, and ddH_2_O for 10 min each for hydration, and then added the hematoxylin staining solution dropwise and stain for 5 min. Rinse off the hematoxylin stain with running water, then use 1% acetic acid alcohol for differentiation for 3 s. Soak the sections in running water for 30 min to counterstain the blue. Then, add 1 drop of 0.5% eosin staining solution to each section, stain for 15 s, and rinse with distilled water three times, each for 5 min. Then immerse the sections in 75% ethanol, 80% ethanol, 90% ethanol, absolute ethanol, and xylene for dehydration. After the xylene has completely evaporated, seal the sections with neutral gum.

After heating paraffin sections in a 60 °C oven for 2 h, immerse them in xylene twice for 10 min each, sequentially immerse in absolute ethanol, 90% ethanol, 80% ethanol, and 75% ethanol for hydration, perform antigen retrieval using EDTA, wait for the sections to return to room temperature, then block with 20% goat serum, incubate with the primary antibody at 4 °C overnight, then incubate with goat antimouse/rabbit IgG polymer at room temperature for 30 min. Perform staining with a DAB substrate, then restain with hematoxylin. After washing with 1% acetic acid ethanol, soak the sections in running water for 30 min to counterstain the blue, after that, immerse the sections in 75% ethanol, 80% ethanol, 90% ethanol, absolute ethanol, and xylene for dehydration. After the xylene has completely evaporated, seal the sections with neutral gum.

HE and IHC images were acquired by scanning with a Fully Automatic Digital Pathology Slide Scanner (Kfbio/KF‐PRO‐020). After randomly selecting one field of view at 4 × magnification, 5 additional field of views at 20 × magnification is randomly selected from the same region. The scores of all images are then comprehensively evaluated.

### Statistical Analysis

Clinical statistical analysis was performed using SPSS 20.0 software. Survival times for patient groups were assessed using the log‐rank test, Kaplan–Meier curves. The relationship between post‐RT CD8/Granzyme B/Perforin and pre‐RT USP1 level was evaluated using Spearman correlation analysis. A *p*‐value of less than 0.05 was considered statistically significant.

All in vitro and vivo experiments statistical analysis was performed using GraphPad Prism 8.0 software. Differences in the average between two groups with one variate were determined by Student's *t*‐test. Differences in the average between more than two groups with one variate were determined by one‐way analysis of variance (ANOVA). Differences between groups with dose factors or with more than one variate were determined by two‐way ANOVA. Survival was analyzed using the log‐rank test.

Additional Experimental Section can be found in the Supporting Information. All information regarding reagents, plasmids, antibodies, and cell constructs can be found in Table S3 (Supporting Information). Abbreviations can be found in Table S4 (Supporting Information).

## Conflict of Interest

The authors declare no conflicts of interest.

## Author Contributions

W.Z., Y.Z., W.Q., and W.W. contributed equally to this work. All authors participated in data acquisition. X.Y., X.B., X.W. contributed to the conception and design of the study. W.Z., Y.Z., W.Q., W.W., C.L., Y.Z., X.Y., X.C., Y.W., Y.K., J.W., J.Z., J.Q. did the data analysis and interpretation. X.Y., X.B., X.W., W.Z., Y.Z., J.Q. contributed to the drafting and revision of the manuscript. All authors read and approved the final manuscript.

## Supporting information



Supporting Information

## Data Availability

All data are available within the article, supplementary information or available from the corresponding author upon reasonable request.
