# Supplementary material for: Targeting USP1 Potentiates Radiation‐Induced Type I IFN‐Dependent Antitumor Immunity by Enhancing Oligo‐Ubiquitinated SAR1A‐Mediated STING Trafficking and Activation
Source: Adv Sci (Weinh). 2025 Feb 20;12(15):2412687. doi: 10.1002/advs.202412687 (PMC12005740; doi:10.1002/advs.202412687)
Supplement: Supplementary file 1 — Supporting Information [file ADVS-12-2412687-s001.pdf]

ADVANCED SCIENCE
Open Access

Supporting Information

for *Adv. Sci.*, DOI 10.1002/advs.202412687

Targeting USP1 Potentiates Radiation-Induced Type I IFN-Dependent Antitumor Immunity by Enhancing Oligo-Ubiquitinated SAR1A-Mediated STING Trafficking and Activation

Weilin Zhou, Yuxuan Zhao, Wenjing Qin, Weijian Wu, Chenyang Liao, Yiqiu Zhang, Xingli Yang, Xue Chen, Youqiao Wang, Yushan Kang, Jiaxin Wu, Jiaojiao Zhao, Junmin Quan, Xuecen Wang, Xianzhang Bu* and Xin Yue**

Supplementary Information for

Targeting USP1 potentiates Radiation-induced Type I IFN-dependent Antitumor immunity by Enhancing Oligo-ubiquitinated SAR1A-mediated STING Trafficking and Activation

Weilin Zhou, Yuxuan Zhao, Wenjing Qin, Weijian Wu, Chenyang Liao, Yiqiu Zhang, Xingli Yang, Xue Chen, Youqiao Wang, Yushan Kang, Jiaxin Wu, Jiaojiao Zhao, Junmin Quan, Xuecen Wang*, Xianzhang Bu*, and Xin Yue*.

This file contains:

Supplementary Figures S1-S26

Supplementary Tables S1-S4

Supplementary Experimental Section

Supplementary Figures

Figure S1

A

Inhibitors	Targets
LDN57444	UCH-L1; UCH-L3; UCH-L5
b-AP15	UCH-L5; USP14
ML323	USP1
ML364	USP2
XL177A	USP7
DUB-IN-2	USP8
Degrasyn	USP9X; USP5
Mitoxantrone	USP11
Spautin1	USP10; USP13
PT33	BAP1; USP14
USP15-IN-1	USP15
GSK264394A	USP20
USP22-IN-1	USP22
USP28-IN-3	USP28
MF-094	USP30
USP7/47-IN-1	USP7; USP47
OTUB1/USP8-IN-1	OTUB1; USP8

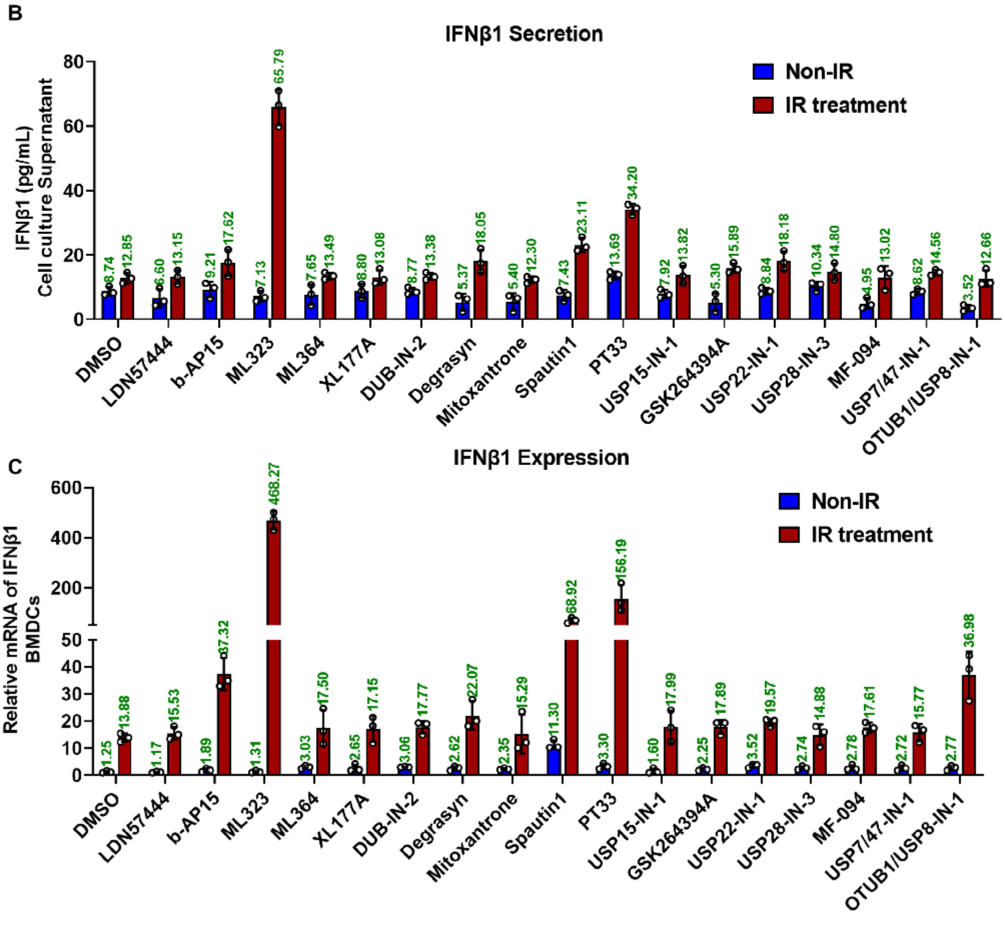

Figure S1. Screening DUB inhibitors to enhance innate immune effects following RT, related to Figures 1A-C. (**A**) Selected DUB inhibitors and their corresponding targets. (**B**) Quantification of IFNβ1 secretion in the supernatant of co-cultured BMDCs treated with various DUB inhibitors using ELISA assay. (**C**) Expression levels of *Ifnβ1* in co-cultured BMDCs measured by RT-qPCR. Data were represented as mean ± S.D. (n=3).

Figure S2

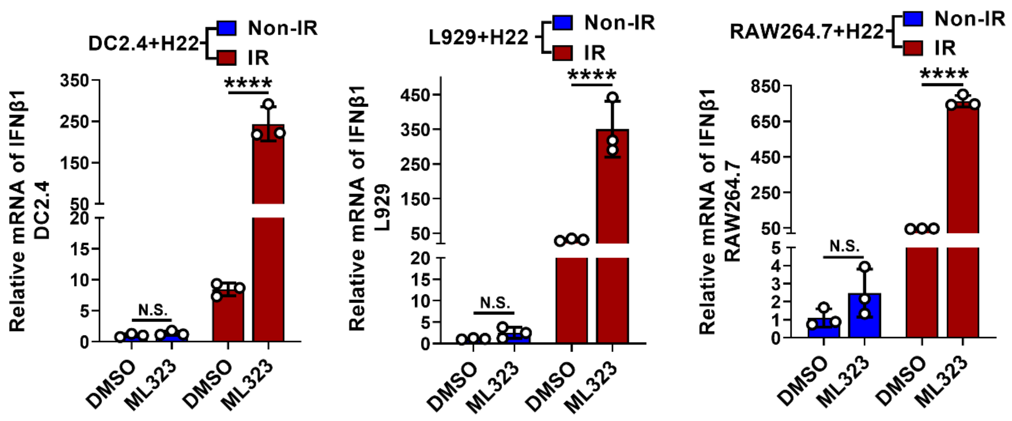

Figure S2. Effects of USP1 inhibitor ML323 on IFNβ1 expression in co-culture experiments of DC2.4, L929, and RAW264.7 cells with irradiated H22 cells, related to Figure 1B. Co-treatment with ML323 showed elevated **expression** levels compared to the RT group. Data were represented as mean ± S.D. (n=3). Statistical significance was determined by two-way ANOVA (N.S., no significance; ****, P<0.0001).

Figure S3

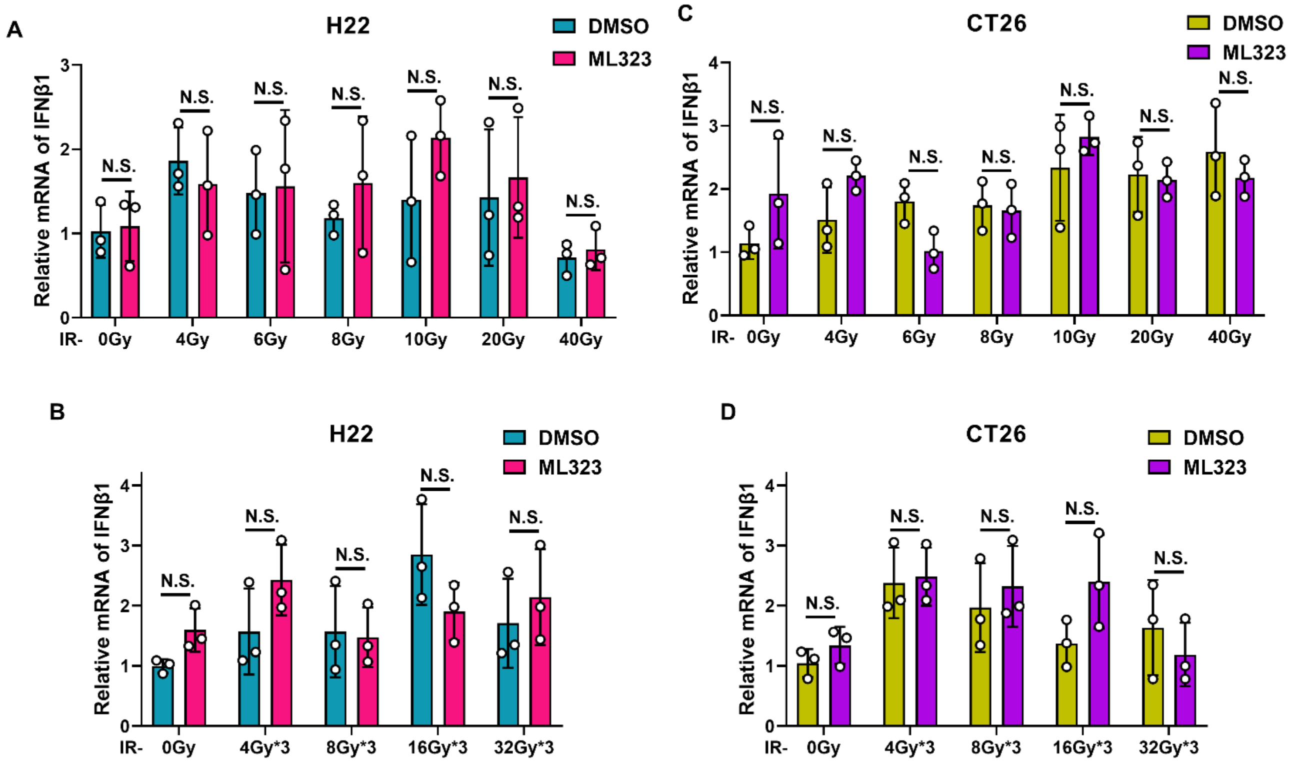

Figure S3. ML323 does not significantly promote the expression of IFNβ1 induced by RT of tumor cells. H22 and CT26 cells were treated with indicated doses of radiotherapy and combined with ML323 (5 μM). The expression level of *Ifnβ1* was detected by RT-qPCR assay. Data were represented as mean ± S.D. (n=3). Statistical significance was determined by two-way ANOVA (N.S., no significance).

Figure S4

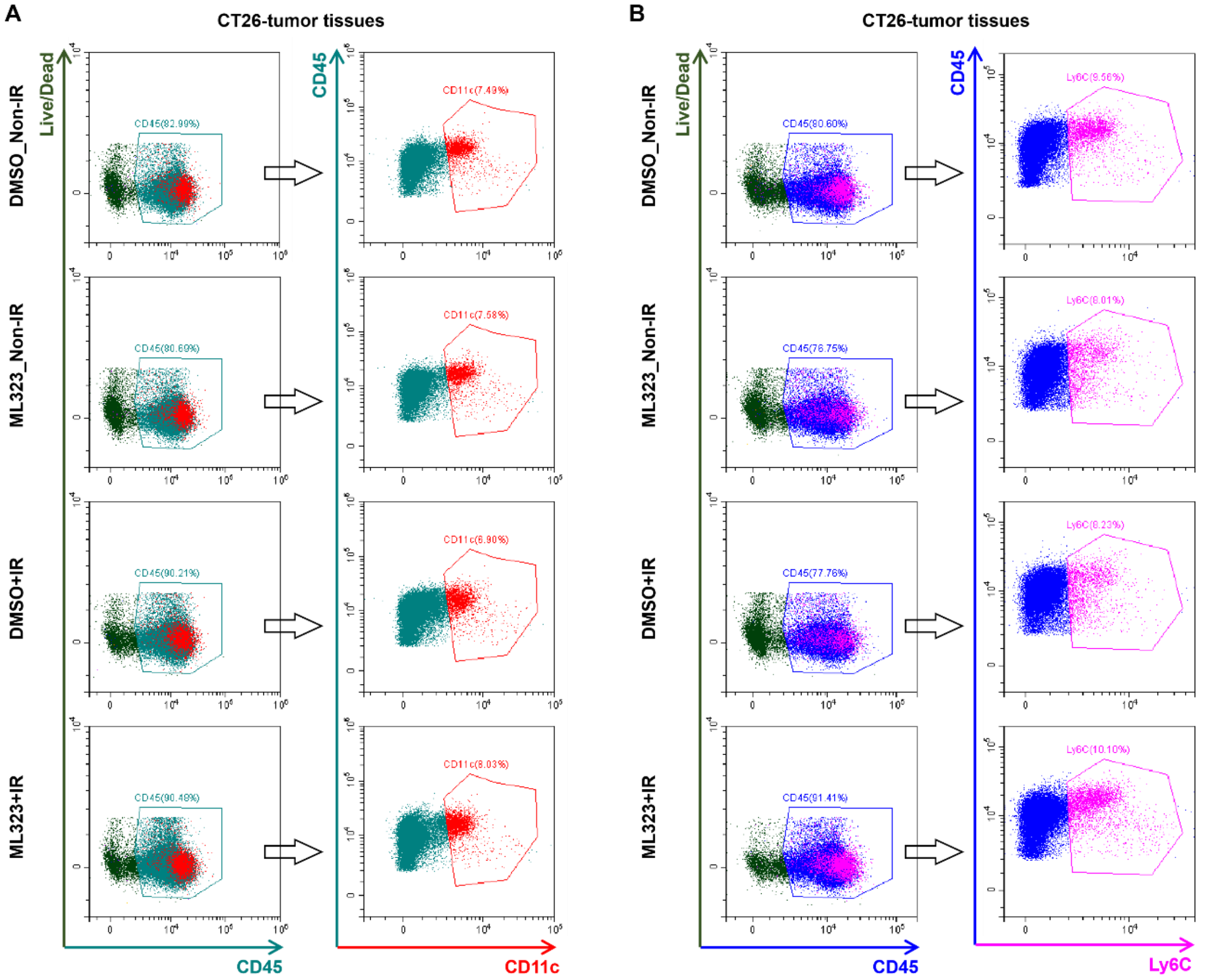

Figure S4. Single-cell suspensions from tumors treated with C-1 were stained with conjugated antibodies against CD45, CD11c, and Ly6C, followed by sorting into different cell populations using flow cytometry. Flow cytometry analysis of CD11c^{+} (**A**) and Ly6C^{+} (**B**) cells in CD45^{+} cells from CT26-tumor tissues. Immune cell populations are identified based on CD45 expression, **related to Figure 1C-4/5**.

Figure S5

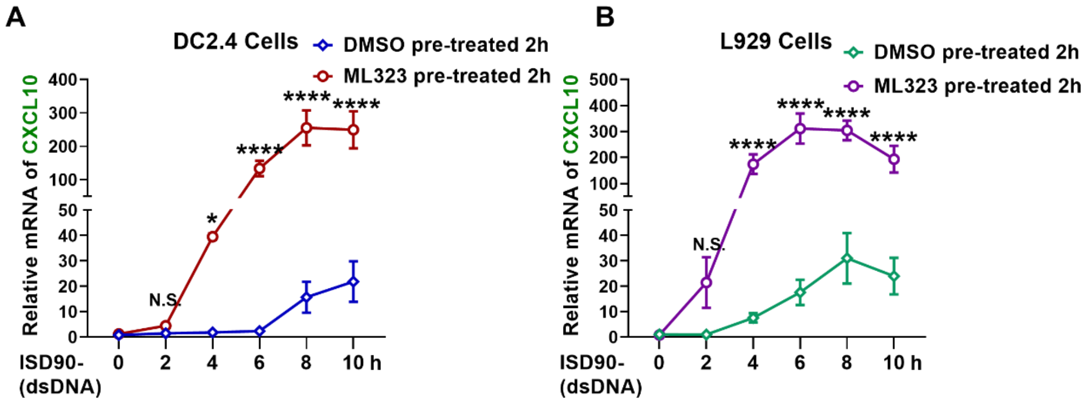

Figure S5. ML323 enhances dsDNA induced response of type I IFNs, related to Figure 2A-2. (A-B) DC2.4/L929 cells were treated by ISD90 (5 nM) or ISD90 combined with ML323 (5 μM, pretreated 2 h) for the indicated time, relative expression levels of *Ifnβ1* and *Cxcl10* were detected by RT-qPCR assay. Data were represented as mean ± S.D. (n=3). Statistical significance was determined by two-way ANOVA (N.S., no significance; *, P<0.05; ****, P<0.0001).

Figure S6

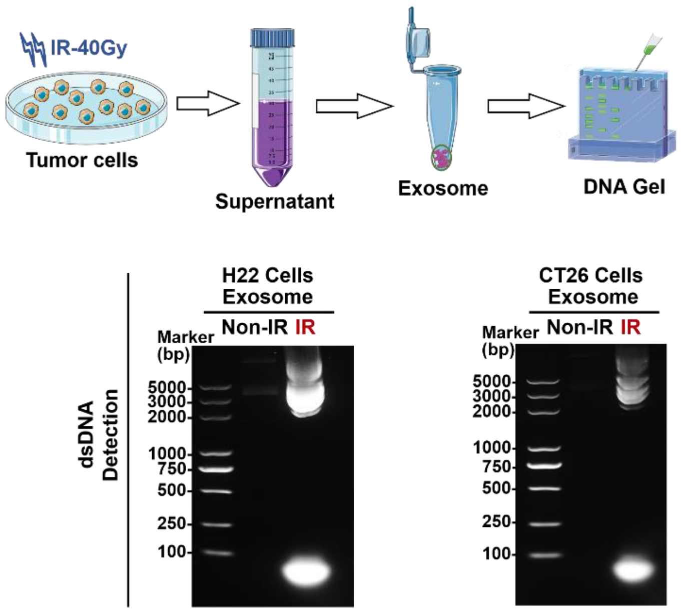

Figure S6. The secretion of exosomes from tumor cells after radiotherapy treatment. After being treated with RT at a dose of 40 Gy, H22 and CT26 cells were cultured for 12 hours. Exosomes were then extracted from the supernatant culture medium and detected by DNA gel separation.

Figure S7

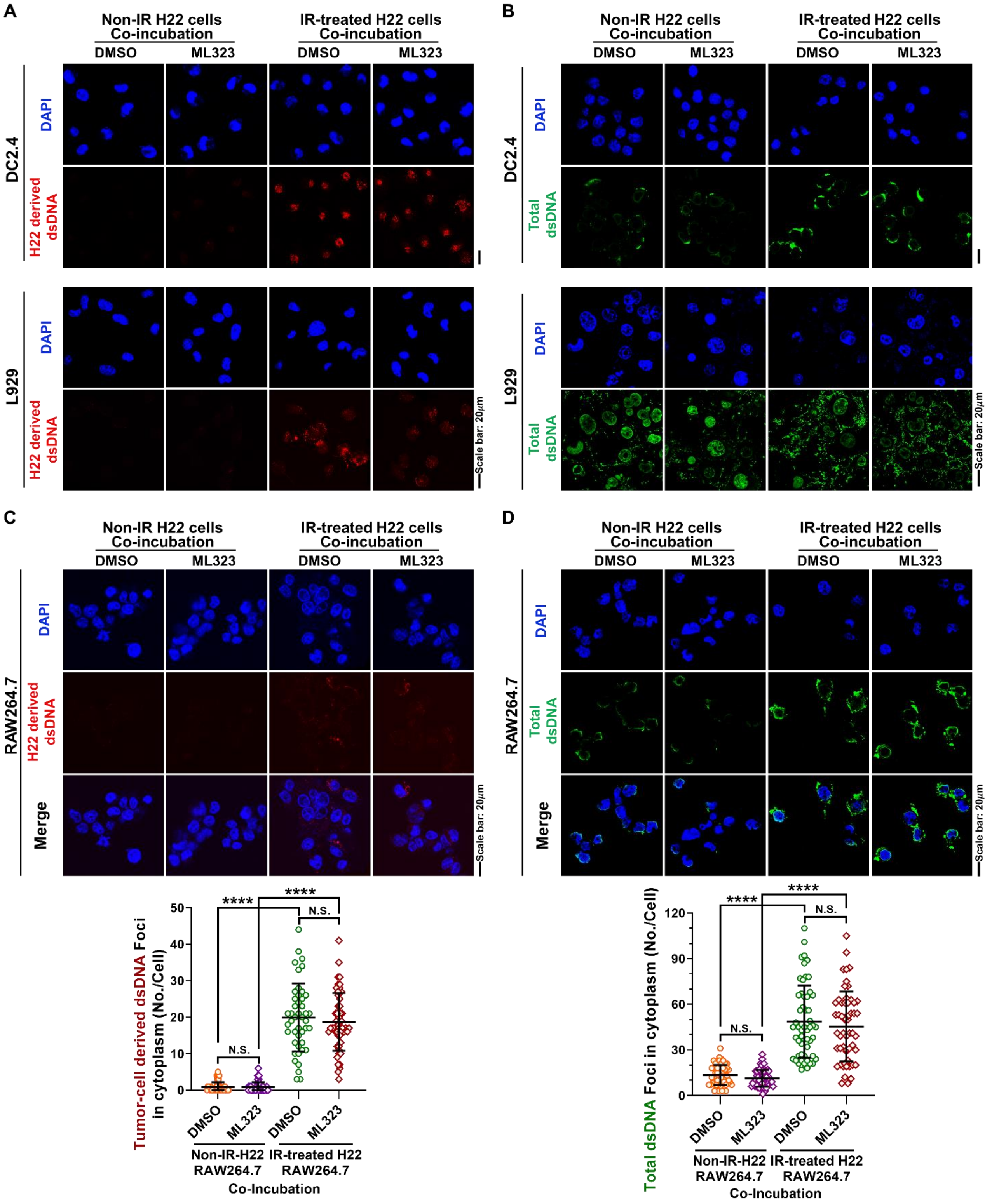

Figure S7. **Characterization of tumor cell-derived dsDNA and total dsDNA content and distribution.** (**A**) Imaging of dsDNA content and distribution within DC2.4 and L929 cells after co-incubation with H22-derived dsDNA in live cells, **related to Figure 2C-2**. (**B**) IF assay analysis of

dsDNA content and distribution within DC2.4 and L929 cells after co-incubation, **related to Figure 2C-4.** (**C**) Imaging of dsDNA content and distribution within RAW264.7 cells after co-incubation with H22-derived dsDNA in live cells. (**D**) IF assay analysis of dsDNA content and distribution within RAW264.7 cells after co-incubation. The statistical method for cell imaging includes results from all biological replicates (n=3). Statistical significance was determined by (**C** and **D**) one-way ANOVA (N.S., no significance; ****, P<0.0001).

Figure S8

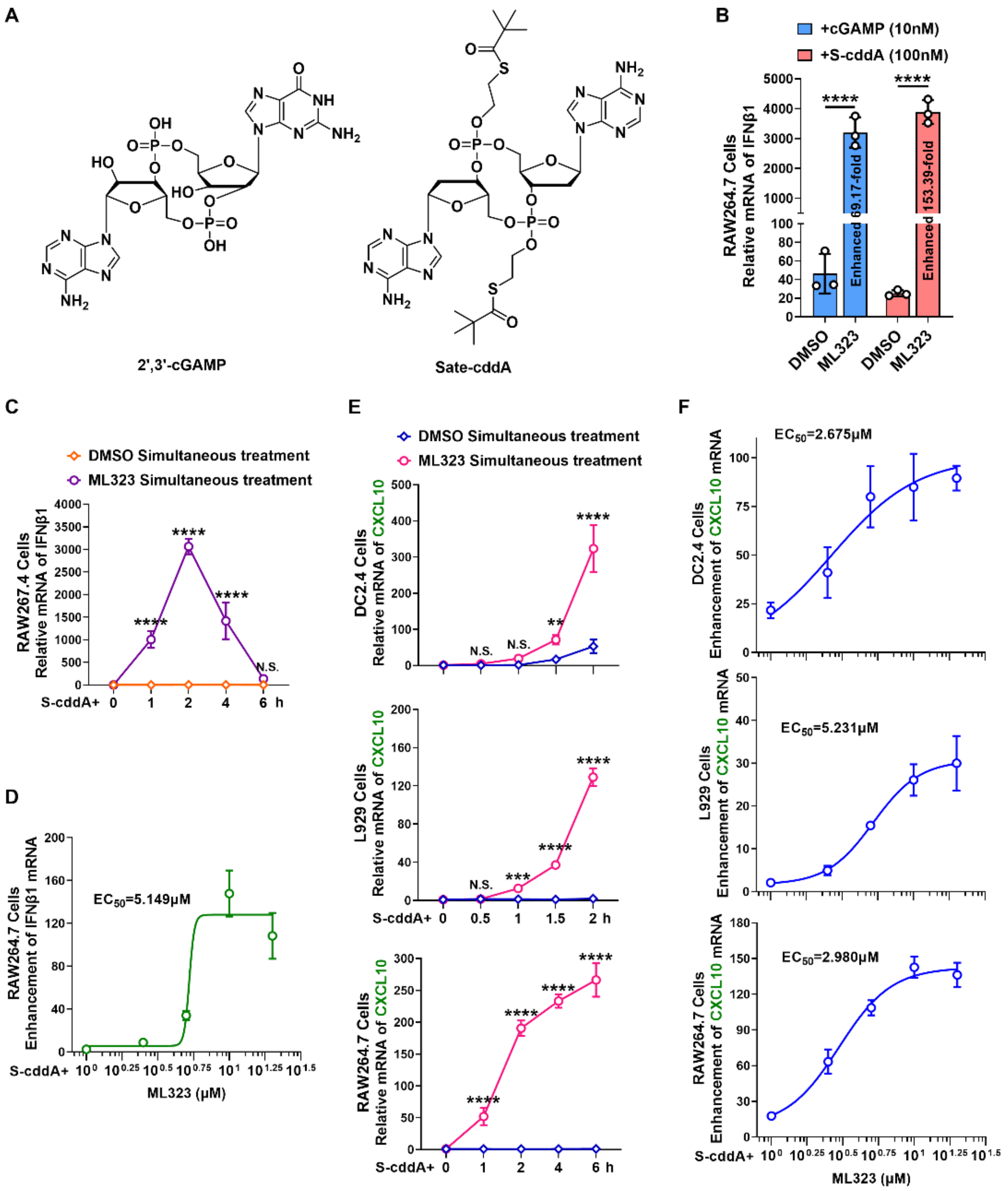

Figure S8. ML323 synergistically activates the STING-mediated type I IFN responses, related to Figures 2E-G. (**A**) Structure of 2’,3’-cGAMP and Sate-cddA (S-cddA). (**B**) RAW264.7 cells were treated by STING agonist cGAMP or S-cddA, as well as combined with ML323, relative expression levels of *Ifnβ1* were detected by RT-qPCR assay. (**C**) RAW264.7 cells were treated by S-cddA (100 nM) combined with ML323 (5 μM) for the indicated time, relative expression levels of *Ifnβ1* were detected by RT-qPCR assay. (**D**) RAW264.7 cells were treated by S-cddA (100 nM) combined with the indicated concentration of ML323, relative expression levels of *Ifnβ1* were detected by RT-qPCR

assay and EC_{50} value were calculated. (**E**) DC2.4/L929/RAW264.7 cells were treated by S-cddA (100 nM) combined with ML323 for the indicated time, relative expression levels of *Cxcl10* were detected by RT-qPCR assay. (**F**) DC2.4/L929/RAW264.7 cells were treated by S-cddA (100 nM) combined with the indicated concentration of ML323, relative expression levels of *Cxcl10* were detected by RT-qPCR assay and EC_{50} value were calculated. Data were represented as mean ± S.D. (n=3). Statistical significance was determined by two-way ANOVA (N.S., no significance; **, P<0.01; ***, P<0.001; ****, P<0.0001).

Figure S9

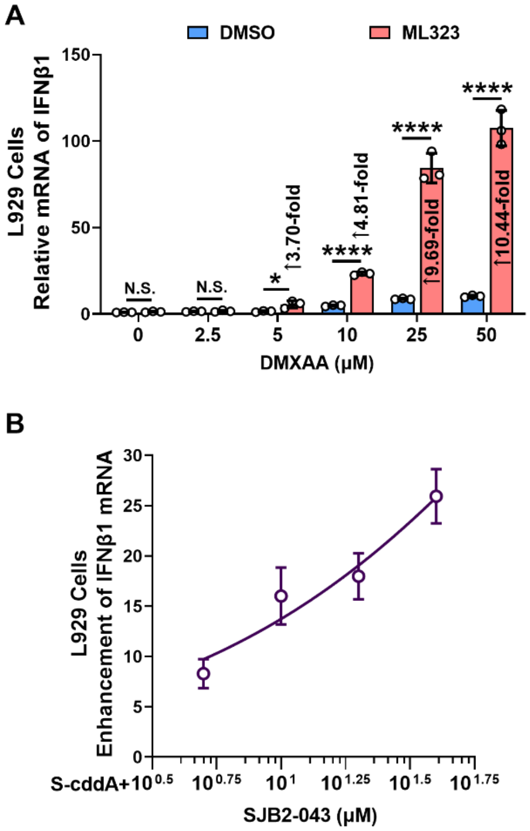

Figure S9. USP1 inhibitors can synergize with different STING agonists to enhance type I IFN responses. (**A**) In L929 cells, ML323 (5 μM) was co-incubated with the indicated concentrations of STING agonist DMXAA, and the relative expression level of *Ifnβ1* was detected by RT-qPCR assay. (**B**) In L929 cells, S-cddA (100 nM) was co-incubated with different concentrations of USP1 inhibitor SJB2-043, and the relative expression level of *Ifnβ1* was detected by RT-qPCR assay. Data were represented as mean ± S.D. (n=3), Statistical significance was determined by two-way ANOVA (N.S., no significance; *, P<0.05; ****, P<0.0001).

Figure S10

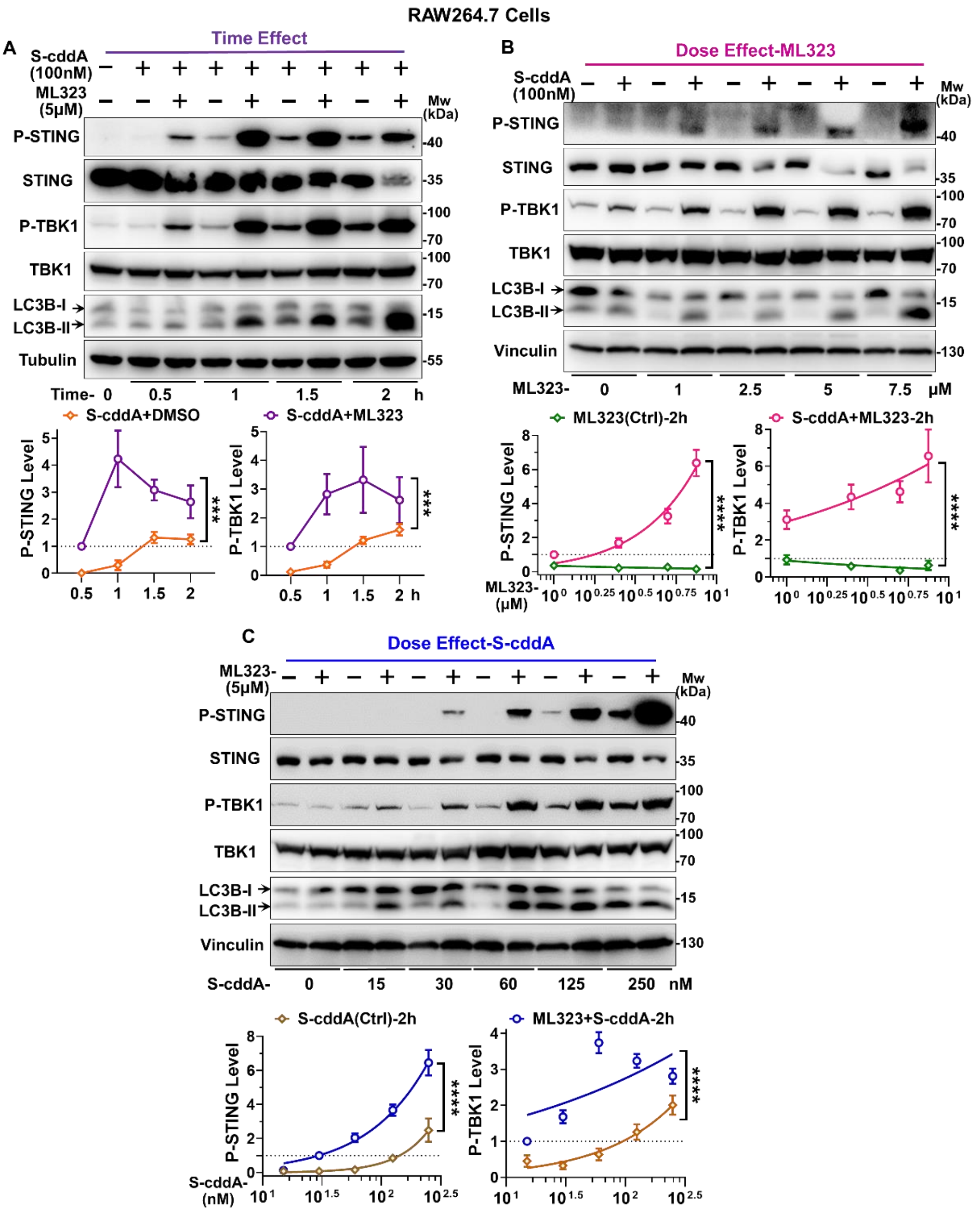

Figure S10. Inhibition of USP1 with ML323 enhances STING pathway activation and promotes LC3 lipidation in RAW264.7 cells. (**A**) Time-course analysis of S-cddA alone or in combination with ML323 revealed variations in P-STING, P-TBK1, and LC3B-II/LC3B-I levels. (**B**) Dose-response analysis of ML323 showed a dose-dependent increase in P-STING, P-TBK1, and LC3B-II/LC3B-I levels when combined with S-cddA. (**C**) Dose-response analysis of S-cddA revealed a dose-dependent increase in P-STING, P-TBK1, and LC3B-II/LC3B-I levels when combined with ML323. Quantifications of grayscale values (below), data were represented as mean ± S.D. (n=3). Statistical significance was determined by two-way ANOVA (***, P<0.001; ****, P<0.0001).

Figure S11

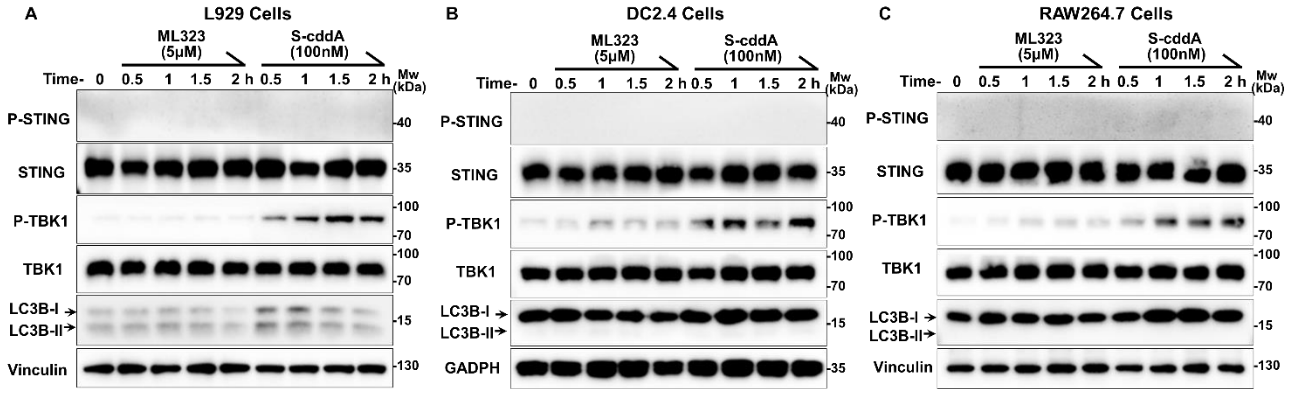

Figure S11. ML323 alone does not have an activating effect on cGAS-STING signaling pathway. WB analysis in L929 (**A**), DC2.4 (**B**), RAW264.7 (**C**) cells treated by ML323 or S-cddA for 0.5, 1.0, 1.5, 2.0 hours. All data were obtained from biological replicates conducted more than 3 times.

Figure S12.

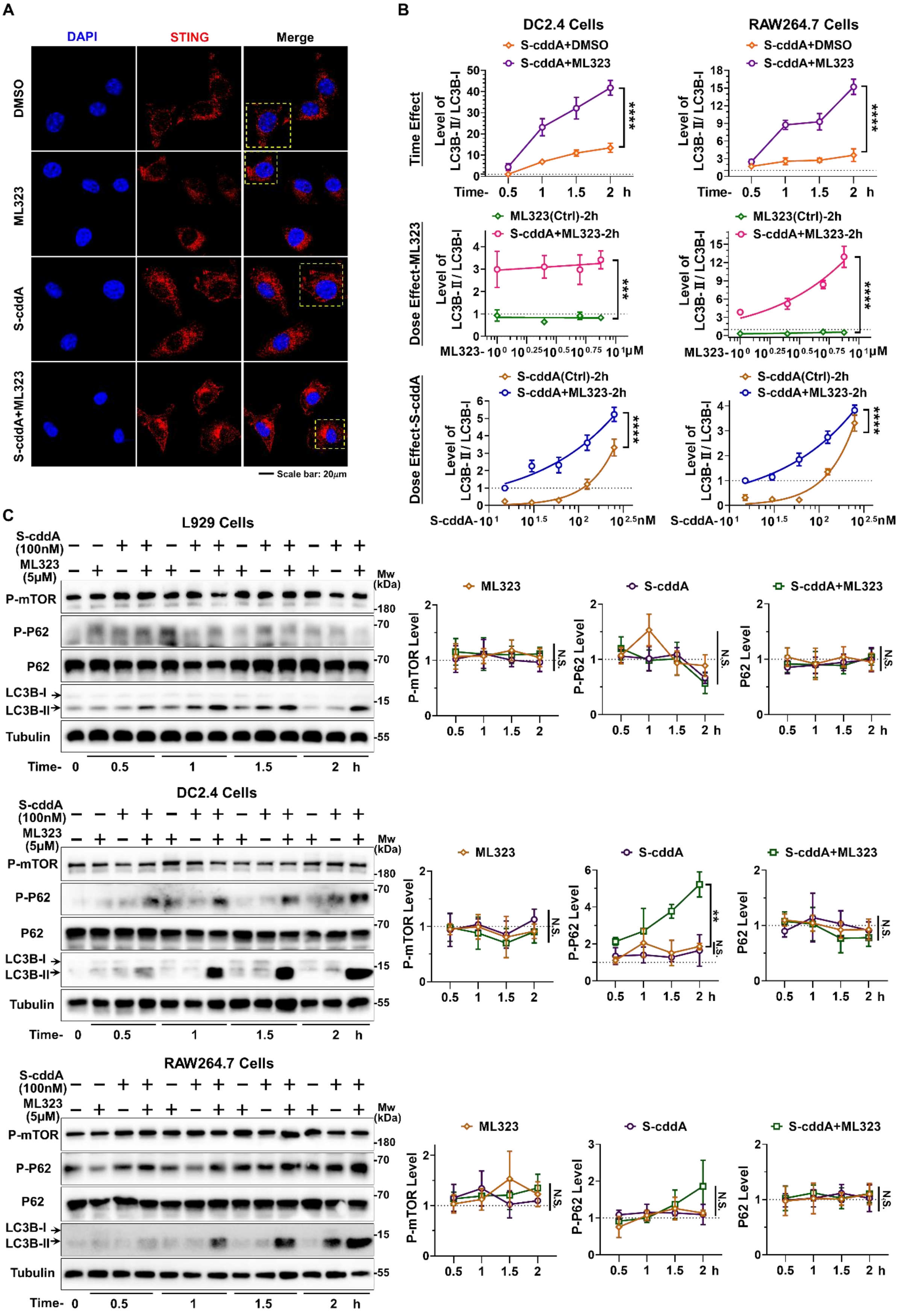
A
DAPI
STING
Merge
DMSO
ML323
S-cddA
S-cddA+ML323
Scale bar: 20μm
B
DC2.4 Cells
RAW264.7 Cells
S-cddA+DMSO
S-cddA+ML323
Time Effect
Level of LC3B-II/ LC3B-I
Time- 0.5 1 1.5 2 h
ML323(Ctrl)-2h
S-cddA+ML323-2h
Dose Effect-ML323
ML323-
μM
S-cddA(Ctrl)-2h
S-cddA+ML323-2h
Dose Effect-S-cddA
S-cddA-
nM
C
L929 Cells
DC2.4 Cells
RAW264.7 Cells
S-cddA (100nM)
ML323 (5μM)
Mw (kDa)
P-mTOR
P-P62
P62
LC3B-I
LC3B-II
Tubulin
-180
-70
-15
-55
ML323
S-cddA
S-cddA+ML323
P-mTOR Level
P-P62 Level
P62 Level
N.S.

Figure S12. The autophagic activity induced by ML323 combined with S-cddA. (**A**) Immunofluorescence analysis of distribution of STING in L929 cells treated by S-cddA (100 nM) combined with ML323 (5 μM), **related to Figure 4A.** (**B**) Grey value analysis of relative level of LC3B-II/LC3B-I in DC2.4 cells and RAW264.7 cells, **related to Figure 3.** Statistical significance was determined by two-way ANOVA, (N.S., no significance; **, P<0.01; ***, P<0.001; ****, P<0.0001). (**C**) WB analysis of LC3B-II/LC3B-I, P62, P-P62 and P-mTOR in L929, DC2.4 and RAW264.7 cells treated by ML323 (5 μM) and S-cddA (100 nM) for 0.5, 1.0, 1.5 and 2.0 hours, and relative grey value analysis. All data were obtained from biological replicates conducted more than 3 times.

Figure S13

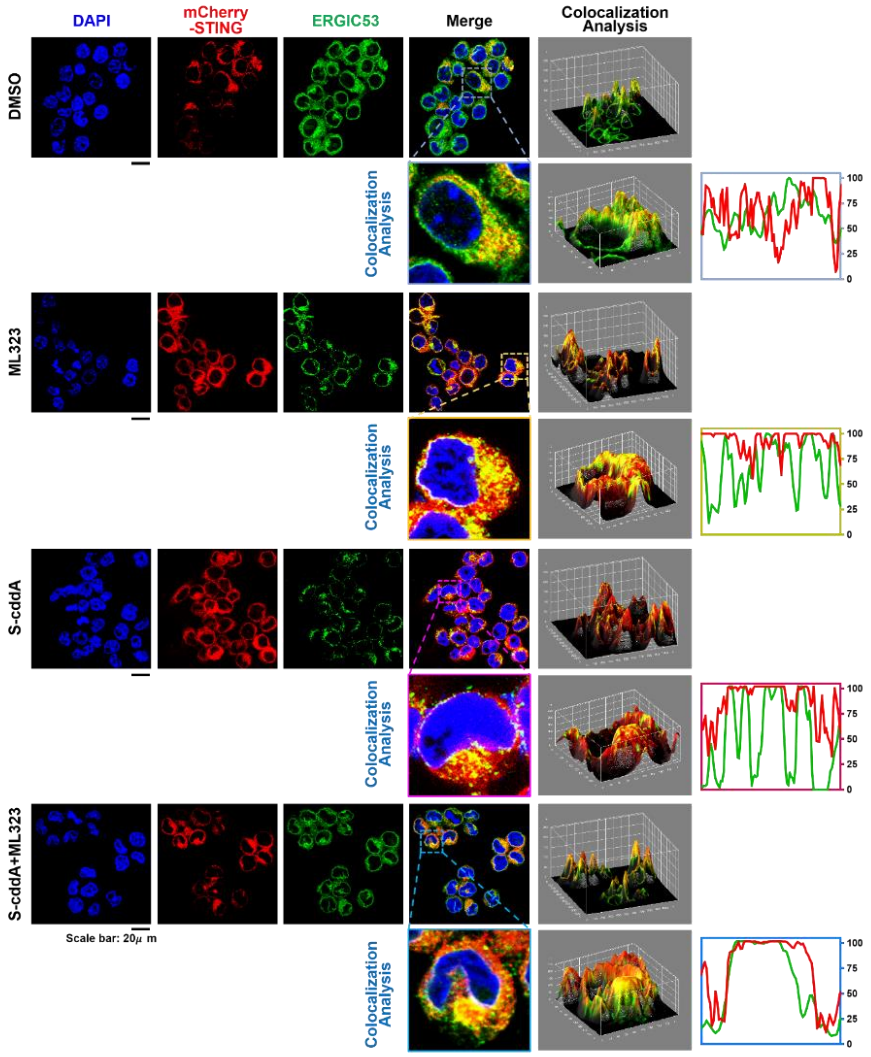

Figure S13. ML323 promotes S-cddA mediated STING trafficking from ER to ERGIC/Golgi. IF analysis of distribution and colocalization analysis of mCherry-STING and ERGIC53 in L929-mCherry-STING cells treated by S-cddA (100 nM) combined with ML323 (5 μM).

Figure S14

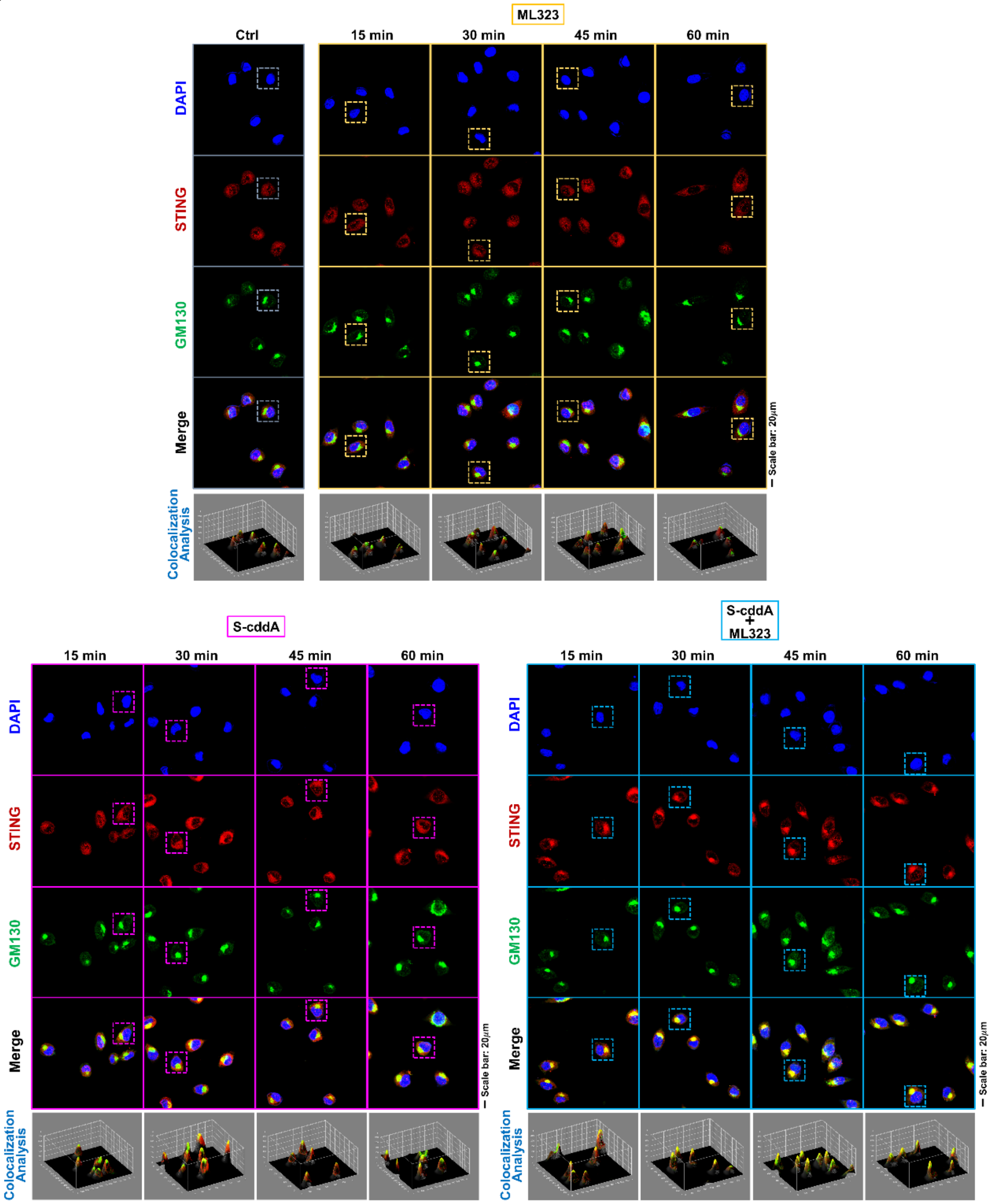

Figure S14. ML323 promotes S-cddA mediated STING transport to the Golgi. IF analysis of distribution and colocalization analysis of STING and GM130 in L929 cells treated by S-cddA (300 nM) combined with ML323 (5 μM) for the indicated times, **related to Fig. 4C.**

Figure S15

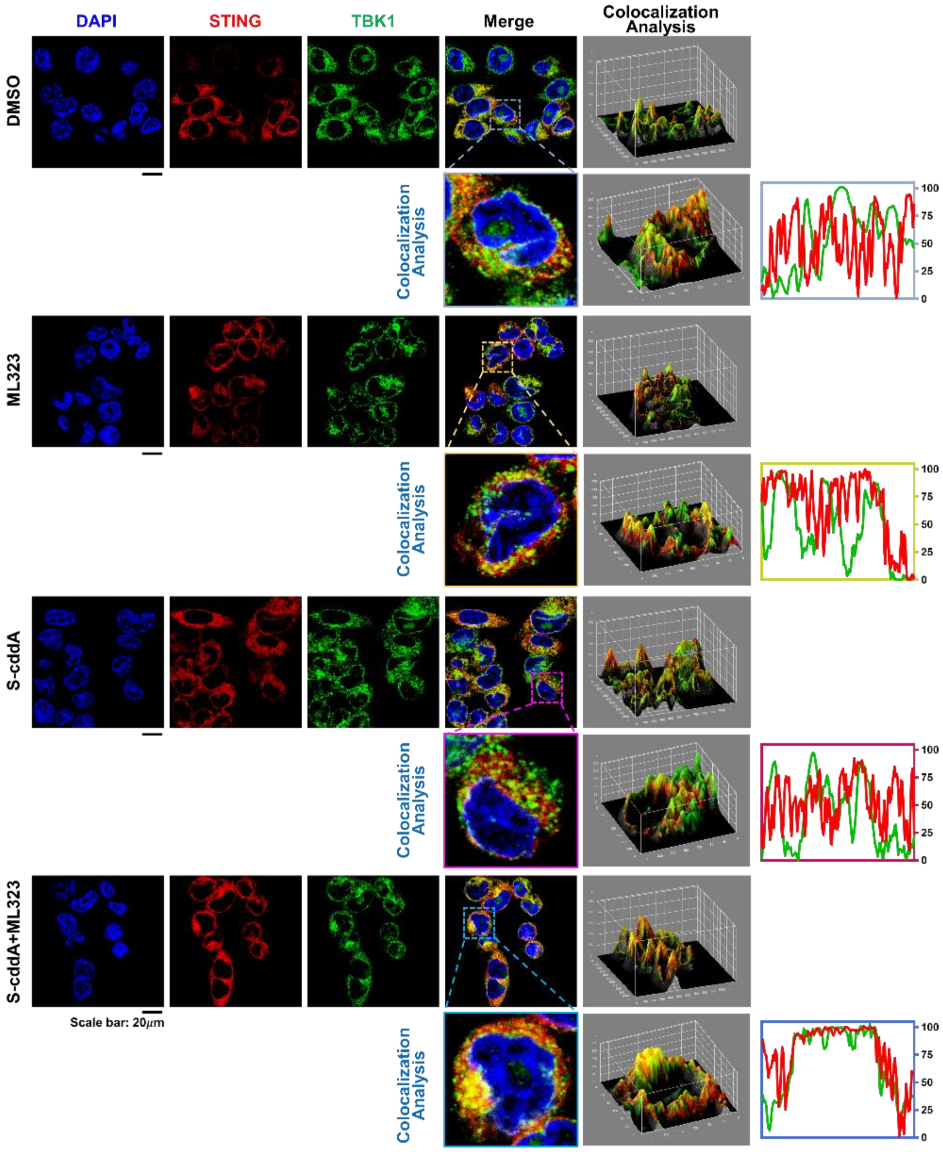

Figure S15. **ML323 promotes S-cddA mediated the interaction between STING and TBK1.** IF analysis of distribution and colocalization analysis of STING and TBK1 in L929 cells treated by S-cddA (100 nM) combined with ML323 (5 μM).

Figure S16

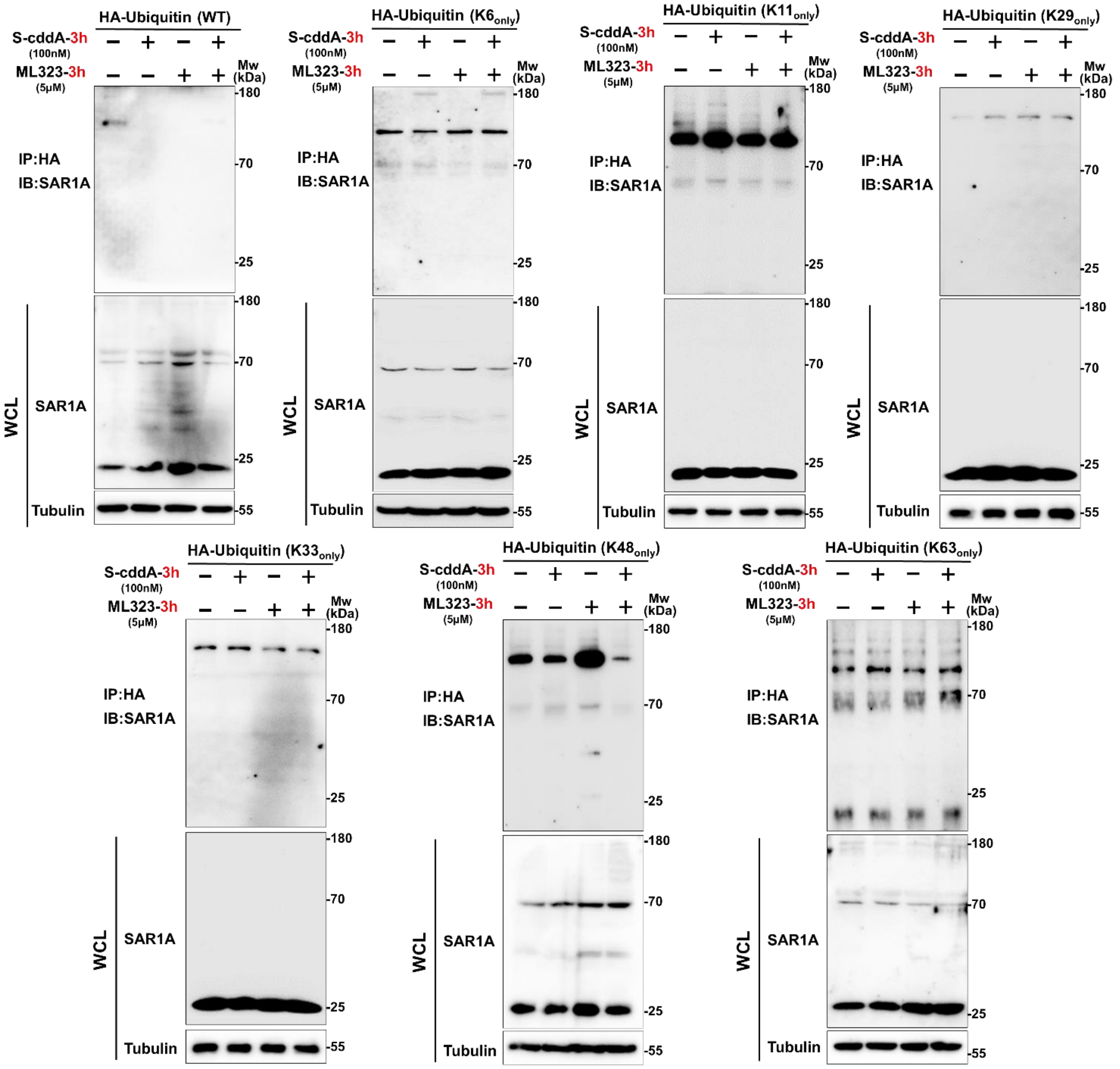

Figure S16. The oligo-ubiquitinated status of SAR1A at various K linkage forms when USP1 was suppressed, related to Figure 5C. Co-IP/WB assay in L929-HA-UB-(WT/K6/K11/K29/K33/K48/K63 only) cells, revealed that the combined treatment with S-cddA and ML323 resulted oligo-ubiquitinated form of SAR1A have not been enhanced. All data were obtained from biological replicates conducted more than 3 times.

Figure S17

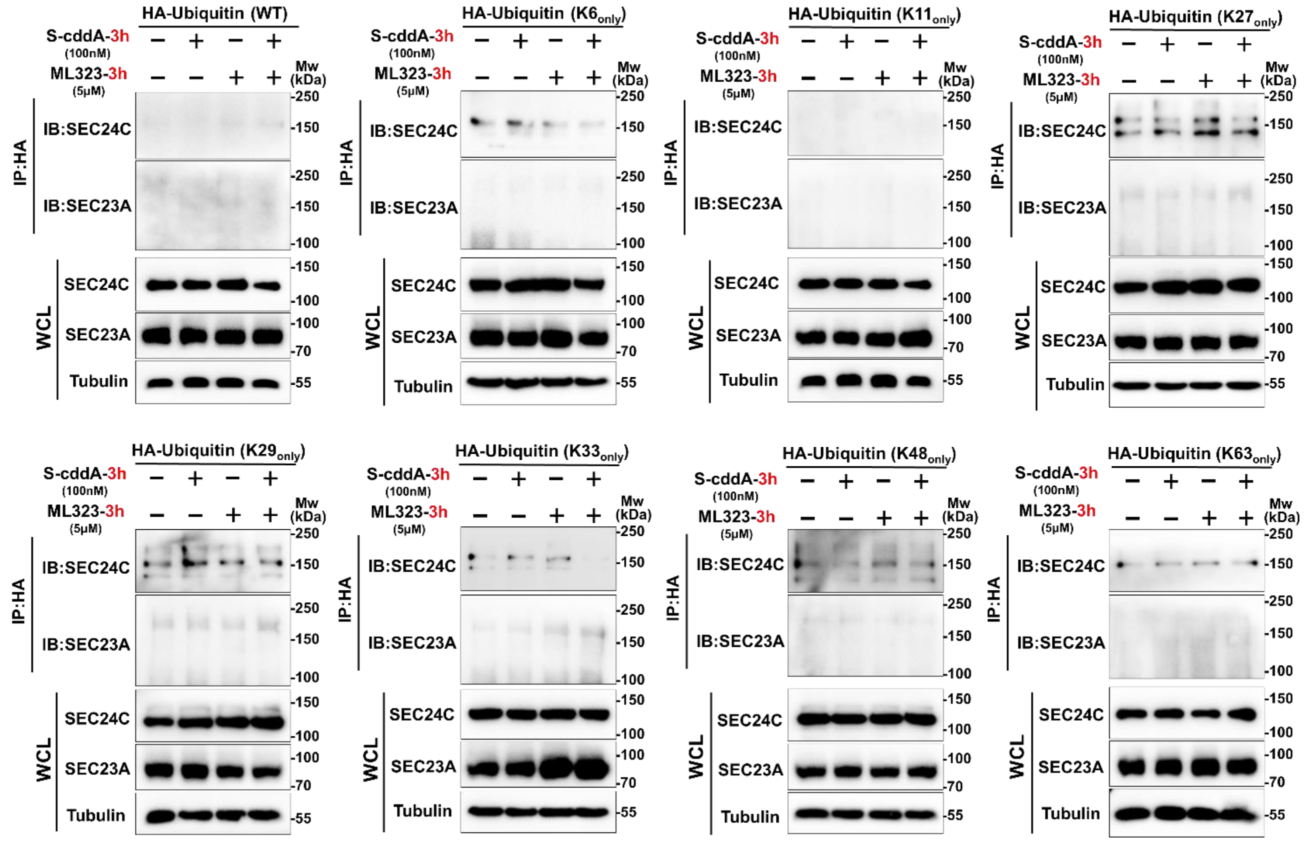

Figure S17. The oligo-ubiquitinated status of SEC24C and SEC23A at various K linkage forms when USP1 was suppressed, related to Figure 5F. Co-IP/WB analysis of L929-HA-ubiquitin (WT/K6/K11/K27/K29/K33/K48/K63 only) cells treated by S-cddA combined with ML323, revealed that the combined treatment with S-cddA and ML323 resulted oligo-ubiquitinated form of SEC24C or SEC23A have not been enhanced. All data were obtained from biological replicates conducted more than 3 times.

Figure S18

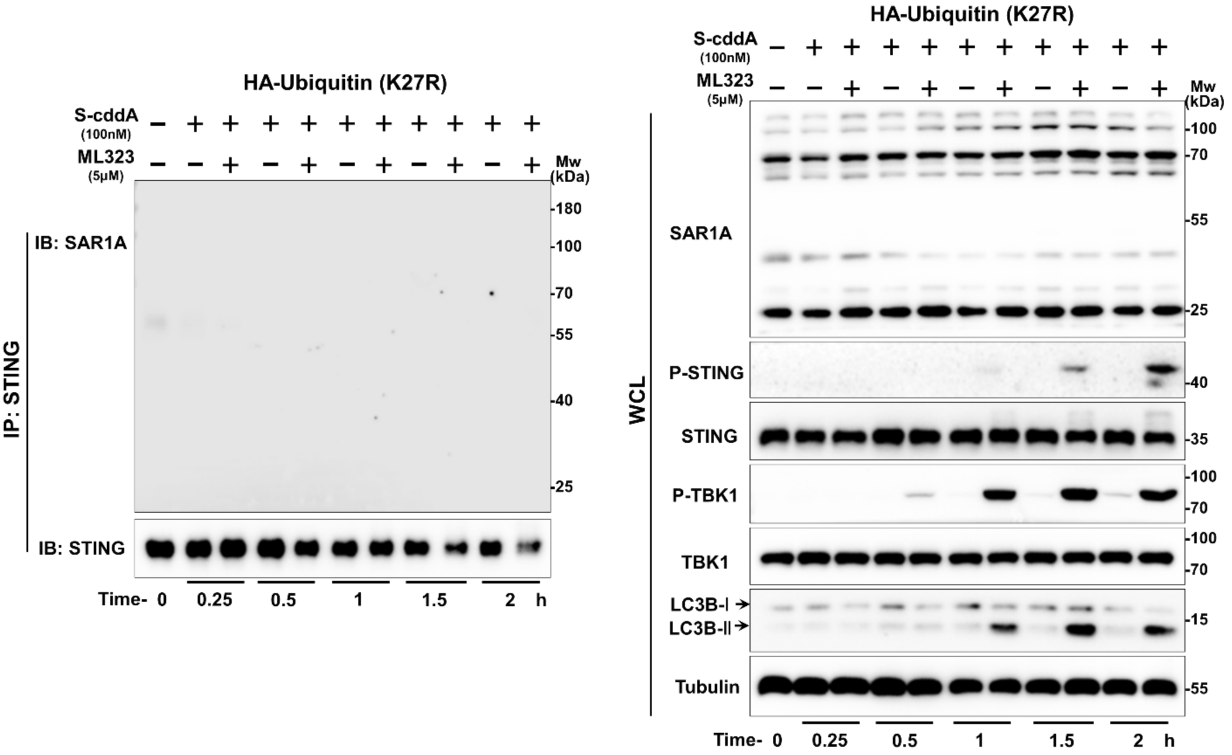

Figure S18. The impact of K27R mutated ubiquitin on the interaction between STING and oligo-ubiquitination state of SAR1A when USP1 is inhibited. Co-IP/WB analysis of L929-HA-ubiquitin (K27R only) cells treated by S-cddA combined with ML323 for 0.25, 0.5, 1.0, 1.5, 2.0 hours. All data were obtained from biological replicates conducted more than 3 times.

Figure S19

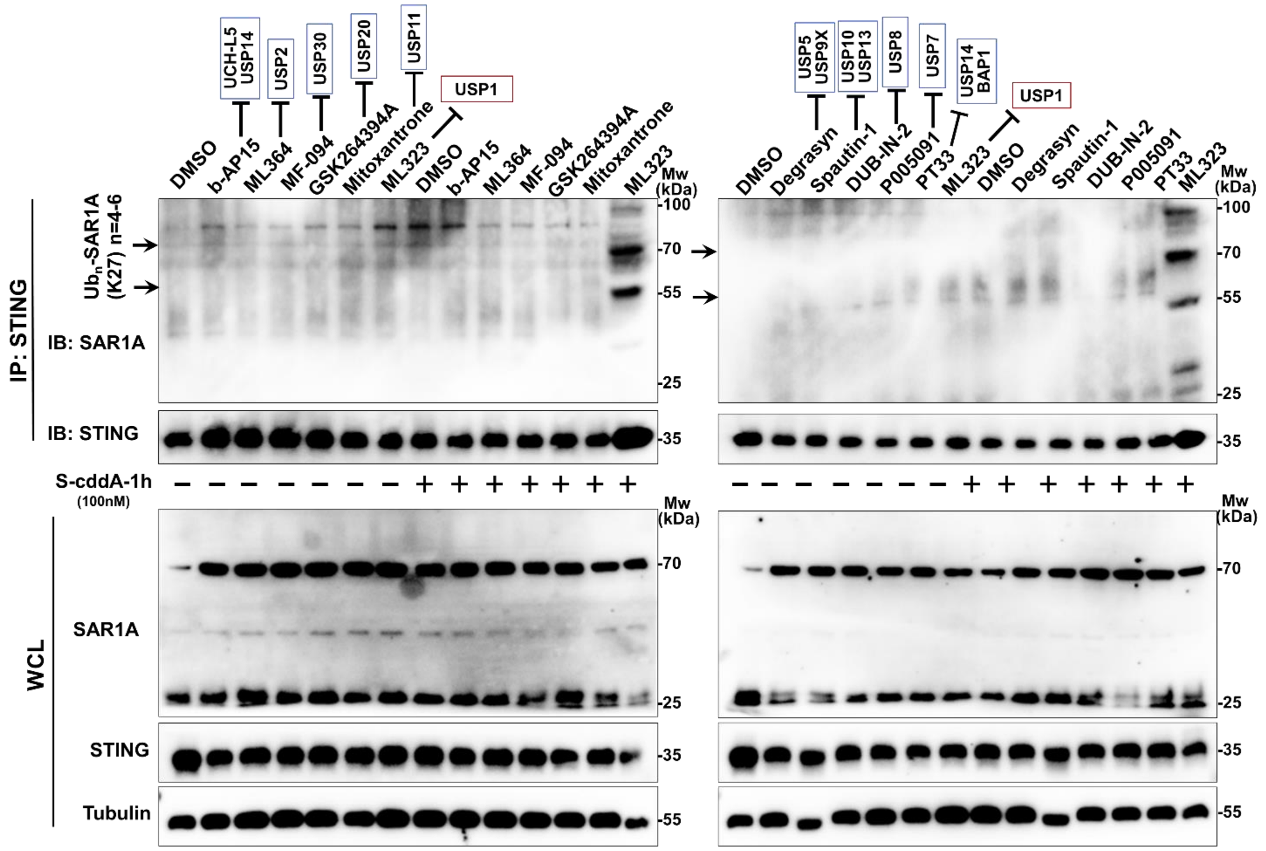

Figure S19. Screening DUB inhibitors to promote the accumulation of the K27-linked oligo-ubiquitination state of SAR1A. Co-IP/WB analysis of L929-HA-ubiquitin (K27 only) cells treated by S-cddA combined with DUB inhibitors. All data were obtained from biological replicates conducted more than 3 times.

Figure S20

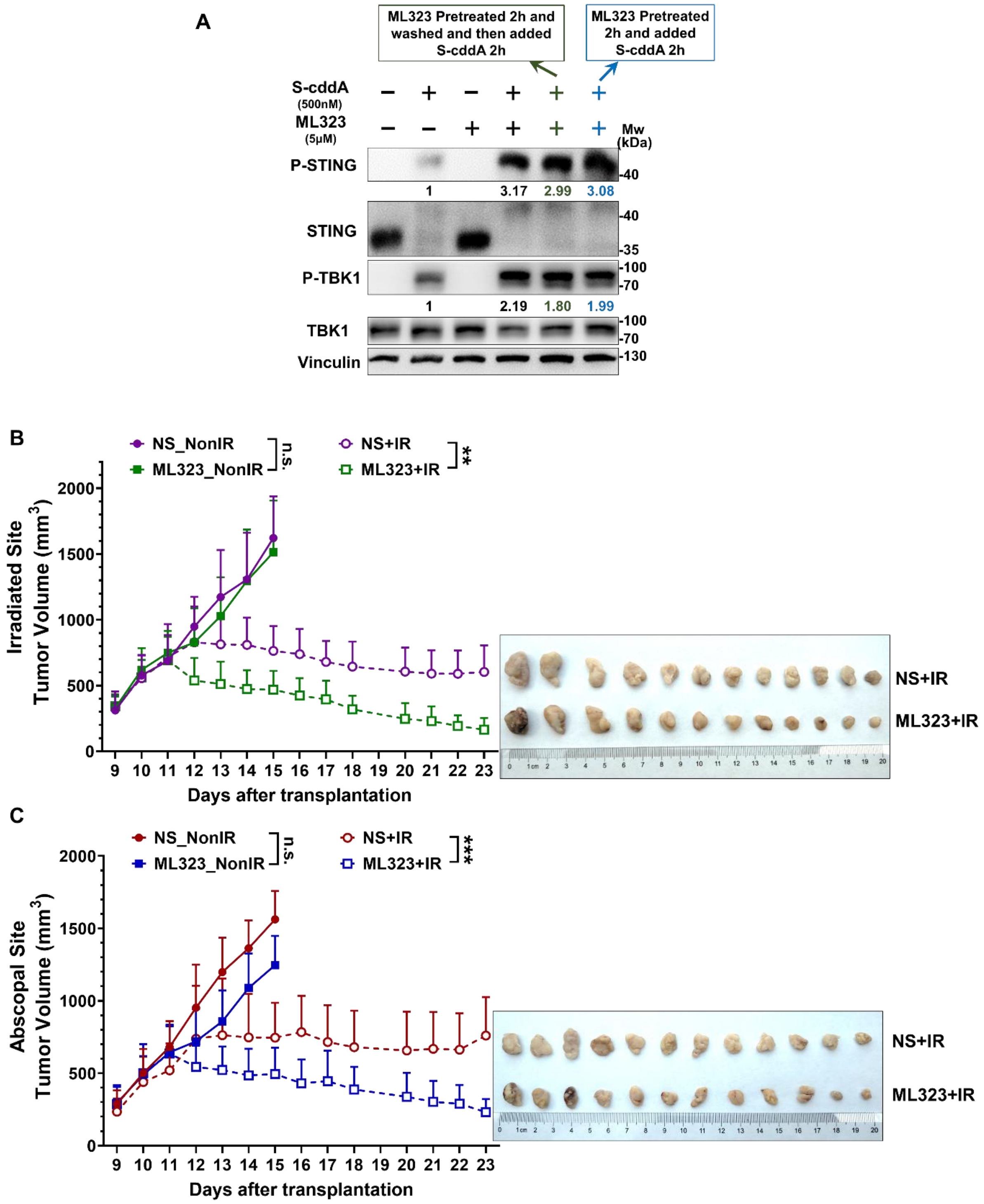

Figure S20. The effect of ML323 on anti-tumor and abscopal effect mediated by RT. (**A**) In L929 cells, by co incubating ML323 with S-cddA for 2 hours, pre incubating ML323 for 2 hours followed with washing ML323 and then adding S-cddA for 2 hours, and pre incubating ML323 with S-cddA for

2 hours, protein levels of p-STING and p-TBK1 were detected by WB assay. All data were obtained from biological replicates conducted more than 3 times. (**B**-**C**) Starting from the 9th day of tumor implantation, the tumor volume of BalB/c mice (**B:** irradiated site; **C:** abscopal site) was recorded daily. On the 15th day, mice in the blank control group and ML323 group were euthanized. On the 23rd day, mice in the radiotherapy group and the radiotherapy combined with ML323 group were euthanized, **related to Figure 6B.** Statistical significance was determined by one-way ANOVA, (N.S., no significance; **, P<0.01; ***, P<0.001).

Figure S21

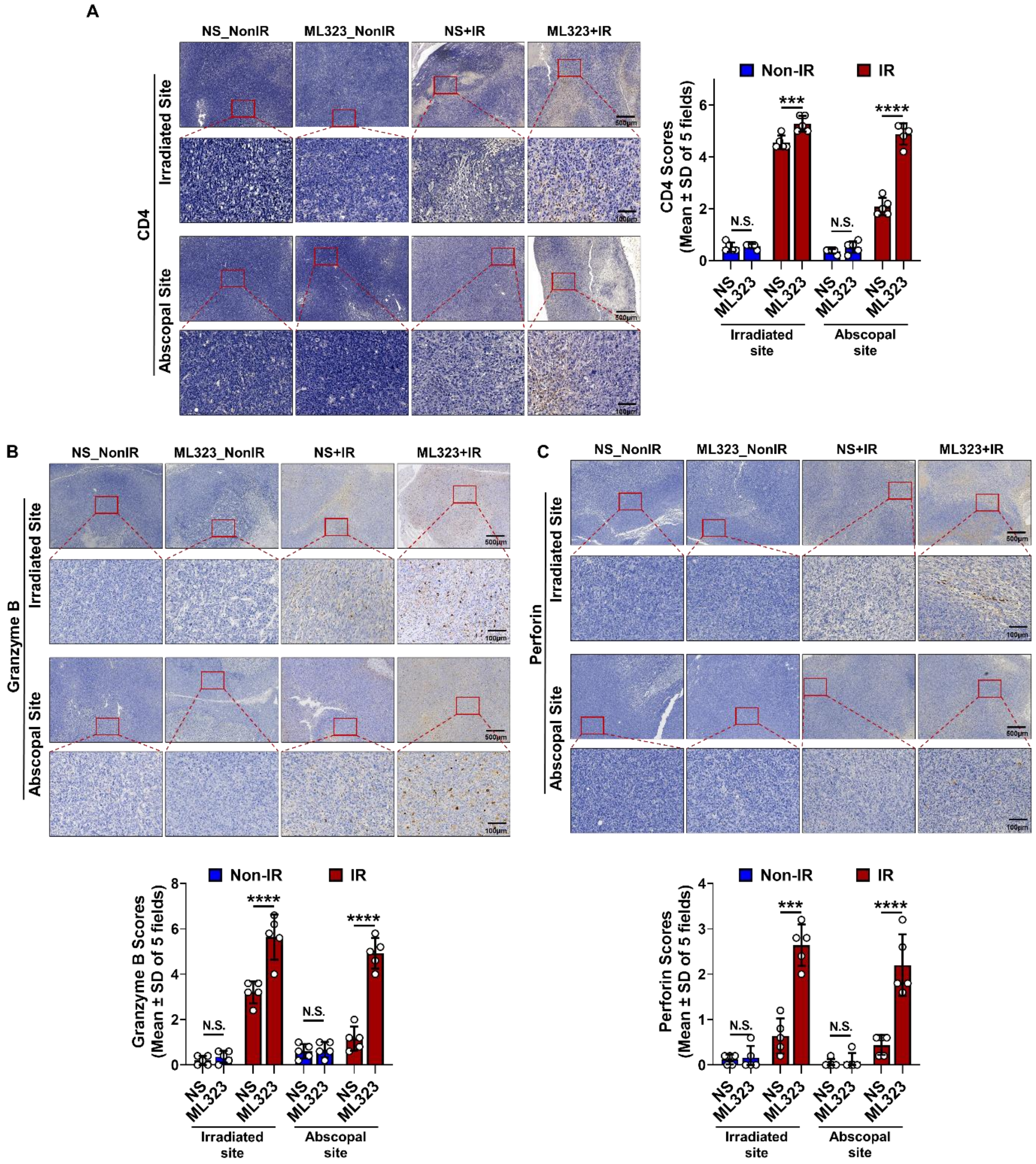

Figure S21. The impact of ML323 on immune infiltration in the irradiated and abscopal side tumors following RT. The tumors were analyzed by IHC to measure the level of $CD4^+$ T cells (**A**), Granzyme B (**B**) and Perforin infiltration (**C**), **related to Figure 6D**. Statistical significance was determined by two-way ANOVA, (N.S., no significance; ***, P<0.001; ****, P<0.0001).

Figure S22

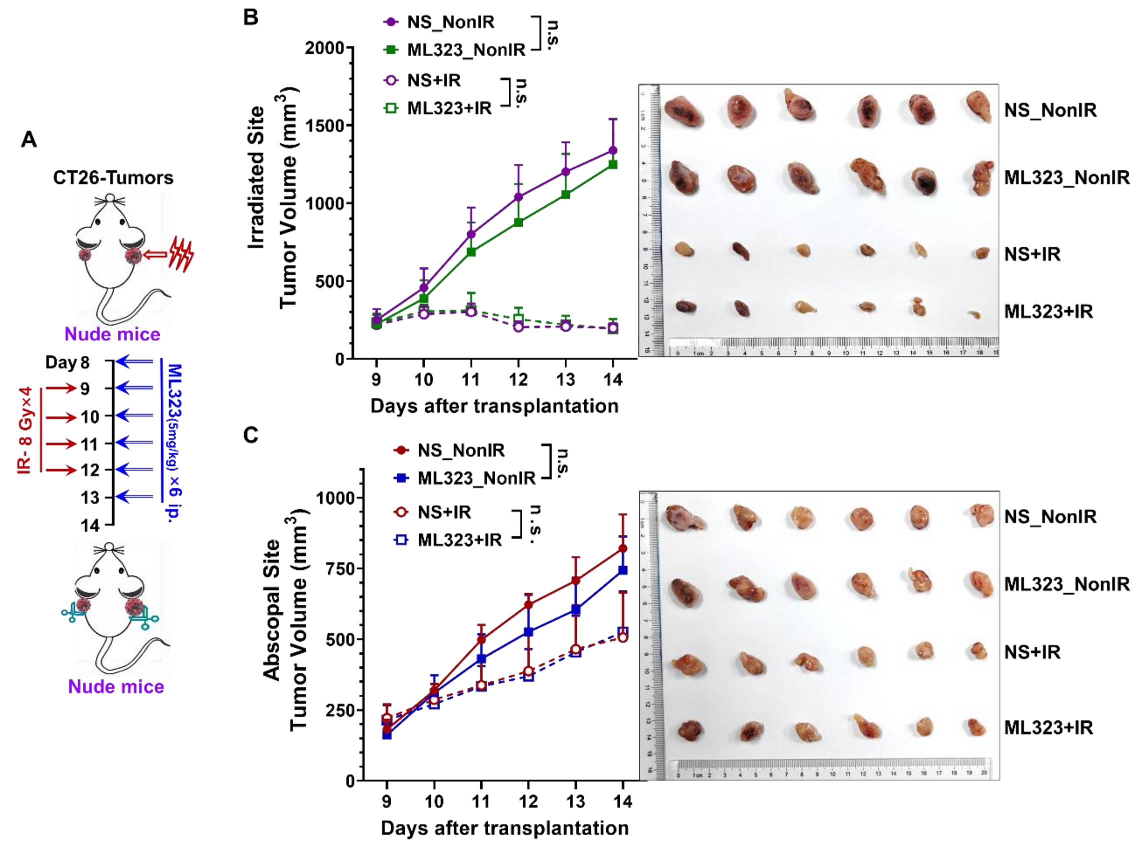

Figure S22. ML323 does not exhibit a radiosensitizing effect or abscopal effect in nude mice. (**A**) Schematic diagram of the experimental design demonstrating the abscopal effect of RT, involving tumor-bearing mice (CT26 tumors implanted in bilateral axillae) treated with IR and ML323. (**B-C**) Starting from the 9th day of tumor implantation, the tumor volume of nude mice (**B:** irradiated site; **C:** abscopal site) was recorded daily. On the 14th day, mice were euthanized. Statistical significance was determined by one-way ANOVA, (n.s., no significance).

Figure S23

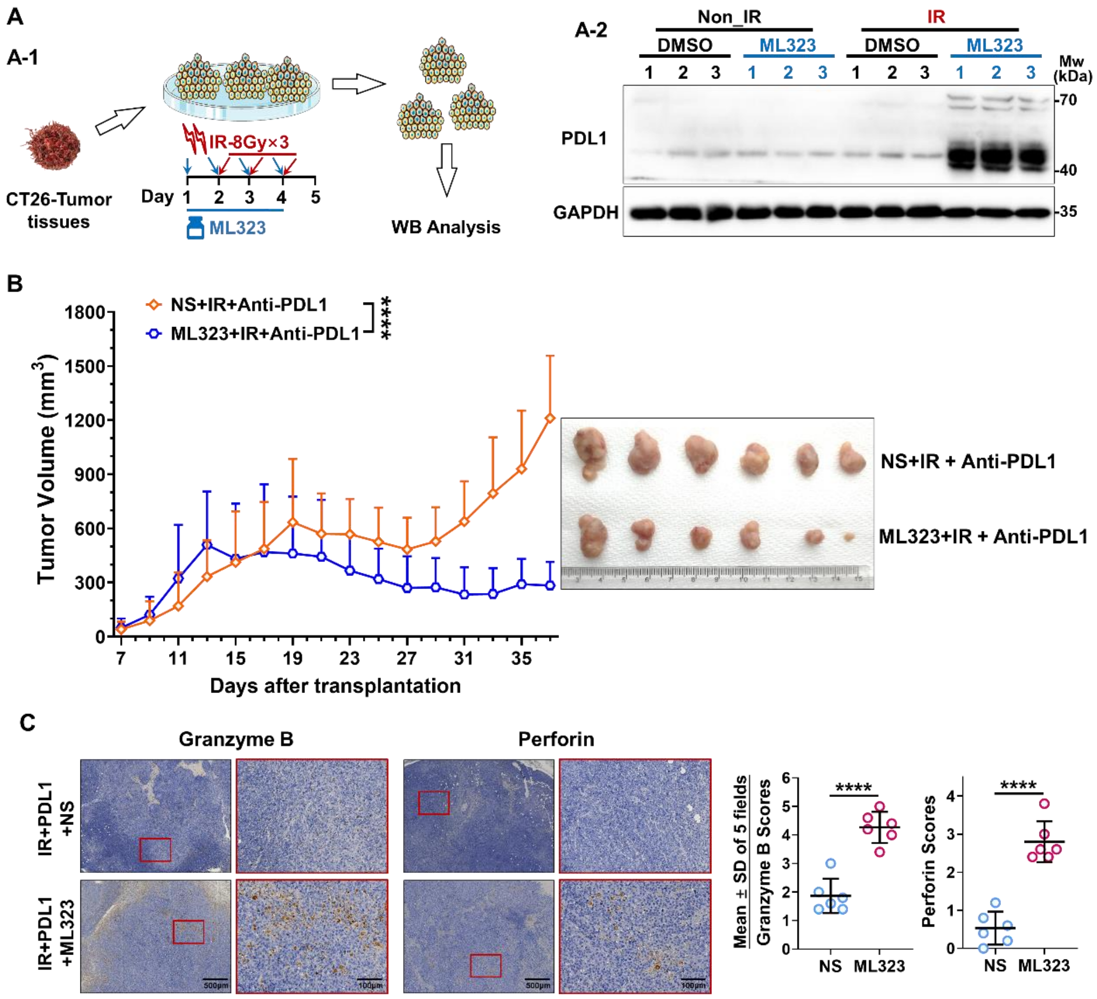

Figure S23. ML323 enhances the effect of RT + immunotherapy. (**A**) Effects of ML323 on PDL1 expression levels of tumor tissues following RT. (**A-1**) Schematic diagram; (**A-2**) PDL1 expression levels in the indicated treatment group of mouse tumor tissues were detected by WB assay. (**B**) BalB/c mice were divided into radiotherapy combined with PD-L1 group, and ML323 + RT combined with PD-L1 group. Starting from the 7th day of tumor implantation, the tumor volume was recorded daily. Two groups of mice were euthanized on the 37th day, **related to Figure 6F**. (**C**) The tumors were analyzed by IHC to measure the level of granzyme B and perforin infiltration. Statistical significance of **B** was determined by two-way ANOVA, Statistical significance of **C** was determined by one-way ANOVA (****, P<0.0001).

Figure S24

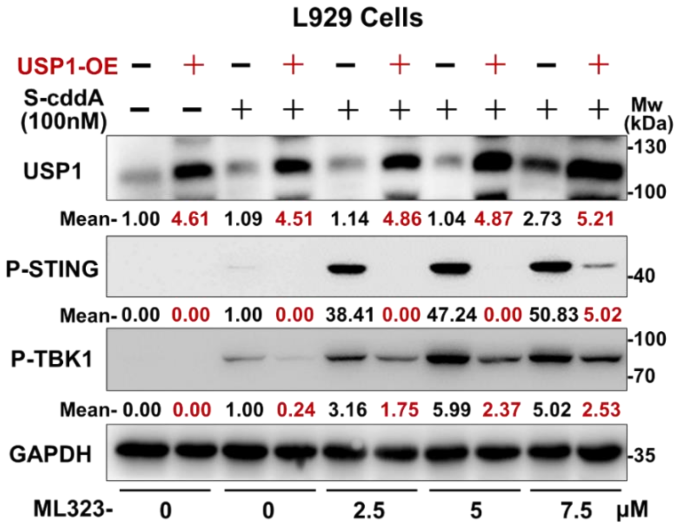

Figure S24. The extent of STING activation is influenced by the expression level of USP1. In USP1-WT/OE L929 cells, the protein levels of P-STING and P-TBK1 were detected by WB analysis after co incubation with S-cddA and the indicated concentrations of ML323. All data were obtained from biological replicates conducted more than 3 times.

Figure S25

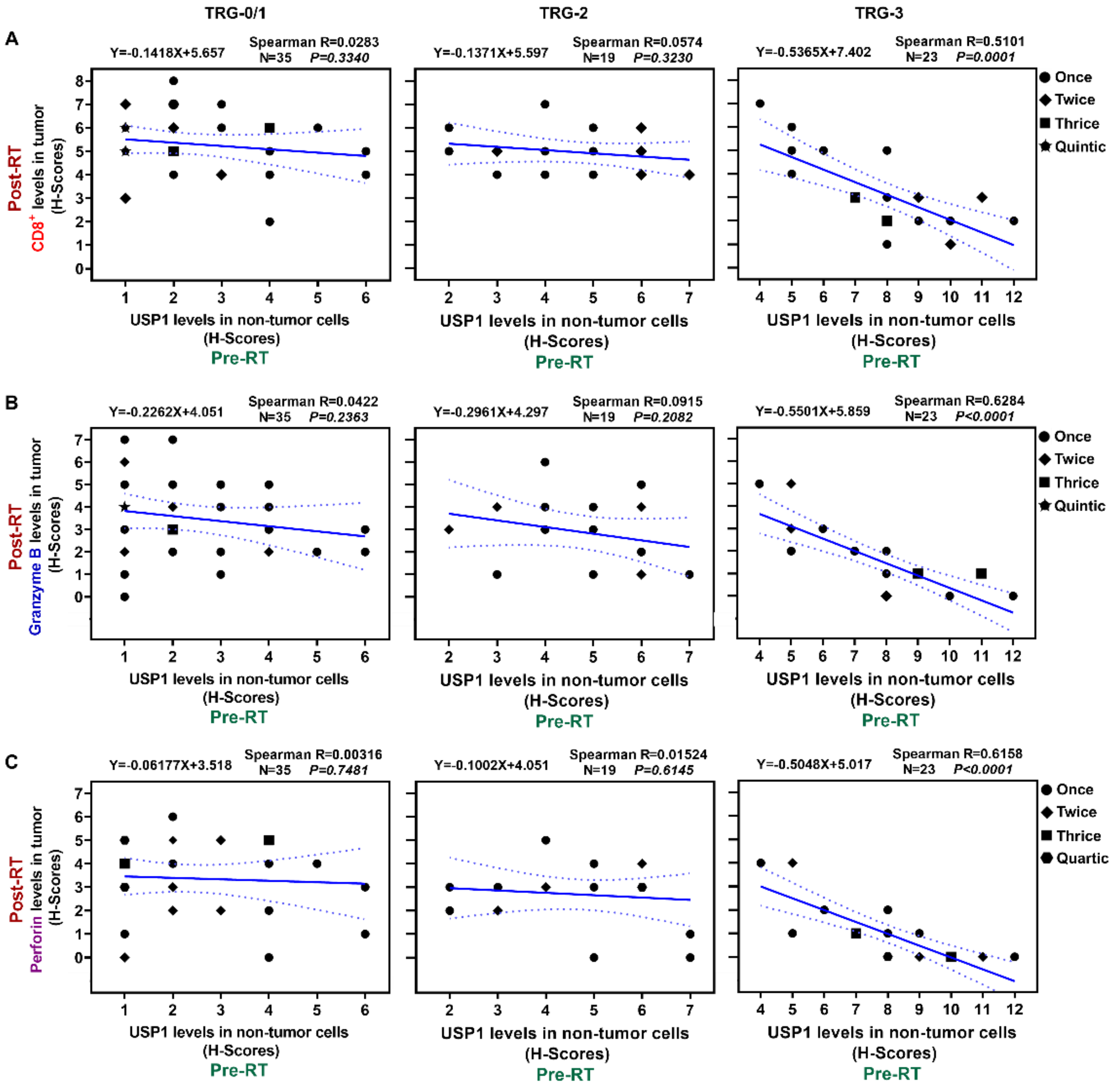

Figure S25.The correlation between the expression level of USP1 in non-tumor cells before RT and the infiltration levels of CD8^{+} T cells, granzyme B and perforin in tumor cells following RT among patients with different TRG grades. Spearman analysis of TRG0/1 and TRG2 patients between USP1 levels in pre-RT non-tumor cells and CD8^{+} T cells (**A**), Granzyme B (**B**) and Perforin (**C**) infiltration levels in post-RT tumor cells showed no correlation (R<0.3). In TRG3 patients, the result showed moderate correlation (0.5≤R<0.8).

Figure S26

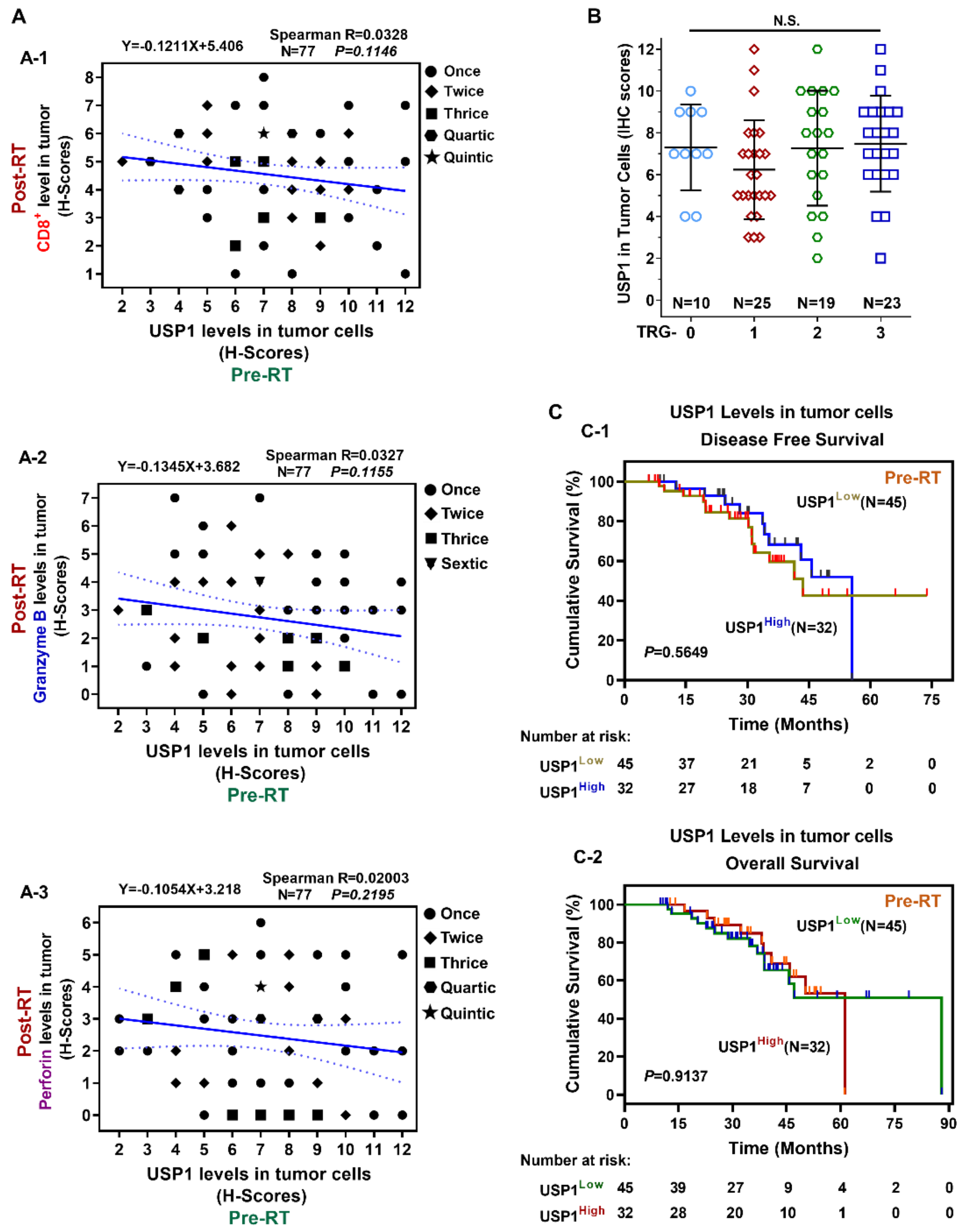

Figure S26. The correlation between the expression level of USP1 in tumor cells before RT and the survival rate of patients following RT. (**A1-A3**) Spearman analysis of tumor tissues between USP1 levels in tumor cells and $CD8^+$ T cell, granzyme B and perforin infiltration levels in tumor showed no correlation (R<0.3). (**B**) IHC analysis of the differences in USP1 expression levels in tumor cells among patients with TRG grades 0, 1, 2, and 3. (**C**) Cumulative survival analysis of patients showed no correlation between USP1 levels in tumor cells and DFS or OS (P>0.05). Statistical significance was determined by one-way ANOVA, (N.S., no significance).

Supplementary Tables

Table S1: Correlation between levels of USP1 in non-tumor cells and clinicopathological parameters for pre-RT samples

Variables	No. of patients (%)	USP1 levels		P value
		Low (n; %)	High (n; %)	
**Gender**				***0.330***
Male	55 (71.4)	41 (53.2)	14 (18.2)	
Female	22 (28.6)	16 (20.8)	6 (7.8)	
**Age**				***0.656***
≤62	56 (72.7)	29 (37.7)	27 (35.0)	
>62	21 (27.3)	9 (11.7)	12 (15.6)	
**Pathologic grade**				***0.251***
I	6 (7.8)	5 (6.5)	1 (1.3)	
II	58 (75.3)	43 (55.8)	15 (19.5)	
III	13 (16.9)	8 (10.4)	5 (6.5)	
**cT**				***0.521***
T2	1 (1.3)	1 (1.3)	0 (0)	
T3	47 (61.0)	33 (42.8)	14 (18.2)	
T4	29 (37.7)	23 (29.9)	6 (7.8)	
**cN**				***0.100***
N0	7 (9.1)	6 (7.8)	1 (1.3)	
N1	29 (37.7)	24 (31.2)	5 (6.5)	
N2	41 (53.2)	27 (35.0)	14 (18.2)	
**CEA (ng/mL)**				***0.993***
<5	44 (57.1)	32 (41.5)	12 (15.6)	
≥5	33 (42.9)	25 (32.5)	8 (10.4)	
**CA19-9(Ku/l)**				***0.845***
≤35	59 (76.6)	44 (57.1)	15 (19.5)	
>35	18 (23.4)	13 (16.9)	5 (6.5)	
**Adjuvant chemotherapy**				
No	24 (31.2)	17 (22.1)	7 (9.1)	***0.672***
Yes	53 (68.8)	40 (51.9)	13 (16.9)	
**Surgical procedure**				***0.095***
Dixon	59 (76.6)	41 (53.2)	18 (23.4)	
Miles	16 (20.8)	14 (18.2)	2 (2.6)	
Hartmann	2 (2.6)	2 (2.6)	0 (0)	
**TRG**				***0.000****
0	10 (13.0)	10 (13.0)	0 (0)	
1	25 (32.4)	25 (32.4)	0 (0)	
2	19 (24.7)	17 (22.1)	2 (2.6)	
3	23 (29.9)	5 (6.5)	18 (23.4)	
**Status**				***0.000****
Alive	53 (68.8)	48 (62.3)	5 (6.5)	
Dead	24 (31.2)	9 (11.7)	15 (19.5)	

cT: clinical T stage; cN: clinical N stage

Table S2: Correlation between levels of USP1 in tumor cells and clinicopathological parameters for pre-RT samples

Variables	No. of patients (%)	USP1 levels		P value
		Low (n; %)	High (n; %)	
**Gender**				***0.616***
Male	55 (71.4)	33 (42.8)	22 (28.6)	
Female	22 (28.6)	12 (15.6)	10 (13.0)	
**Age**				***0.414***
≤62	38 (49.4)	24 (31.2)	14 (18.2)	
>62	39 (50.6)	21 (27.2)	18 (23.4)	
**Pathologic grade**				***0.966***
I	6 (7.8)	4 (5.2)	2 (2.6)	
II	58 (75.3)	33 (42.8)	25 (32.5)	
III	13 (16.9)	8 (10.4)	5 (6.5)	
**cT**				***0.775***
T2	1 (1.3)	0 (0)	1 (1.3)	
T3	47 (61.0)	28 (36.3)	19 (24.7)	
T4	29 (37.7)	17 (22.1)	12 (15.6)	
**cN**				***0.015****
N0	7 (9.1)	5 (6.5)	2 (2.6)	
N1	29 (37.7)	22 (28.6)	7 (9.1)	
N2	41 (53.2)	18 (23.3)	23 (29.9)	
**CEA (ng/mL)**				***0.292***
<5	44 (57.1)	28 (36.3)	16 (20.8)	
≥5	33 (42.9)	17 (22.1)	16 (20.8)	
**CA19-9(Ku/L)**				***0.780***
≤35	59 (76.6)	35 (45.4)	24 (31.2)	
>35	18 (23.4)	10 (13.0)	8 (10.4)	
**Adjuvant chemotherapy**				***0.331***
No	24 (31.2)	16 (20.8)	8 (10.4)	
Yes	53 (68.8)	29 (37.7)	24 (31.1)	
**Surgical procedure**				***0.886***
Dixon	59 (76.6)	34 (44.1)	25 (32.5)	
Miles	16 (20.8)	10 (13.0)	6 (7.8)	
Hartmann	2 (2.6)	1 (1.3)	1 (1.3)	
**TRG**				***0.112***
0	10 (13.0)	6 (7.8)	4 (5.2)	
1	25 (32.4)	19 (24.6)	6 (7.8)	
2	19 (24.7)	9 (11.7)	10 (13.0)	
3	23 (29.9)	11 (14.3)	12 (15.6)	
**Status**				***0.990***
Alive	53 (68.8)	31 (40.2)	22 (28.6)	
Dead	24 (31.2)	14 (18.2)	10 (13.0)	

cT: clinical T stage; cN: clinical N stage

Table S3: Information of reagents and resource

REAGENT or RESOURCE	SOURCE	IDENTIFIER
**Antibodies**		
Rabbit anti-STING	Cell Signaling Technology	Cat#13647; RRID: AB_2732796
Rabbit anti-Phospho-STING (Ser365)	Cell Signaling Technology	Cat#72971; RRID: AB_2799831
Rabbit anti-TBK1/Nak	Cell Signaling Technology	Cat#3504; RRID: AB_2255663
Rabbit anti-p-TBK1/p-Nak (Ser172)	Cell Signaling Technology	Cat#5483; RRID: AB_10693472
Rabbit anti-LC3A/B	Cell Signaling Technology	Cat#2275; RRID: AB_915950
Rabbit anti-ERp72	Cell Signaling Technology	Cat#5033; RRID: AB_2160984
Rabbit anti-USP1	Cell Signaling Technology	Cat#8033; RRID: AB_10858879
Rabbit anti-PI3 Kinase Class III (D9A5) (VPS34)	Cell Signaling Technology	Cat#4263; RRID: AB_2299765
Rabbit anti-SEC24C	Cell Signaling Technology	Cat#8513; RRID: AB_11141829
Rabbit anti-GAPDH	Cell Signaling Technology	Cat#2188; RRID: AB_561053
Rabbit anti-Vinculin	Cell Signaling Technology	Cat#18799; RRID: AB_2714181
Rabbit anti-β-Tubulin	Cell Signaling Technology	Cat#15115; RRID: AB_2798712
Rabbit anti-Phospho-mTOR (Ser2448)	Cell Signaling Technology	Cat#5536; RRID: AB_10691552
Rabbit anti-SAR1A	Proteintech	Cat#22291-1-AP; RRID: AB_2879062
Rabbit Anti-ERGIC-53 / p58	Sigma-Aldrich	Cat# E1031; RRID: AB_532237
Rabbit anti-HA	Proteintech	Cat#22291-1-AP; RRID: AB_11042321
Rabbit anti-Myc	Proteintech	Cat#16286-1-AP; RRID: AB_11182162
P62, SQSTM1 Recombinant antibody	Proteintech	Cat#80294-1-RR; RRID: AB_2918883
Phospho-P62, SQSTM1 (Ser349) Polyclonal antibody	Proteintech	Cat#29503-1-AP; RRID: AB_2923592
Rabbit anti SEC23A	ABclonal	Cat#A12101; RRID: AB_2758996
Mouse anti-GM130	BD Transduction Laboratories™	Cat#610822; RRID: AB_398141
Mouse anti-dsDNA	Abcam	Cat#ab27156; RRID: AB_470907
Anti-Perforin (CB5.4)	Abcam	Cat#ab16074; RRID: AB_302236
Anti-Perforin (5B10)	Abcam	Cat#ab89821; RRID: AB_2042606
Anti-Granzyme B (EPR22645-206)	Abcam	Cat#ab255598; RRID: AB_2860567
Mouse-anti-STING	Proteintech	Cat#66680-1-Ig; RRID:

		AB_2882034
Mouse-anti-HA	Proteintech	Cat#66006-2-Ig; RRID: AB_2881490
Mouse-anti-Myc	Proteintech	Cat#60003-2-Ig; RRID: AB_2734122
USP1 Polyclonal antibody	Proteintech	Cat#14346-1-AP; RRID: AB_2214314
CD8a Monoclonal antibody	Proteintech	Cat#66868-1-Ig; RRID: AB_2882205
Recombinant Anti-CD4 antibody	Servicebio	Cat#GB15064; RRID: AB_3095557
Recombinant Anti-CD8 alpha	Servicebio	Cat#GB15068; RRID: AB_3246431
Anti -CD11c	Servicebio	Cat#GB11059; RRID: AB_2905514
HRP-conjugated Recombinant Rabbit Anti-Mouse IgG Kappa Light Chain	Proteintech	Cat#SA00001-19; RRID: AB_2935617
Mouse Anti-Rabbit IgG Light Chain Specific, HRP conjugate	Proteintech	Cat#SA00001-7L; RRID: AB_2890988
Goat Anti-Mouse IgG H&L (Alexa Fluor® 488)	Abcam	Cat#ab150113; RRID: AB_2576208
Goat Anti-Rabbit IgG H&L (Alexa Fluor® 647)	Abcam	Cat#ab150079; RRID: AB_2722623
In Vivo MAb anti-mouse PD-L1	Bioxcell	Cat#BE0101; RRID: AB_10949073
Fixable Viability Stain 700	BD Transduction Laboratories™	Cat#564997; RRID: AB_2869637
PE-Cy7 Hamster Anti-Mouse CD3e (145-2C11)	BD Transduction Laboratories™	Cat#610822; RRID: AB_394460
Rat Anti-Mouse CD4 (RM4-5)	BD Transduction Laboratories™	Cat#566407; RRID: AB_2744427
FITC Rat Anti-Mouse CD8a (53-6. 7)	BD Transduction Laboratories™	Cat#553030; RRID: AB_394568
Rat Anti-Mouse CD45	BD Transduction Laboratories™	Cat#567111; RRID: AB_2870024
Hanster Anti-Mouse CD11c	BD Transduction Laboratories™	Cat#759736; RRID: AB_3674667
Rat Anti-Mouse Ly-6C	BD Transduction Laboratories™	Cat#771169; RRID: AB_3674668
**Chemicals and Reagents**		
DMSO	Sigma-Aldrich	Cat#D4540
LDN57444	TargetMol, USA	Cat#T1924, CAS 668467-91-2
B-AP15	TargetMol, USA	Cat#T1932, CAS 1009817-63-3
ML323	TargetMol, USA	Cat#T1757, CAS 1572414-83-5
ML364	TargetMol, USA	Cat#T3555, CAS 1991986-30-1
XL177A	TargetMol, USA	Cat#T9122, CAS 2417089-74-6
DUB-IN-2	TargetMol, USA	Cat# T11111, CAS 924296-19-5
Mitoxantrone	TargetMol, USA	Cat# T6588, CAS 65271-80-9
GSK2643943A	TargetMol, USA	Cat#T11485, CAS 2449301-27-1
P005091	TargetMol, USA	Cat#T6925, CAS 882257-11-6
Degrasyn	TargetMol, USA	Cat#T6300, CAS 856243-80-6
Spautin1	TargetMol, USA	Cat#T1937, CAS 1262888-28-7
USP15-IN-1	TargetMol, USA	Cat#T61575, CAS 2260826-16-0
GSK2643943A	TargetMol, USA	Cat#T11485，CAS 2449301-27-1

USP28-IN-3	TargetMol, USA	Cat#T74793, CAS 2931509-14-5
USP22-IN-1	TargetMol, USA	Cat#T73151，CAS 309735-96-4
MF-094	TargetMol, USA	Cat#T12024, CAS 2241025-68-1
OTUB1/USP8-IN-1	TargetMol, USA	Cat#T72784, CAS 2858800-98-1
USP7/47-IN-1	TargetMol, USA	Cat#T13268, CAS 1381291-36-6
SJB2-043	TargetMol, USA	Cat#T7678, CAS 63388-44-3
H151	TargetMol, USA	Cat#T5674, CAS 941987-60-6
2',3'-cGAMP sodium	TargetMol, USA	Cat#T10065L, CAS 2734858-36-5
DMXAA	Ambeed	Cat#A140665, CAS 117570-53-3
Brefeldin A (BFA)	Med Chem Express	Cat#HY-16592, CAS 20350-15-6
PT33	Autonomous synthesis	N/A
Sate-cddA	Autonomous synthesis	N/A
DMEM, high glucose, pyruvate	Corning	Cat#10-013-CV
RPMI 1640	Corning	Cat#10-040-CVRC
Fetal Bovine Serum (Characterized)	Bio-Channel	Cat#BC-SE-FBS07
Penicillin-streptomycin	New Cell & Molecular Biotech	Cat# C100C5
rmGM-CSF	PeproTech	Cat# no. 315–03-20
L-glutamine	MedChemExpress	Cat#HY-N0390, CAS 56-85-9
2-mercaptoethanol	MedChemExpress	Cat#HY-N0390, CAS 60-24-2
Red cell lysing	Solarbio	Cat# R1010
Newborn Calf Serum	Gibco	Cat# 16010159
VeZol Reagent	Vazyme Biotech Co.,Ltd	Cat# R411-01/02
HiScript II Q RT SuperMix for qPCR (+gDNA wiper)	Vazyme Biotech Co.,Ltd	Cat# R223-01
ChamQ SYBR qPCR Master Mix	Vazyme Biotech Co.,Ltd	Cat# Q311-02
NanoTrans™ Transfection Reagent Plus	Cytoch	Cat# CT0005
DEPC H_2O	Beyotime	Cat# R0021
QuickBlock Immunostaining Blocking Solution	Beyotime	Cat#P0260
BSA	New Cell & Molecular Biotech Co.,Ltd	Cat#WB6504
Triton X-100	Sigma-Aldrich	Cat#X100
DAPI	ThermoFisher	Cat#D3571
Agrose	Gene fist	Cat#GF0100
SDS-PAGE Sample Loading Buffer，5X	Yeason	Cat# 20315E
Two-color prestained protein Marker 10 kDa~250 kDa	Shanghai Epizyme	Cat#WJ103
Cell Lysis Buffer for Western and IP without Inhibitors	New Cell & Molecular Biotech Co.,Ltd	Cat# P70100
Protease and Phosphatase Inhibitor Cocktail	New Cell & Molecular Biotech Co.,Ltd	Cat#P002
NCM Universal Antibody Diluent	New Cell & Molecular Biotech Co.,Ltd	Cat#WB500D
Protein A+G Agarose (Fast Flow,for IP)	Beyotime	Cat# P2055
BeyoMag™ Anti-HA Magnetic Beads	Beyotime	Cat# P2121
BeyoMag™ Anti-Flag Magnetic Beads	Beyotime	Cat# P2115
BeyoMag™ Anti-Myc Magnetic Beads	Beyotime	Cat#2218
Nuc Red Live 647 Ready Probes	Invitrogen	Cat#R37106
L- Glycine	AmBeed	Cat#A216141; CAS 56-40-6

THAM	AmBeed	Cat#A168222; CAS 77-86-1
SDS	AmBeed	Cat#A120709; CAS 151-21-3
Methanol	Aladdin	Cat#M433267; CAS 67-56-1
Ethanol	Aladdin	Cat#E111963; CAS 64-17-5
Xylene	Aladdin	Cat#X112051; CAS 1330-20-7
Poly-L-lysine	Macklin	Cat#P875129; CAS 25988-63-0
Histiocyte fixative solution	Leagene	Cat#DF0135
EDTA antigen retrieval solution (50×)	Solarbio	Cat# C1034
Ready-to-use normal goat serum	BOSTER	Cat#AR0009
PV-6000 Universal Kit (Mouse/Rabbit Polymer Assay System)	ZSGB-Bio	Cat#PV-6000
DAB kit	ZSGB-Bio	Cat#ZL1-9017
Eosin stain	Servicebio	Cat#G1002
Hematoxylin staining solution	Servicebio	Cat#G1004
Standard Matrigel	Corning	Cat#356234
Leukocyte Activation Cocktail, with BD GolgiPlug	BD Transduction Laboratories™	Cat#550583
Stain Buffer (FBS)	BD Transduction Laboratories™	Cat#554656
Neutral resin	Solarbio	Cat# G8590
Puromycin	Gibco	Cat#A1113803
Blasticidin S HCl	Beyotime	Cat#ST018
Kolliphor HS-15	Sigma-Aldrich	Cat#42966
**Critical Commercial Assays**		
Mouse IFN-beta Quantikine ELISA Kit	Bio-Techne China Co. Ltd.	Cat#DY466
Mouse IFN-gamma Quantikine ELISA Kit	Bio-Techne China Co. Ltd.	Cat#SMIF00
2'3'- Cyclic GAMP ELISA Kit	Arbor Assays	Cat# K067-H5
Mouse CXCL10/IP-10/CRG-2 DuoSet ELISA	Bio-Techne China Co. Ltd.	Cat#DY466
Mouse spleen lymphocyte isolate Kit	Solarbio	Cat#P8860
BCA protein assay kit	KeyGen Biotech	Cat#KGP903
Endotoxin-free plasmid mini-grape Kit	Genefist	Cat# GF2118
PAGE Gel Fast Preparation Kit	Shanghai Epizyme	Cat# PG112
One-Step PAGE Gel Fast Preparation Kit (15%)	Vazyme Biotech Co.,Ltd	Cat# E305-01
**Experimental models: Cell lines**		
L929 cells	ATCC	CCL-1
RAW264.7 cells	ATCC	TIB-71
DC2.4 cells	Pricella	CL-0545
CT26 cells	ATCC	CRL-2638
H22 cells	Pricella	CL-0341
HEK-293T cells	ATCC	CRL-3216
BMDCs cells	This paper	N/A
**Overexpressed or control cells**		
L929 cells stably expressing vector vehicle control	This paper	N/A
L929 cells stably expressing USP1-Flag	This paper	N/A

L929 cells stably expressing mCherry- Sting	This paper	N/A
L929 cells stably expressing HA-UB-WT	This paper	N/A
L929 cells stably expressing HA-UB-$K6_{only}$	This paper	N/A
L929 cells stably expressing HA-UB-$K11_{only}$	This paper	N/A
L929 cells stably expressing HA-UB-$K27_{only}$	This paper	N/A
L929 cells stably expressing HA-UB-$K29_{only}$	This paper	N/A
L929 cells stably expressing HA-UB-$K33_{only}$	This paper	N/A
L929 cells stably expressing HA-UB-$K48_{only}$	This paper	N/A
L929 cells stably expressing HA-UB-$K63_{only}$	This paper	N/A
L929 cells stably expressing HA-UB-$K27R_{only}$	This paper	N/A
**Recombinant DNA**		
pCDH-EF1-MCS-T2A-Puro vector	Laboratory of Dr. Ziyang Wang	N/A
psPAX2	Laboratory of Dr. Ziyang Wang	N/A
pMD2.G	Laboratory of Dr. Ziyang Wang	N/A
pCDH-EF1-MCS-T2A-Puro-USP1-Flag	Self-constructing plasmids	N/A
pCDH-EF1-MCS-T2A-Puro-Flag-mSTING-	Self-constructing plasmids	N/A
pCDH-EF1-MCS-T2A-Puro-mSTING-mCherry	Self-constructing plasmids	N/A
pCDH-EF1-MCS-T2A-Puro-HA-UB-WT	Self-constructing plasmids	N/A
pCDH-EF1-MCS-T2A-Puro-HA-UB-K6	Self-constructing plasmids	N/A
pCDH-EF1-MCS-T2A-Puro-HA-UB-K11	Self-constructing plasmids	N/A
pCDH-EF1-MCS-T2A-Puro-HA-UB-K27	Self-constructing plasmids	N/A
pCDH-EF1-MCS-T2A-Puro-HA-UB-K29	Self-constructing plasmids	N/A
pCDH-EF1-MCS-T2A-Puro-HA-UB-K33-specificity	Self-constructing plasmids	N/A
pCDH-EF1-MCS-T2A-Puro-HA-UB-K48	Self-constructing plasmids	N/A
pCDH-EF1-MCS-T2A-Puro-HA-UB-K63	Self-constructing plasmids	N/A
pCDH-EF1-MCS-T2A-Puro-HA-UB-K27R	Self-constructing plasmids	N/A
**Software and Algorithms**		
GraphPad Prism	GraphPad 8.0 Software	GraphPad
SPSS	SPSS 20.0 Software	SPSS
FlowJo	FlowJo V10	FlowJo
ImageJ	National Institutes of Health	ImageJ
FV31S-SW Version: 2.1.1.98	OLYMPUS	FV31S-SW
KViewer V1	KFBIO	KViewer
QuPath	QuPath-0.3.0 Software	QuPath

Bio-Rad CFX manager 3.1	Bio-Rad	CFX manager

Table S4: List of Abbreviations

Abbreviations	Full Terms
**TME**	Tumor microenvironment
**dsDNA**	Double-stranded DNA
**DUBs**	Deubiquitinating enzymes
**USP1**	Ubiquitin specific peptidase 1
**STING**	Stimulator of interferon genes protein
**cGAS**	Cyclic GMP-AMP synthase
**cGAMP**	Cyclic GMP-AMP
**TBK1**	TANK Binding Kinase 1
**IRF3**	Interferon regulatory factor 3
**ER**	Endoplasmic reticulum
**ERGIC**	ER-Golgi intermediate compartment
**SAR1A**	Secretion associated Ras related GTPase 1A
**SEC23**	Protein transport protein Sec23
**SEC24C**	Protein transport protein Sec24C
**SEC13**	Protein transport protein Sec13
**SEC31**	Protein transport protein Sec31
**COP-II**	Coat protein II
**Type I IFN**	Type I interferons
**RT**	Radiation therapy
**DAMPs**	Damage-associated molecular patterns
**APCs**	Antigen-presenting cells
**DCs**	Dendritic cells
**DSBs**	Double-strand breaks
**YIPF5**	Yip1 domain family member 5
**TMED2/5**	Transmembrane emp24 domain-containing protein 2/5
**iRhom2**	Inactive rhomboid protein 2
**STEEP**	STING ER exit protein
**PDL1**	Programmed cell death 1 ligand 1
**BMDCs**	Bone marrow-derived Dendritic Cells
**GM-CSF**	Granulocyte-macrophage Colony Stimulating Factor
**IF**	Immunofluorescence
**ip.**	Intraperitoneal injection
**K**	Lysine
**IHC**	Immunohistochemistry
**ANOVA**	Analysis of variance
**FCS**	Fetal calf serum
**USP2**	Ubiquitin Specific Peptidase 2

**USP5**	Ubiquitin Specific Peptidase 5
**USP7**	Ubiquitin Specific Peptidase 7
**USP8**	Ubiquitin Specific Peptidase 8
**USP9X**	Ubiquitin Specific Peptidase 9 X-Linked
**USP10**	Ubiquitin Specific Peptidase 10
**USP11**	Ubiquitin Specific Peptidase 11
**USP13**	Ubiquitin specific peptidase 13
**USP14**	Ubiquitin specific peptidase 14
**USP15**	Ubiquitin Specific Peptidase 15
**USP20**	Ubiquitin Specific Peptidase 20
**USP22**	Ubiquitin Specific Peptidase 22
**USP28**	Ubiquitin Specific Peptidase 28
**USP30**	Ubiquitin Specific Peptidase 30
**USP47**	Ubiquitin Specific Peptidase 47
**UCH-L1**	Ubiquitin C-Terminal Hydrolase L1
**UCH-L3**	Ubiquitin C-Terminal Hydrolase L3
**UCH-L5**	Ubiquitin C-Terminal Hydrolase L5
**BAP1**	Ubiquitin carboxyl-terminal hydrolase BAP1
**OTUB1**	OTU Deubiquitinase 1
**IFN β1**	Interferon β1
**DMSO**	Dimethyl sulfoxide
**EC50**	Median effect concentration
**CXCL10**	C-X-C Motif Chemokine Ligand 10
**LC3B**	Microtubule associated protein 1 light chain 3 Beta
**VPS34**	Vacuolar Protein Sorting 34
**HE staining**	Hematoxylin-eosin staining
**IHC**	Immunohistochemistry
**CRC**	Colorectal cancer
**MSCs**	Mesenchymal stem cells
**LARC**	Locally advanced rectal cancer
**TRG**	Tumor regression grade
**OS**	Overall survival
**DFS**	Disease-free survival
**NK cells**	Natural killer cell
**gDNA**	Genomic DNA
**mtDNA**	Mitochondrial DNA
**SAR1B**	Secretion associated Ras related GTPase 1B
**WDR48**	WD repeat-containing protein 48
**TAZ**	Tafazzin
**NLRP3**	NLR family pyrin domain containing 3
**SURF4**	Surfeit locus protein 4
**APOB**	Apolipoprotein B

**APOA1**	Apolipoprotein A1
**FFPE**	Formalin-fixed and paraffin-embedded
**VMAT**	Volumetric modulated arc therapy
**RT-qPCR**	Reverse transcription quantitative polymerase chain reaction
**CD3e**	T-Cell Surface Glycoprotein CD3 Epsilon Chain
**CD4**	T-cell surface glycoprotein CD4
**CD8a**	T-cell surface glycoprotein CD8 alpha chain
**CD11c**	Integrin alpha-X
**Th17**	T helper cell 17
**RNF26**	E3 ubiquitin-protein ligase RNF26
**RNF5**	E3 ubiquitin-protein ligase RNF5
**CD45**	Leukocyte common antigen
**Ly6C**	Lymphocyte antigen 6 complex
**SYSUFAH**	The First Affiliated Hospital, Sun Yat-sen University

Supplementary Experimental Section

Extraction and Culturing of BMDC Cells

Harvest the femurs and tibias from 4 to 12-week-old Balb/c female mice, using forceps to remove the connective tissue. Afterwards, immerse the intact bones in 70% ethanol for 5 minutes for disinfection, and then wash them with PBS. Then, cut both ends with scissors, and flush the bone marrow with PBS using an injection syringe with a 0.45 mm diameter needle, collecting the PBS. After passing through a 100 mm cell strainer to remove bone fragments, centrifuge at 1500 rpm for three minutes to obtain the total bone marrow cells. After lysing the red blood cells with red cell lysing buffer one to two times, and then centrifuge at 1500 rpm f or three minutes to obtain the total white blood cells in the bone marrow. After counting, 2×10^6 cells were plated in a 10 cm dish (10 mL of R10 medium containing 100 U/ml rmGM-CSF (PeproTech, no. 315-03-20) per dish). The R10 medium is a RPMI 1640 basal medium supplemented with 1% penicillin and streptomycin; 2 mM L-glutamine, 50 mM 2-mercaptoethanol, and 10% of heat-inactivated and 0.22 mm filtered Millipore fetal calf serum (FCS) for incubation at 37 °C with 5% CO_2. After three days, 10 mL of fresh R10 medium containing 100 U/mL rmGM-CSF was added. On the 6th and 8th days, half of the culture supernatant was collected, centrifuged at 1500 rpm for 3 minutes, and resuspended in 10 mL of fresh R10 medium containing 200 U/mL rmGM-CSF. The cell suspension was then returned to the original dish. Co-culture experiments were performed in vitro from the 8th to the 10th day.

Flow cytometry analysis

Dissect the mice to isolate the spleen. After grinding the spleen, resuspend it in PBS buffer, and then isolate the mouse spleen cells using 1× Lymphocyte Separation Medium. Next, add 50 μL of the cell suspension, 1 mL of RPMI 1640 culture medium, and 2 μL of the stimulation blocker to a 24-well cell culture plate. Incubate the plate at 37°C for 8 hours.

After incubation, centrifuge the samples to collect the cells and discard the supernatant. Add red blood cell lysis buffer to the cell pellet and let it lyse for 3 minutes, then add stain buffer to terminate

the lysis. Centrifuge again, discard the supernatant, and wash the cells with PBS buffer.

For sample preparation, include a blank tube that does not contain antibodies. For the live/dead single staining tube, add 100 μL of PBS and FVS dye (1:1000 dilution) to a tube containing 1×10^6 cells in suspension, and stain in the dark at 4 °C for 20 minutes. For the single antibody staining tubes, add 1 μg of antibodies (CD3, CD4, CD8) and stain in the dark at 4 °C for 20 minutes.

Perform surface staining by adding 100 μL of the 1:1000 FVS (1 μL AF700 + 1 mL PBS) live/dead dye solution in 1× PBS (without serum). Incubate in the dark at 4 °C for 20 minutes, then wash the cells with stain buffer. Centrifuge to discard the wash solution and resuspend the cells in stain buffer (containing FBS).

Dilute the antibodies (CD3, CD4, CD8) with stain buffer and add them to each tube (containing 1 μg of each antibody). Incubate at room temperature in the dark for 15 minutes. Wash with PBS, then centrifuge to remove any unbound antibodies. Finally, analyze the samples using the CytoFLEX S (BECKMAN COULTER).

To prepare single-cell suspensions, tumor tissues were minced into small pieces and mechanically dissociated using gentleMACS™ Dissociators (Miltenyi Biotech). The tissues were then enzymatically digested with 1 mg/mL collagenase IV (Sigma) and 0.2 mg/mL DNase I (Sigma) for 30 minutes at 37 °C. For staining, the single-cell suspensions were first blocked with anti-FcR antibodies (clone 2.4G2, BioXcell) and subsequently stained with antibodies targeting CD11c, Ly6C, and CD45. Cell sorting and analysis were performed using a FACSAria II Cell Sorter (BD). Extract RNA from the sorted lymphocytes for RT-qPCR analysis.
